# Second Spectrum of Tungsten (W II)

**DOI:** 10.6028/jres.068A.021

**Published:** 1964-04-01

**Authors:** Donald D. Laun

## Abstract

A preliminary report on the second spectrum of tungsten, published in 1938, presented 27 even energy levels and 50 odd energy levels that were derived from 500 W II lines ranging in wavelength from 1961.43 Å to 4348.13 Å. The present paper submits data on 62 even levels, 132 odd levels, and 2,173 classified lines of W II, ranging in wavelength from 1756.6 Å to 6219.77 Å. The ground state of the W^+^ ion is represented by the ^6^D_0½_ level of a sextet D term arising from the 5*d*^4^6*s*^1^ electron configuration, but the level intervals and magnetic splitting factors indicate considerable departure from *LS*-coupling, suggesting that coupling intermediate between *LS* and *Jj* may be more appropriate for the spectrum W II.

## 1. Introduction

The search for regularities among spectral lines emitted by tungsten arcs and sparks was initiated at the National Bureau of Standards in 1925 when O. Laporte and I were both new (temporary) members of the Spectroscopy Section. By exploiting existing data on wavelengths, intensities, and Zeeman patterns, Laporte [1]^2^ identified the first spectral terms and classified lines of W I. Zeeman data confirmed the designation of the six lowest energy levels as ^5^D from 5*d*^4^6*s*^2^ and ^7^S from 5*d*^5^6*s*^1^. This term analysis and quantum interpretation of W I was later greatly extended by Laporte and Mack [2] until it included 300 energy levels and 2,378 classified lines ranging in wavelength from 2008.64 Å to 11477.97 Å.

Shortly after Laporte found the low-energy terms in W I, I began a search for the theoretical low ^6^D (from 5*d*^4^6*s*^1^) and ^6^S (from 5*d*^5^) terms in W II, assuming that ionization removed one *s* electron. This initial search failed because of insufficient information about the spectrum W II. Then I undertook to reobserve the ultraviolet arc and spark spectra of tungsten, to distinguish W II from W I, to refine the wavelengths, and to observe Zeeman patterns of W II lines. From these observations, 27 even levels (including ^6^D and ^6^S) and 50 odd levels were derived from 500 W II lines ranging in wavelength from 1961.43 Å to 4348.13 Å; these results were published [3] in 1938 as a preliminary report on lines and levels of the spectrum W II. From time to time during the following quarter century, further improvements in observations and extensions of analyses have been made ; the purpose of the present paper is to present the final data on 62 even levels, 132 odd levels, and 2,173 classified lines of W II, ranging in wavelength from 1756.6 Å to 6219.77 Å.

## 2. Experimental Procedure

Small rods of very pure tungsten were used as electrodes in the arcs and sparks that served as light sources. The arcs were operated with applied potential of 220 v and 6 amp dc, the sparks with 30,000 v, about 30 ma, ac, and 0.006 *μ*f capacitance.

The first spectrograms were made in 1925 with a Hilger E 1 (Littrow) quartz spectrograph which could be effectively employed in the ultraviolet down to wavelength 1900 A. Later, these spectrograms were supplemented by a new series obtained by C. C. Kiess with the first Hilger E 185 (Littrow) quartz spectrograph that was delivered to the Bureau of Standards in 1932. This larger spectrograph has a focal length of 3 m, and reciprocal dispersion, or plate factor, of 0.3 Å/min at 2100 Å and 1 Å/mm at 3000 A. These observations of tungsten lines on photographic plates exposed in quartz spectrographs were presented by me as a Master’s Thesis at the University of Chicago [4]. Additional spectrograms were made in 1933 by Kiess with a concave grating of 22-ft radius, and 30,000 lines per inch. In a Wadsworth mounting, this grating produced spectra down to 1900 Å with a plate factor of 2.44 Å/mm in the first order, and with a plate factor of 0.88 Å/mm in the second order from 3200 to 4400 Å. This grating spectrograph was employed also to photograph tungsten spectra throughout the visible range.

Since all the above-mentioned spectrographs were stigmatic, movable diaphragms before the slits permitted placing strips of juxtaposed spectra on photographic plates. The usual procedure was to photograph the iron-arc spectrum containing standard wavelengths, then the tungsten arcs and sparks in adjacent strips and another iron spectrum beside the tungsten spark. The positions of tungsten spectral lines relative to iron lines were measured on Gaertner comparators, in 1925–26 at the Bureau of Standards; and in 1933–34 at Marquette University by courtesy of the late Fr. Joseph Carroll, S. J. Linear scale readings allowed the wavelengths of tungsten lines to be interpolated between iron standards in the case of prismatic spectra with the aid of Hartmann’s dispersion formula, and from grating spectrograms by assuming linear dispersion. In both cases, small corrections were made when the interpolated values of iron wavelengths departed from their standard values. For purposes of analysis, the final wavelengths of tungsten lines were converted to vacuum wavenumbers with the aid of the Table of Wavenumbers published by Coleman, Bozman, and Meggers [5].

At the same time that the positions or wavelengths of tungsten lines were being measured, their relative intensities were estimated in both sources. Although this intensity scale, ranging from 1 for a barely discernible line to 1000 for the strongest, is more or less arbitrary, it is primarily useful in separating W II from W I, and also qualitatively supports the subsequent analysis in which intensities are related to quantum numbers. In some cases W II lines appear to coincide with W I, and if a W II line sometimes appears stronger in the arc than in the spark, it may be an example of pole effect. Unfortunately, no deliberate attempt to exclude or exploit pole effect in the tungsten arc was made.

Finally, some information about tungsten spectra in the vacuum ultraviolet was obtained by measuring two spectrograms made by J. C. Boyce at the Massachusetts Institute of Technology in 1940 and kindly presented to the Bureau of Standards. These spectrograms were obtained with an evacuated grating spectrograph having a plate factor of 4.26 Å/mm. The spectra were measured down to 1200 Å. Although the excitation conditions were altered, the differences between spectra were so slight that only above 1756 Å was it possible positively to identify W II lines. The wavelengths from this point to 1950 Å were finally corrected to the scale of values calculated from atomic energy levels.

My preliminary paper [3] of 1938 reported Zeeman-effect measurements of spectrograms made with a Weiss magnet and quartz spectrographs at the Bureau of Standards. These data have been superseded by those obtained with the more powerful Bitter magnet and grating spectrographs at the Massachusetts Institute of Technology. These results will be mentioned in the next section.

## 3. Results

The main result of the experiments mentioned above is that the wavelengths of about 13,000 spectral lines characteristic of arcs and sparks between tungsten electrodes have been measured. Among these, about 4,000 have been attributed to W II and 2,173 of these lines, including 90 percent of the total intensity, have been shown to arise from transitions between 194 derived energy levels, 62 even and 132 odd. The even levels are displayed in table 1 and the odd levels in table 2. In these tables, the electron configuration is given in column 1, the spectral term designation in column 2, total angular momentum, *J*, in column 3, relative values of energy levels in column 4, intervals between levels of complex terms in column 5, and observed magnetic splitting factor, *g*, in column 6. Most of these data were supplied in 1956 for the third volume of Atomic Energy Levels [6] which contains *J* and level values for 45 even and 90 odd levels. In that volume, *g*-factors were reported for 38 even levels and for 58 odd. These were derived from MIT Zeeman spectrograms measured by J. E. Mack and Mrs. Taschek at the University of Wisconsin. Since 1956, the number of even levels has increased from 45 to 62 and odd levels from 90 to 132. Likewise, *g*-factors are now available for 46 even and 71 odd levels as compared with 38 and 58 respectively, in 1956.

In Atomic Energy Levels [6], it is stated that because of departure from *LS*-coupling it is difficult to assign term designations except for the lowest terms. Consequently, only six even terms, comprising 21 levels between 0 and 17437 K, were completely grouped and designated, and 24 higher even levels, between 18000 and 26929 K, were tentatively designated, guided by analogy with Mo II, *g*-factors, and theoretical calculations of the late R. E. Trees. Likewise, two odd terms, comprising six levels, were tentatively designated ^6^F and ^2^S, but the remaining 84 levels were given as miscellaneous. In the present paper, additional levels and *g*-factors are reported but no revision or extension of designations has been made.

The spectral lines of tungsten from which all the information in tables 1 and 2 was derived, are listed in table 3 in order of increasing wavelength. Each line is described by its measured wavelength in angstroms, by its estimated intensity in a tungsten arc and/or spark, by its appropriate vacuum wavenumber in kaysers (1 K=1 cm^−1^), by the difference (*O–C*) between the observed wavenumber and that calculated from the combination of energy levels shown in the next-to-last column. Abbreviated notes about the observed Zeeman patterns for 307 lines appear in the final column.

Table 3 contains 2,173 classified lines of W II, including 42 doubly classified. The only other table of classified W II lines was published [3] in 1938; it contained 500 lines, including 6 doubly classified. In addition to 2,173 classified lines, table 3 also contains wavelengths, intensities, and wavenumbers of 25 of the strongest W II lines still unclassified; these are included with the hope that the blank spaces under “combinations” may eventually be filled in.

The “Intensity” column of table 3 shows that the great majority of these W II lines were observed in arc as well as spark sources but with generally higher intensity in the latter when spectrogram exposures were chosen to make W I lines appear in both sources with nearly equal intensities. In a few cases, W II lines appear only, or stronger, in arc spectra; most if not all such deviations may be explained by coincident W I lines, pole effects, or air lines that occasionally mask metal lines in sparks operated at atmospheric pressure. Where the letter *A* follows an arc intensity, a line with nearly identical wavelength has actually been classified as a combination of W I energy levels. The letter *d* in table 3 indicates a double line.

The observed minus calculated wavenumbers (*O–C*) have an average value of 0.1 K for wavelengths measured in air, that is, above 2000 Å. This means that the average error in these wavelength measurements is of the order of 0.01 Å, and there is high confidence that the great majority of the combinations are real, rather than accidental. However, in the vacuum ultraviolet, below 2000 Å, the average *O–C* is considerably greater, in a few cases as large as 5 K, which entails an error in wavelength of the order of 0.2 Å. In such cases, confidence that the combinations are genuine is greatly reduced but they have been tentatively retained for the following reasons. Since the scale of wavenumbers is greatly compressed in the vacuum region, and the tungsten spectra below 2000 Å were photographed with relatively small dispersion and measured without adequate standard wavelengths, it was inevitable that these measured wavelengths of W II lines would be more or less uncertain. Furthermore, it appears that most of these WII short waves involve the lowest and adjacent metastable energy levels, which is precisely what should be expected of strong lines with high energy. Therefore, these combinations with relatively large *O–C* may be regarded as real pending wavelength measurements of higher accuracy.

In the introduction, I mentioned that the first spectral terms, both in WI and in WII, were found from Zeeman-effect observations. In both spectra, such observations have greatly facilitated their analyses, and constitute the supreme test of validity. In the last column of table 3, are found some notes about the observed Zeeman patterns of 307 WII lines extending from 2216 Å to 4358 Å. The Zeeman patterns of W I and W π on MIT spectrograms were measured at the University of Wisconsin by J. E. Mack and Mrs. Taschek who supplied most of the magnetic splitting factors (*g*-factors) for tungsten levels that appear in Atomic Energy Levels [6]. Recently, those spectrograms were kindly loaned to me for examination. Without giving any details, I have merely written “res” to indicate resolved Zeeman patterns that agree with the combinations of W II lines in table 3. Unresolved Zeeman patterns are numbered 4, 5, 6, and 7, according to the notation of Back and Laudé [7]. Incidentally, Zeeman types 4, 5, and 6 uniquely characterize, and fix *J*-values of, energy levels belonging to even multiplicities such as those responsible for WII lines. All the Zeeman- effect spectrograms are thickly covered by patterns of W I lines, and the W II patterns are often obscured, or vice versa, but despite these difficulties, the combinations are generally verified.

Finally, reference is made to the Tables of Spectral- Line Intensities by Meggers, Corliss, and Scribner [8] which list 1,300 tungsten lines, 1168 W I and 132 W II, observed in an arc between copper electrodes containing 0.1 atomic percent of tungsten. It is noteworthy that no WII lines appear among the first 28 lines of highest intensity; they are all W I. Tungsten is similar to rhenium in this respect, but unlike thorium or uranium in which spark lines predominate in this type of arc source. Ionization potentials may be responsible for this difference, since the I.P. of copper is less than that of tungsten or rhenium but greater than that of thorium or uranium. In the list of all observed lines of tungsten [8], all of the W II lines are now classified, and will be given in this paper.

In conclusion, let me say that since my association with the Bureau of Standards in 1925–26, when this investigation began, and again in 1943–44, when I collaborated on the uranium project [9], this work on W II has been pursued as a part-time occasional avocation with the goal of finding order in a complex spectrum.

## Figures and Tables

**Table 1:** Even levels of W *II*

Configuration	Designation	*J*	Level	Interval	Obs. *g*
					
5*d*^4^(^5^D)6*s*	*a*^6^D	0½	0.00		3.186
				1518.78	
		1½	1518.78		1.839
				1653.74	
		2½	3172.52		1.639
				1543.80	
		3½	4716.32		1.563
				1430.84	
		4½	6147.16		1.522
5*d^5^*	*a*^6^S	2½	7420.43		1.913
5*d*^4^(^3^F)6*s*	*a*^4^F	1½	8711.26		0.624
				2589.82	
		2½	11301.08		1.084
				2110.88	
		3½	13411.96		1.186
				1445.26	
		4½	14857.22		1.234
5*d*^4^(^3^P)6*s*	*a*^4^P	0½	8832.66		2.383
				1759.86	
		1½	10592.52		1.471
				2841.58	
		2½	13434.10		1.526
5*d*^4^(^5^D)6*s*	*a*^4^D	0½	13173.38		0.455
				1460.98	
		1½	14634.36		1.183
				333.46	
		2½	14967.82		1.013
				179.20	
		3½	15147.02		0.872
5*d*^4^(^3^G)6*s*	a^4^G	2½	16234.84		0.995
				354.83	
		3½	16589.67		1.153
				−36.53	
		4½	16553.14		1.137
				883.88	
		5½	17437.02		1.181
5*d*^4^(^3^H)6*s*	^4^H	3½	18000.70		1.098
5*d*^5^	^2^D	1½	18990.96		0.90
5*d*^5^	^4^G	4½	19070.68		1.102
5*d*^5^	^4^G	2½	19276.52		0.997
5*d*^4^(^3^P)6*s*	^2^P	0½	19404.08		0.64
5*d*^4^(^3^H)6*s*	^4^H	6½	19442.54		
5*d*^5^	^2^D	*2*½	19637.38		1.102
5*d*^5^	^4^G	3½	20039.74		1.107
5*d*^5^	^4^D	1½	20455.93		0.51
5*d*^5^	^4^G	5½	20534.35		1.197
5*d*^4^(^3^H)6*s*	^4^H	4½	20780.38		1.065
5*d*^5^	^4^F	2½	22139.97		1.06
5*d*^5^	^4^F	3½	22194.08		1.119
5*d*^4^(^3^P)6*s*	^2^P	1½	22503.06		1.22
		0½	22535.80		2.2
5*d*^4^(^3^D)6*s*	^4^D	3½	23046.80		0.86
5*d*^5^	^4^F	4½	23234.87		1.249
5*d*^5^	^4^D	2½	23450.50		1.297
5*d*^5^	^4^D	3½	23803.84		
5*d^5^*	^2^I	5½	23955.40		1.10
		3½	24804.67		1.10
		1½	24918.10		
5*d*^5^	^4^F	1½	24991.56		0.9
		0½	25045.20		0.32
5*d*^5^	^4^P	1½	25169.87		1.64
		4½	25209.28		
		2½	25672.16		0.9
5*d*^4^(^3^H)6*s*	^2^H	4½	26158.74		
5*d*^5^	^4^P	2½	26227.00		1.04
5*d*^5^(^3^H)6*s*	^2^H	*5*½	26929.34		
		3½	27273.86		
		2½	28118.90		
		6½	28187.60		
		5½	28377.80		
		1½	28491.00		
		3½	28631.86		
		4½	29341.56		
		4½	30633.02		
		5½	31100.46		
		*5*½	33910.56		1.34
		4½	34091.02		

**Table 2:** Odd levels of *W II*

Configuration	Designation	*J*	Level	Interval	Obs. g
					
5*d*^4^(^5^D)^6^*p*	*z*^6^F°	0½	*36165.35*		0.678
				2964.06	
		1½	*39129.41*		1.147
				2920.04	
		2½	*42049.45*		1.292
				2827.73	
		3½	*44877.18*		1.277
				1616.25	
		4½	*46493.48*		1.311
				5001.57	
		5½	*51495.00*		1.054
5*d*^4^(^3^P)6*p*	*z*^2^S°	0½	*38576.32*		1.614
		2½	*39936.81*		0.889
		1½	*42298.20*		1.498
		3½	*42390.27*		1.161
		2½	*44354.82*		1.390
		0½	*44455.18*		−0.217
		4½	*44758.10*		1.270
		1½	*44911.63*		1.221
		0½	*45457.02*		0.519
		1½	*45553.70*		1.033
		3½	*46175.42*		1.452
		2½	*46355.40*		1.236
		0½	*46625.27*		1.70
		1½	*47179.94*		1.007
		2½	*47413.33*		1.111
		1½	*47588.64*		2.00
		2½	*48284*.*48*		1.366
		5½	*48332.73*		
		3½	*48830.70*		1.008
		1½	*48982.86*		1.72
		3½	*49124.52*		1.499
		0½	*49154*.*50*		2.78
		4½	*49181.04*		1.409
		2½	*49242.10*		1.510
		2½	*50292.33*		1.334
		1½	*50430.95*		0.93
		4½	*50863.05*		1.194
		3½	*51045.25*		
		1½	*51254*.*40*		1.58
		2½	*51438.03*		1.301
		3½	*51863.03*		0.937
		2½	*52087.02*		
		3½	*52275.28*		1.297
		0½	*52355.11*		0.981
		4½	*52567.15*		
		0½	*52593.72*		1.56 ?
		1½	*52803.00*		
		3½	*52901.74*		1.374
		2½	*53113.52*		1.262
		1½	*53329.71*		1.357
		3½	*53338.07*		0.968
		4½	*53369.97*		1.086
		1½	*53422.98*		0.976
		0½	*53440.17*		2.038
		2½	*54026.24*		
		4½	*54056.54*		1.123
		1½	*54137.20*		1.608
		5½	*54229.06*		
		2½	*54375.82*		1.51
		0½	*54485.60*		1.46
		3½	*54498.57*		
		2½	*54704.61*		0.623
		0½	*54760.06*		
		5½	*54958.58*		1.141
		3½	*55022.86*		
		2½	*55162.30*		1.00
		4½	*55392.37*		1.061
		1½	*55488.01*		
		1½	*56084.*3*0*		1.021
		5½	*56376.45*		
		4½	*56413.64*		
		6½	*56439.60*		
		2½	*56544.40*		
5*d*^4^(^3^P)6*p*	*z*^2^S^0^	3½	*56612.74*		1.22
		3½	*50768.61*		1.147
		2½	*56874.99*		0.815
		1½	*56932.27*		1.06
		4½	*57089.46*		
		2½	*57252.00*		
		3½	*57729.92*		1.184
		2½	*57856.70*		1.36
		4½	*57986.92*		
		1½	*58007.60*		1.20
		2½	*58336.98*		
		4½	*58687.88*		
		3½	*58709.56*		
		1½	*58747.94*		0.78
		5½	*58891.74*		1.144
		3½	*59276.81*		1.102
		4½	*59399.34*		1.179
		1½	*59816.30*		
		3½	*59869.14*		1.125
		3½	*59933.66*		
		2½	*59992.20*		
		5½	*60218.84*		1.130
		3½	*60256.45*		
		4½	*60278.71*		
		3½	*60424.14*		
		2½	*60474.67*		
		2½	*60656.51*		
		2½	*60900.97*		0.92
		4½	*61055.80*		
		5½	*61240.81*		1.120
		3½	*61326.29*		
		4½	*61360.54*		
		3½	*61550.60*		
		2½	*61566.70*		1.07
		5½	*61589.46*		1.149
		6½	*61602.18*		
		2½	*62333.20*		
		4½	*62437.04*		
		6½	*62714.56*		
		4½	*62715.98*		
		5½	*62966.50*		
		2½	*62989.60*		
		6½	*63087.90*		
		3½	*63266.30*		
		3½	*63788.20*		
		2½	*63880.10*		
		2½	*64030.34*		
		4½	*64207.50*		
		2½	*64310.00*		
		3½	*64356.70*		
		4½	*64516.37*		
		3½	*64896.22*		
		5½	*64969.10*		
		2½	*64990.32*		
		4½	*65003.20*		
		2½	*65141.56*		
		5½	*65326.40*		
		3½	*65644.00*		
		5½	*65684.80*		
		4½	*66270.95*		
		5½	*66703.50*		
		5½	*68012.50*		
		6½	*68078.98*		

**Table 3:** Classified lines of *W II*

Wavelength (Å)	Intensity		Wave number (cm^−1^)		Combination	Zeeman effect
	
	Arc	Spark	Observed	*O–C*		
						
Vac.						
1756.604		8	56928.0	−4.3	*a* ^6^D_0½_— $56932_{1½}^{{}^{\circ}}$	
1757.789		3	56889.6	0.0	*a* ^6^S_2½_— $64310_{2½}^{{}^{\circ}}$	
1759.408		4	56837.3	+3.0	*a* ^6^D_3½_— $61550_{3½}^{{}^{\circ}}$	
1760.00		5	56818.2	0.0	*a* ^6^D_1½_— $58336_{2½}^{{}^{\circ}}$	
1765.514		20	56640.7	$\left\{ \begin{array}{l} -3.1\\ -3.5 \end{array}\right.$	*a* ^6^D_2½_— $59816_{1½}^{{}^{\circ}}$ *a* ^6^D_3½_— $61360_{4½}^{{}^{\circ}}$	
1766.319		8	56614.9	$\left\{ \begin{array}{l} -4.9\\ -5.0 \end{array}\right.$	*a* ^6^D_3½_— $61326_{3½}^{{}^{\circ}}$ *a* ^6^S_2½_— $64030_{2½}^{{}^{\circ}}$	
1775.023		3	56337.3	−0.6	*a* ^6^D_1½_— $57856_{2½}^{{}^{\circ}}$	
1776.834		4	56279.9	+0.8	*a* ^6^F_1½_— $64990_{2½}^{{}^{\circ}}$	
1783.050		10	56083.7	−0.6	*a* ^6^D_0½_— $56084_{1½}^{{}^{\circ}}$	
1794.166		4	55736.2	+3.0	*a* ^6^D_1½_— $57252_{2½}^{{}^{\circ}}$	
1795.131		20	55706.2	−1.6	*a* ^6^D_3½_— $60424_{3½}^{{}^{\circ}}$	
1798.60		3	55598.8	+0.1	*a* ^4^F_1½_— $64310_{2½}^{{}^{\circ}}$	
1799.558		4	55569.2	0.0	*a* ^4^S_2½_— $62989_{2½}^{{}^{\circ}}$	
1800.570		20	55538.0	$\left\{ \begin{array}{l} +1.0\\ -2.1 \end{array}\right.$	*a* ^6^D_2½_— $58709_{3½}^{{}^{\circ}}$ *a* ^6^D_3½_— $60256_{3½}^{{}^{\circ}}$	
1802.211		8	55487.4	−0.6	*a* ^6^D_0½_— $55488_{1½}^{{}^{\circ}}$	
1803.840		8	55437.3	−5.0	*a* ^6^D_4½_— $61589_{5½}^{{}^{\circ}}$	
1804.51		6	55416.7	+3.2	*a* ^6^D_1½_— $56932_{1½}^{{}^{\circ}}$	
1806.437		3	55357.6	+1.4	*a* ^6^D_1½_— $56874_{2½}^{{}^{\circ}}$	
1807.645		2	55320.5	+1.4	*a* ^4^F_1½_— $64030_{2½}^{{}^{\circ}}$	
1809.082		2	55276.6	+0.7	*a* ^6^D_3½_— $59992_{2½}^{{}^{\circ}}$	
1811.021		5	55217.5	+0.2	*a* ^6^D_3½_— $59933_{3½}^{{}^{\circ}}$	
1812.161		40	55182.7	+3.6	*a* ^6^D_4½_— $61326_{3½}^{{}^{\circ}}$	
1812.765		6	55164.4	−0.1	*a* ^6^D_2½_— $58336_{2½}^{{}^{\circ}}$	
1812.977		5	55157.9	+5.1	*a* ^6^D_3½_— $59869_{3½}^{{}^{\circ}}$	
1815.073		2	55094.2	+0.6	*a* ^6^D_4½_— $61240_{5½}^{{}^{\circ}}$	
1817.347		10	55025.2	−0.4	*a* ^6^D_1½_— $56544_{2½}^{{}^{\circ}}$	
1821.062		30	54913.0	$\left\{ \begin{array}{l} +4.4\\ -0.2 \end{array}\right.$	*a* ^6^D_4½_— $61055_{4½}^{{}^{\circ}}$ *a* ^6^S_2½_— $62333_{2½}^{{}^{\circ}}$	
1823.64		3	54835.4	+0.3	*a* ^6^D_2½_— $58007_{1½}^{{}^{\circ}}$	
1826.126		20	54760.7	+0.6	*a* ^6^D_0½_— $54760_{0½}^{{}^{\circ}}$	
1828.69		6	54684.0	$\left\{ \begin{array}{l} +1.0\\ -0.2 \end{array}\right.$	*a* ^6^D_3½_— $59399_{4½}^{{}^{\circ}}$ *a* ^6^D_2½_— $57856_{2½}^{{}^{\circ}}$	
1838.338		25	54397.0	−0.8	*a* ^4^P_1½_— $64990_{2½}^{{}^{\circ}}$	
1847.041		5	54140.6	+3.4	*a* ^6^D_0½_— $54137_{1½}^{{}^{\circ}}$	
1848.104		30	54109.7	+0.4	*a* ^6^D_4½_— $60256_{3½}^{{}^{\circ}}$	
1852.110		15	53992.5	−0.7	*a* ^6^D_3½_— $58709_{3½}^{{}^{\circ}}$	
1852.917		20	53969.0	−0.2	*a* ^6^D_1½_— $55488_{1½}^{{}^{\circ}}$	
1860.069		4	53761.4	+1.6	*a* ^6^D_2½_— $56932_{1½}^{{}^{\circ}}$	
1861.26		6	53727.0	+5.0	*a* ^6^D_4½_— $59869_{3½}^{{}^{\circ}}$	
1864.976		15	53620.0	−0.7	*a* ^6^D_3½_— $58336_{2½}^{{}^{\circ}}$	
1865.818		8	53595.8	−0.3	*a* ^6^D_2½_— $56768_{3½}^{{}^{\circ}}$	
1871.261		6	53439.9	$\left\{ \begin{array}{l} -0.3\\ -0.3 \end{array}\right.$	*a* ^6^D_0½_— $53440_{0½}^{{}^{\circ}}$ *a* ^6^D_2½_— $56612_{3½}^{{}^{\circ}}$	
1871.90		6	53421.6	−1.4	*a* ^6^D_0½_— $53422_{1½}^{{}^{\circ}}$	
1873.646		20	53371.9	0.0	*a* ^6^D_2½_— $56544_{2½}^{{}^{\circ}}$	
1875.156		12	53328.9	−0.8	*a* ^6^D_2½_— $53329_{1½}^{{}^{\circ}}$	
1877.33		2	53267.2	−3.4	*a* ^6^D_3½_— $57986_{4½}^{{}^{\circ}}$	
1877.869		20	53251.8	−0.4	*a* ^6^D_4½_— $59399_{4½}^{{}^{\circ}}$	
1878.54		4	53233.0	−3.1	*a* ^6^S_2½_— $60656_{2½}^{{}^{\circ}}$	
1880.216		8	53185.4	−0.4	*a* ^6^D_1½_— $54704_{2½}^{{}^{\circ}}$	
1881.798		15	53140.6	+0.2	*a* ^6^D_3½_— $57856_{2½}^{{}^{\circ}}$	
1882.185		8	53129.7	+0.1	*a* ^6^D_4½_— $59276_{3½}^{{}^{\circ}}$	
1886.304		6	53013.7	+0.1	*a* ^6^D_3½_— $57729_{3½}^{{}^{\circ}}$	
1891.908		6	52856.7	$\left\{ \begin{array}{l} -0.3\\ +1.3 \end{array}\right.$	*a* ^6^D_1½_— $54375_{2½}^{{}^{\circ}}$ *a* ^6^F_1½_— $61566_{2½}^{{}^{\circ}}$	
1892.442		15	52841.8	+5.8	*a* ^6^S_2½_— $60256_{3½}^{{}^{\circ}}$	
1893.80		4	52803.9	+0.9	*a* ^6^D_0½_— $52803_{1½}^{{}^{\circ}}$	
1895.943		30	52744.2	−0.4	*a* ^6^D_4½_— $58891_{5½}^{{}^{\circ}}$	
1900.503		12	52617.6	−0.8	*a* ^6^D_1½_— $54137_{1½}^{{}^{\circ}}$	
1901.245		30	52597.1	+3.4	*a* ^6^D_0½_— $52593_{0½}^{{}^{\circ}}$	
1902.51		20	52562.1	−0.3	*a* ^6^D_4½_— $58709_{3½}^{{}^{\circ}}$	
1906.677		12	52447.3	−1.4	*a* ^6^S_2½_— $59869_{3½}^{{}^{\circ}}$	
1908.750		10	52390.3	−5.6	*a* ^6^S_2½_— $59816_{1½}^{{}^{\circ}}$	
1909.16		6	52379.0	+5.9	*a* ^6^D_3½_— $57089_{4½}^{{}^{\circ}}$	
1909.80		4	52361.4	+6.3	*a* ^6^D_0½_— $52355_{0½}^{{}^{\circ}}$	
1911.50		25	52314.9	−0.6	*a* ^6^D_2½_— $55488_{1½}^{{}^{\circ}}$	
1915.38		8	52209.0	−0.9	*a* ^4^P_2½_— $65644_{3½}^{{}^{\circ}}$	
1925.99		12	51921.3	−0.1	*a* ^6^D_1½_— $53440_{0½}^{{}^{\circ}}$	
1928.61		20	51850.8	+0.5	*a* ^6^D_2½_— $55022_{3½}^{{}^{\circ}}$	
1929.47		10	51827.7	−0.4	*a* ^6^D_3½_— $56544_{2½}^{{}^{\circ}}$	
1930.10		6	51810.8	−0.1	*a* ^6^D_1½_— $53329_{1½}^{{}^{\circ}}$	
1931.87		10	51763.3	−0.1	*a* ^4^F_1½_— $60474_{2½}^{{}^{\circ}}$	
1934.345		10	51697.1	−0.2	*a* ^6^D_3½_— $56413_{4½}^{{}^{\circ}}$	
1934.62		3	51689.7	+1.2	*a* ^4^F_2½_— $62989_{2½}^{{}^{\circ}}$	
1938.21		25	51594.0	−0.7	*a* ^6^D_1½_— $53113_{2½}^{{}^{\circ}}$	
1938.74		3	51579.9	$\left\{ \begin{array}{l} -2.9\\ +1.5 \end{array}\right.$	*a* ^6^D_4½_— $57729_{3½}^{{}^{\circ}}$ *a* ^4^F_3½_— $64990_{2½}^{{}^{\circ}}$	
1940.54		8	51532.0	−0.1	*a* ^6^D_2½_— $54704_{2½}^{{}^{\circ}}$	
1943.27		4	51459.6	+0.2	*a* ^4^G_4½_— $68012_{5½}^{{}^{\circ}}$	
1945.02		10	51413.5	−0.2	*a* ^4^F_4½_— $66270_{4½}^{{}^{\circ}}$	
1948.35		30	51325.5	−0.5	*a* ^6^D_2½_— $54498_{3½}^{{}^{\circ}}$	
1949.55		40	51293.9	+4.8	*a* ^6^S_2½_— $58709_{3½}^{{}^{\circ}}$	
1949.93		4	51283.9	−0.3	*a* ^6^D_1½_— $52803_{1½}^{{}^{\circ}}$	
1951.06		40	51254.2	−0.2	*a* ^6^D_0½_— $51254_{1½}^{{}^{\circ}}$	
1953.00		3	51203.3	0.0	*a* ^6^D_2½_— $54375_{2½}^{{}^{\circ}}$	
1957.90		2	51075.1	+0.2	*a* ^6^D_1½_— $52593_{0½}^{{}^{\circ}}$	
1959.54		5	51032.3	+0.2	*a* ^4^F_2½_— $62333_{2½}^{{}^{\circ}}$	
1961.42		6	50983.5	−0.1	*a* ^4^P_0½_— $59816_{1½}^{{}^{\circ}}$	
1962.14		40	50964.7	0.0	*a* ^6^D_2½_— $54137_{1½}^{{}^{\circ}}$	
1962.92		20	50944.4	−0.3	*a* ^4^F_3½_— $64356_{3½}^{{}^{\circ}}$	
1963.02		2	50941.9	−0.4	*a* ^6^D_4½_— $57089_{4½}^{{}^{\circ}}$	
1967.09		2	50836.5	+0.2	*a* ^6^D_1½_— $52355_{0½}^{{}^{\circ}}$	
1967.41		10	50828.2	+0.6	*a* ^4^F_4½_— $65684_{5½}^{{}^{\circ}}$	
1968.67		8	50795.6	+0.1	*a* ^4^F_3½_— $64207_{4½}^{{}^{\circ}}$	
1973.32		20	50676.0	0.0	*a* ^6^D_3½_— $55392_{4½}^{{}^{\circ}}$	
1974.66		4	50641.6	−0.3	*a* ^4^G_5½_— $68078_{6½}^{{}^{\circ}}$	
1975.48		15	50620.7	−0.7	*a* ^6^D_4½_— $56768_{3½}^{{}^{\circ}}$	
1976.70		25	50589.4	+2.2	*a* ^6^S_2½_— $58007_{1½}^{{}^{\circ}}$	
1977.24		12	50575.6	+0.1	*a* ^4^G_5½_— $68012_{5½}^{{}^{\circ}}$	
1977.53		15	50568.1	−0.1	*a* ^6^D_1½_— $52087_{2½}^{{}^{\circ}}$	
1981.42		20	50468.8	+3.2	*a* ^6^D_4½_— $56612_{3½}^{{}^{\circ}}$	
1982.35		15	50445.1	−0.9	*a* ^6^D_3½_— $55162_{2½}^{{}^{\circ}}$	
1982.73		1	50435.5	−0.8	*a* ^6^S_2½_— $57856_{2½}^{{}^{\circ}}$	
1982.92		20	50430.7	−0.3	*a* ^6^D_0½_— $50430_{1½}^{{}^{\circ}}$	
1987.80		25	50301.9	−4.6	*a* ^6^D_3½_— $55022_{3½}^{{}^{\circ}}$	
1989.41		30	50266.1	−0.4	*a* ^6^D_4½_— $56413_{4½}^{{}^{\circ}}$	
1990.07		15	50249.4	$\left\{ \begin{array}{l} -0.1\\ -1.1 \end{array}\right.$	*a* ^4^F_2½_— $61550_{3½}^{{}^{\circ}}$ *a* ^6^D_2½_— $53422_{1½}^{{}^{\circ}}$	
1990.86		12	50229.6	+0.3	*a* ^6^D_4½_— $56376_{5½}^{{}^{\circ}}$	
1993.15		6	50171.8	−1.9	*a* ^4^D_2½_— $65141_{2½}^{{}^{\circ}}$	
1993.53		1	50162.3	−3.3	*a* ^6^D_2½_— $53338_{3½}^{{}^{\circ}}$	
1993.74		3	50156.9	−0.3	*a* ^6^D_2½_— $53329_{1½}^{{}^{\circ}}$	
1994.21		15	50145.1	−0.9	*a* ^4^F_4½_— $65003_{4½}^{{}^{\circ}}$	
1995.545		25	50111.6	−0.3	*a* ^4^F_4½_— $64969_{5½}^{{}^{\circ}}$	
1997.46		6	50063.7	−0.3	*a* ^4^P_1½_— $60656_{2½}^{{}^{\circ}}$	
1998.54		3	50036.6	−0.1	*a* ^4^F_1½_— $58747_{1½}^{{}^{\circ}}$	
1998.99		3	50025.2	0.0	*a* ^6^F_2½_— $61326_{3½}^{{}^{\circ}}$	
Air						
1999.83		12	49988.1	−0.2	*a* ^6^D_3½_— $54704_{2½}^{{}^{\circ}}$	
2001.70	3	30	49941.4	+0.4	*a* ^6^D_2½_— $53113_{2½}^{{}^{\circ}}$	
2002.59		2	49919.2	0.0	*a* ^6^D_1½_— $51438_{2½}^{{}^{\circ}}$	
2002.73	1	3	49915.7	+0.4	*a* ^4^P_0½_— $58747_{1½}^{{}^{\circ}}$	
2005.60		1	49844.3	+1.0	*a* ^4^D_3½_— $64990_{2½}^{{}^{\circ}}$	
2006.07		3	49832.6	+0.4	*a* ^4^P_2½_— $63266_{3½}^{{}^{\circ}}$	
2008.08	4	40	49782.7	+0.5	*a* ^6^D_3½_— $54498_{3½}^{{}^{\circ}}$	
2009.37		2	49750.8	+1.6	*a* ^4^D_3½_— $64896_{3½}^{{}^{\circ}}$	
2009.98	1	12	49735.7	+0.1	*a* ^6^D_1½_— $51254_{1½}^{{}^{\circ}}$	
2010.24	3	15	49729.3	+0.1	*a* ^6^D_2½_— $52901_{3½}^{{}^{\circ}}$	
2010.66	2	1	49718.9	+1.1	*a* ^4^G_4½_— $66270_{4½}^{{}^{\circ}}$	
2012.18		5	49681.3	0.0	*a* ^4^G_3½_— $66270_{4½}^{{}^{\circ}}$	i
2013.06	4	4	49659.6	+0.1	*a* ^6^D_3½_— $54375_{2½}^{{}^{\circ}}$	
2014.23	3	15	49630.7	+0.2	*a* ^6^D_2½_— $52803_{1½}^{{}^{\circ}}$	
2014.43	3	10	49625.8	+0.1	*a* ^4^F_1½_— $58336_{2½}^{{}^{\circ}}$	
2015.44	2	8	49601.0	+1.1	*a* ^4^F_2½_— $60900_{2½}^{{}^{\circ}}$	
2017.26	–	2	49556.2	+0.7	*a* ^4^P_2½_— $62989_{2½}^{{}^{\circ}}$	
2019.08	–	1	49511.6	−0.2	*a* ^6^S_2½_— $56932_{1½}^{{}^{\circ}}$	
2019.55	2	5	49500.0	+0.5	*a* ^4^F_4½_— $64356_{3½}^{{}^{\circ}}$	
2021.43		3	49454.0	−0.6	*a* ^6^S_2½_— $56874_{2½}^{{}^{\circ}}$	
2023.25		1	49409.5	+0.1	*a* ^4^G_2½_— $65644_{3½}^{{}^{\circ}}$	
2023.63		3	49400.3	+0.6	*a* ^4^P_1½_— $59992_{2½}^{{}^{\circ}}$	
2023.79		3	49396.4	+0.4	*a* ^4^D_1½_— $64030_{2½}^{{}^{\circ}}$	
2025.45	1	4	49355.9	+0.5	*a* ^4^F_2½_— $60656_{2½}^{{}^{\circ}}$	
2025.62		2	49351.7	+1.4	*a* ^4^F_4½_— $64207_{4½}^{{}^{\circ}}$	
2026.07	8	30	49340.8	+0.6	*a* ^6^D_3½_— $54056_{4½}^{{}^{\circ}}$	
2027.30	1	12	49310.8	+0.9	*a* ^6^D_3½_— $54026_{2½}^{{}^{\circ}}$	
2027.55	2	3	49304.8	+0.8	*a* ^4^F_3½_— $62715_{4½}^{{}^{\circ}}$	
2027.87	2	5	49296.9	+0.6	*a* ^4^F_1½_— $58007_{1½}^{{}^{\circ}}$	
2029.12	1	15	49266.6	+0.1	*a* ^4^G_5½_— $66703_{5½}^{{}^{\circ}}$	
2029.99	5	50	49245.5	+0.3	*a* ^6^D_4½_— $55392_{4½}^{{}^{\circ}}$	
2030.87		3	49224.2	+0.4	*a* ^4^P_1½_— $59816_{1½}^{{}^{\circ}}$	
2031.44		12	49210.4	+0.7	*a* ^4^D_3½_— $64356_{3½}^{{}^{\circ}}$	
2032.16		3	49192.9	+0.6	*a* ^6^S_2½_— $56612_{3½}^{{}^{\circ}}$	
2032.92		12	49174.5	−0.4	*a* ^4^P_0½_— $58007_{1½}^{{}^{\circ}}$	
2033.38		10	49163.4	+0.4	*a* ^4^D_3½_— $64310_{2½}^{{}^{\circ}}$	
2033.73		8	49154.9	+0.4	*a* ^6^D_0½_— $49154_{1½}^{{}^{\circ}}$	
2034.19		3	49143.8	−1.6	*a* ^6^F_1½_— $57856_{2½}^{{}^{\circ}}$	
2035.02	5	25	49123.8	−0.2	*a* ^6^S_2½_— $56544_{2½}^{{}^{\circ}}$	
2035.87	5	40	49103.3	+0.5	*a* ^6^D_2½_— $52275_{3½}^{{}^{\circ}}$	
2036.40		2	49090.5	−0.4	*a* ^4^G_4½_— $65644_{3½}^{{}^{\circ}}$	
2037.58	3	40	49062.1	−0.4	*a* ^4^D_2½_— $64030_{2½}^{{}^{\circ}}$	
2037.89	2	15	49054.6	+0.3	*a* ^4^G_3½_— $65644_{3½}^{{}^{\circ}}$	
2039.10	–	4	49025.5	+0.4	*a* ^4^F_3½_— $62437_{4½}^{{}^{\circ}}$	
2040.86	4	20	48983.3	+0.4	*a* ^6^D_0½_— $48982_{1½}^{{}^{\circ}}$	
2042.00	1	6	48956.0	+0.6	*a* ^4^F_2½_— $60256_{3½}^{{}^{\circ}}$	
2042.55		5	48942.7	+0.9	19070_4½_— $68012_{5½}^{{}^{\circ}}$	
2043.02	2	8	48931.3	+0.3	*a* ^4^F_4½_— $63788_{3½}^{{}^{\circ}}$	
2043.52	5	30	48919.6	−1.6	*a* ^4^F_3½_— $62333_{2½}^{{}^{\circ}}$	
2043.72		15	48914.7	+0.2	*a* ^6^D_2½_— $52087_{2½}^{{}^{\circ}}$	
2043.82		3	48912.3	+0.1	*a* ^6^D_1½_— $50430_{1½}^{{}^{\circ}}$	
2044.33	2	10	48900.1	+1.0	*a* ^4^P_2½_— $62333_{2½}^{{}^{\circ}}$	
2045.05	3	3	48883.0	−0.3	*a* ^4^D_3½_— $64030_{2½}^{{}^{\circ}}$	
2045.32	2	15	48876.4	+0.7	*a* ^6^D_4½_— $55022_{3½}^{{}^{\circ}}$	
2047.07	2	20	48834.8	+0.9	*a* ^4^G_5½_— $66270_{4½}^{{}^{\circ}}$	
2047.76		2	48818.2	−2.2	*a* ^4^D_2½_— $63788_{3½}^{{}^{\circ}}$	
2048.03	5	30	48811.8	+0.4	*a* ^6^D_4½_— $54958_{5½}^{{}^{\circ}}$	
2049.62	8	25	48773.9	+0.3	*a* ^6^D_1½_— $50292_{2½}^{{}^{\circ}}$	
2050.36		12	48756.3	+0.8	*a* ^4^G_2½_— $64990_{2½}^{{}^{\circ}}$	
2051.32	1?	1	48733.5	+0.4	*a* ^4^D_3½_— $63880_{2½}^{{}^{\circ}}$	
2053.10	5	15	48691.3	$\left\{ \begin{array}{l} +0.2\\ +0.8 \end{array}\right.$	*a* ^4^F_2½_— $59992_{2½}^{{}^{\circ}}$ *a* ^6^D_2½_— $51863_{3½}^{{}^{\circ}}$	
2053.35		3	48661.6	+0.2	*a* ^6^G_2½_— $64896_{3½}^{{}^{\circ}}$	
2054.67	10	50	48654.0	+0.4	*a* ^6^D_3½_— $53369_{4½}^{{}^{\circ}}$	
2055.19	2	3	48641.8	+0.6	*a* ^4^D_3½_— $63788_{3½}^{{}^{\circ}}$	
2055.54	1?	3	48633.5	+0.9	*a* ^4^F_2½_— $59933_{3½}^{{}^{\circ}}$	
2055.99	2	10	48622.8	+1.0	*a* ^6^D_3½_— $53338_{3½}^{{}^{\circ}}$	
2058.30	12	15	48568.3	+0.2	*a* ^4^F_2½_— $59869_{3½}^{{}^{\circ}}$	
2059.03	1	8	48550.9	−1.0	*a* ^4^G_3½_— $65141_{2½}^{{}^{\circ}}$	
2059.45	4	8	48541.1	+0.4	*a* ^4^F_1½_— $57252_{2½}^{{}^{\circ}}$	
2060.53	3	2	48515.7	+0.5	*a* ^4^F_2½_— $59816_{1½}^{{}^{\circ}}$	
2063.34		1	48449.7	−0.4	*a* ^4^G_4½_— $65003_{4½}^{{}^{\circ}}$	
2064.76	2	6	48416.3	+0.3	*a* ^4^G_4½_— $64969_{5½}^{{}^{\circ}}$	
2065.06	6*A*	3	48409.4	+0.3	*a* ^4^F_4½_— $63266_{3½}^{{}^{\circ}}$	
2065.57	3	30	48397.3	+0.1	*a* ^6^D_3½_— $53113_{2½}^{{}^{\circ}}$	
2067.52	–?	30	48351.7	+0.3	*a* ^6^D_4½_— $54498_{3½}^{{}^{\circ}}$	
2067.87	–?	10	48343.5	+0.4	*a* ^4^G_4½_— $64896_{3½}^{{}^{\circ}}$	
2069.36		10	48308.8	+2.2	*a* ^4^G_3½_— $64896_{3½}^{{}^{\circ}}$	
2069.77		3	48299.1	+0.6	*a* ^4^D_2½_— $63266_{3½}^{{}^{\circ}}$	
2071.19	2	40	48265.9	+0.4	*a* ^6^D_2½_— $51438_{2½}^{{}^{\circ}}$	
2071.95		3	48248.3	+0.5	*a* ^4^G_5½_— $65684_{5½}^{{}^{\circ}}$	
2074.63		15	48185.9	+0.5	*a* ^6^D_3½_— $52901_{3½}^{{}^{\circ}}$	
2075.59	2	30	48163.8	+0.1	*a* ^4^F_1½_— $56874_{2½}^{{}^{\circ}}$	
2075.93		10	48155.9	+0.5	*a* ^4^P_1½_— $58747_{1½}^{{}^{\circ}}$	
2076.92		2	48132.9	+0.3	*a* ^4^P_2½_— $61566_{2½}^{{}^{\circ}}$	
2077.36		5	48122.6	+0.7	*a* ^4^G_2½_— $64356_{3½}^{{}^{\circ}}$	
2077.61		6	48116.9	+0.4	*a* ^4^P_2½_— $61550_{3½}^{{}^{\circ}}$	
2077.92	1	10	48109.7	+0.4	*a* ^4^F_4½_— $62966_{5½}^{{}^{\circ}}$	
2078.32	1	40	48100.3	+0.7	*a* ^4^P_0½_— $56932_{1½}^{{}^{\circ}}$	
2079.11	20	80	48082.2	$\left\{ \begin{array}{l} +0.3\\ +0.3 \end{array}\right.$	*a* ^6^D_2½_— $51254_{1½}^{{}^{\circ}}$ *a* ^6^D_4½_— $54229_{5½}^{{}^{\circ}}$	
2081.75		3	48021.2	−0.6	*a* ^4^D_2½_— $62989_{2½}^{{}^{\circ}}$	
2083.70	1	10	47976.4	+0.7	*a* ^4^F_2½_— $59276_{3½}^{{}^{\circ}}$	
2084.23		12	47964.1	+0.9	*a* ^4^G_4½_— $64516_{4½}^{{}^{\circ}}$	
2084.88		5	47949.1	+0.5	*a* ^4^F_3½_— $61360_{4½}^{{}^{\circ}}$	
2086.37	2	3	47914.9	+0.6	*a* ^4^F_3½_— $61326_{3½}^{{}^{\circ}}$	
2086.58	5	4	47910.0	+0.6	*a* ^6^D_4½_— $54056_{4½}^{{}^{\circ}}$	
2087.34	2	2	47892.6	+0.4	*a* ^4^P_2½_— $61326_{3½}^{{}^{\circ}}$	
2087.46	5	4	47889.9	+0.5	*a* ^4^G_5½_— $65326_{5½}^{{}^{\circ}}$	
2088.18	60	30	47873.4	+0.7	*a* ^4^D_2½_— $51045_{3½}^{{}^{\circ}}$	
2089.13	40	20	47851.6	+0.8	*a* ^6^D_3½_— $52567_{4½}^{{}^{\circ}}$	
2089.49		4	47843.4	+0.8	*a* ^4^D_3½_— $62989_{2½}^{{}^{\circ}}$	
2091.23	2	4	47803.5	−0.1	*a* ^4^G_4½_— $64356_{3½}^{{}^{\circ}}$	
2093.79	20	20	47745.1	+0.6	*a* ^4^P_1½_— $58336_{2½}^{{}^{\circ}}$	
2094.72	50	25	47723.9	+0.6	*a* ^6^D_1½_— $49242_{2½}^{{}^{\circ}}$	
2095.78		1	47699.8	+1.0	*a* ^4^D_1½_— $62333_{2½}^{{}^{\circ}}$	
2097.76	2	3	47654.6	+0.2	*a* ^4^G_4½_— $64207_{4½}^{{}^{\circ}}$	
2098.22	60*A*	30	47644.2	+0.4	*a* ^4^F_3½_— $61055_{4½}^{{}^{\circ}}$	
2098.58	70	40	47636.2	+0.5	*a* ^6^D_1½_— $49154_{0½}^{{}^{\circ}}$	
2098.69	3	–	47633.7	+0.9	19070_4½_— $66703_{5½}^{{}^{\circ}}$	
2099.37	2	1	47618.3	+0.5	*a* ^4^G_3½_— $64207_{4½}^{{}^{\circ}}$	
2100.06	–	1	47602.6	+0.2	*a* ^6^S_2½_— $55022_{3½}^{{}^{\circ}}$	
2100.66	50	20	47589.0	+0.4	*a* ^6^D_0½_— $47588_{1½}^{{}^{\circ}}$	
2101.05	–?	3	47580.1	+0.3	*a* ^4^F_4½_— $62437_{4½}^{{}^{\circ}}$	
2101.66		4	47566.4	+0.2	*a* ^4^G_5½_— $65003_{4½}^{{}^{\circ}}$	
2101.96	10	2	47559.6	+0.6	*a* ^6^D_3½_— $52275_{3½}^{{}^{\circ}}$	
2102.21	3	l	47553.9	+0.5	*a* ^4^G_2½_— $63788_{3½}^{{}^{\circ}}$	
2103.16	30	15	47532.5	+0.4	*a* ^4^G_5½_— $64969_{5½}^{{}^{\circ}}$	
2105.07		3	47489.3	+0.3	*a* ^4^F_3½_— $60900_{2½}^{{}^{\circ}}$	
2106.04	2		47467.4	+0.5	*a* ^4^P_2½_— $60900_{2½}^{{}^{\circ}}$	
2106.17	50	30	47464.5	+0.4	*a* ^4^D_1½_— $48982_{1½}^{{}^{\circ}}$	
2107.26		3	47440.0	−0.7	*a* ^4^G_3½_— $64030_{2½}^{{}^{\circ}}$	
2108.38	–?	25	47414.8	−0.3	*a* ^4^P_1½_— $58007_{1½}^{{}^{\circ}}$	
2108.65	–?	15	47408.6	+0.1	*a* ^4^F_2½_— $58709_{3½}^{{}^{\circ}}$	
2110.33	50	30	47371.0	+0.3	*a* ^6^D_3½_— $52087_{2½}^{{}^{\circ}}$	
2113.92	20	2	47290.5	$\left\{ \begin{array}{l} +0.1\\ +0.5 \end{array}\right.$	*a* ^4^G_3½_— $63880_{2½}^{{}^{\circ}}$ *a* ^4^D_3½_— $62437_{4½}^{{}^{\circ}}$	
2114.16		1	47285.1	+0.9	*a* ^6^S_2½_— $54704_{2½}^{{}^{\circ}}$	
2115.08	10	2	47264.5	+0.3	*a* ^4^P_1½_— $57856_{2½}^{{}^{\circ}}$	
2115.35	20		47258.6	+0.2	*a* ^6^D_2½_— $50430_{1½}^{{}^{\circ}}$	
2115.65	2		47251.8	+0.2	*a* ^4^P_0½_— $56084_{1½}^{{}^{\circ}}$	
2115.91		2	47246.1	+1.5	*a* ^4^F_3½_— $60656_{2½}^{{}^{\circ}}$	
2116.94	25	2	47223.1	+0.3	*a* ^6^D_4½_— $53369_{4½}^{{}^{\circ}}$	
2118.03	15	5	47198.8	+0.3	*a* ^4^G_3½_— $63788_{3½}^{{}^{\circ}}$	
2118.34		2	47191.8	+0.9	*a* ^6^D_4½_— $53338_{3½}^{{}^{\circ}}$	
2118.87	40	20	47180.1	+0.2	*a* ^6^D_0½_— $47179_{1½}^{{}^{\circ}}$	
2120.38	1	1	47146.4	−0.3	*a* ^6^D_3½_— $51863_{3½}^{{}^{\circ}}$	
2121.60	100	25	47119.3	−0.5	*a* ^6^D_2½_— $50292_{2½}^{{}^{\circ}}$	
2123.42	15	20	47079.0	−0.4	*a* ^4^G_5½_— $64516_{4½}^{{}^{\circ}}$	
2124.14		1	47063.0	+0.3	*a* ^4^F_3½_— $60474_{2½}^{{}^{\circ}}$	
2125.58	4	10	47031.1	−0.4	*a* ^4^G_2½_— $63266_{3½}^{{}^{\circ}}$	
2126.86	2	4	47002.8	+0.3	18000_3½_— $65003_{4½}^{{}^{\circ}}$	
2127.44	12	20	46990.0	$\left\{ \begin{array}{l} +0.4\\ \;0.0 \end{array}\right.$	18000_3½_— $64990_{2½}^{{}^{\circ}}$ *a* ^4^P_2½_— $60424_{3½}^{{}^{\circ}}$	
2129.00	10	2	46955.6	+0.2	*a* ^4^S_2½_— $54375_{2½}^{{}^{\circ}}$	
2130.05	20	2	46932.4	+0.1	*a* ^4^D_1½_— $61566_{2½}^{{}^{\circ}}$	
2131.73	4	3	46895.5	0.0	18000_3½_— $64896_{3½}^{{}^{\circ}}$	
2134.044	25	10	46844.61	+0.12	*a* ^4^F_3½_— $60256_{3½}^{{}^{\circ}}$	
2135.04	–	20	46822.8	+0.4	*a* ^4^P_2½_— $60256_{3½}^{{}^{\circ}}$	
2137.135	20*A*	10	46776.87	+0.12	*a* ^4^F_1½_— $55488_{1½}^{{}^{\circ}}$	
2137.636	25	6	46765.9	+0.2	*a* ^6^D_1½_— $48284_{2½}^{{}^{\circ}}$	
2138.133	40	30	46755.0	+0.4	*a* ^6^D_4½_— $52901_{3½}^{{}^{\circ}}$	
2139.16	30	12	46732.6	+0.4	*a* ^4^F_4½_— $61589_{5½}^{{}^{\circ}}$	
2139.646	12	6	46722.0	+0.3	*a* ^4^D_3½_— $51438_{2½}^{{}^{\circ}}$	
2139.86	1		46717.3	+0.5	*a* ^6^S_2½_— $54137_{1½}^{{}^{\circ}}$	
2140.03	5	6	46713.6	+0.4	*a* ^4^G_4½_— $63266_{3½}^{{}^{\circ}}$	
2140.94		2	46693.7	+0.3	*a* ^4^F_4½_— $61550_{3½}^{{}^{\circ}}$	
2142.49	15	4	46660.0	+0.5	*a* ^4^P_1½_— $57252_{2½}^{{}^{\circ}}$	
2142.736	3	1	46645.61	+0.26	*a* ^4^P_0½_— $55488_{1½}^{{}^{\circ}}$	
2143.257	15+	3	46643.27	+0.35	*a* ^4^D_0½_— $59816_{1½}^{{}^{\circ}}$	
2144.08	40	4	46625.4	+0.1	*a* ^6^D_0½_— $46625_{0½}^{{}^{\circ}}$	
2145.28	6		46599.3	+0.4	*a* ^4^D_2½_— $61566_{2½}^{{}^{\circ}}$	
2146.02	8	3	46583.1	+0.3	*a* ^4^D_2½_— $61550_{3½}^{{}^{\circ}}$	
2146.13	25	8	46580.8	+0.6	*a* ^4^F_3½_— $59992_{2½}^{{}^{\circ}}$	
2147.17	1		46558.3	+0.2	*a* ^4^P_2½_— $59992_{2½}^{{}^{\circ}}$	
2147.31	35*A*	3	46555.2	−0.4	*a* ^4^F_2½_— $57856_{2½}^{{}^{\circ}}$	
2148.84		8	46522.1	+0.4	*a* ^4^F_3½_— $59933_{3½}^{{}^{\circ}}$	
2149.136		12	46515.69	+0.02	18000_3½_— $64516_{4½}^{{}^{\circ}}$	
2149.69	3	3	46503.7	+0.4	*a* ^4^F_4½_— $61360_{4½}^{{}^{\circ}}$	
2151.28	–	3	46469.3	+0.2	*a* ^4^F_4½_— $61326_{3½}^{{}^{\circ}}$	
2151.83		4	46457.4	+0.2	*a* ^4^F_3½_— $59869_{3½}^{{}^{\circ}}$	
2152.126	20	25	46451.07	+0.03	*a* ^4^F_1½_— $55162_{2½}^{{}^{\circ}}$	
2152.85	5	–	46435.5	+0.5	*a* ^4^P_2½_— $59869_{3½}^{{}^{\circ}}$	
2153.15	8	1	46429.0	+0.2	*a* ^4^F_2½_— $57729_{3½}^{{}^{\circ}}$	
2153.550	40	20	46420.36	+0.37	*a* ^6^D_4½_— $52567_{4½}^{{}^{\circ}}$	
2153.870	4	20	46413.46	+0.10	*a* ^4^G_4½_— $62966_{5½}^{{}^{\circ}}$	
2154.31	4	3	46404.0	+0.4	*a* ^4^D_3½_— $61550_{3½}^{{}^{\circ}}$	
2155.25	3		46383.7	+0.1	*a* ^4^F_4½_— $61240_{5½}^{{}^{\circ}}$	
2155.31	4	2	46382.5	+0.3	*a* ^4^P_2½_— $59816_{1½}^{{}^{\circ}}$	
2156.00		4	46367.61	+0.13	19276_2½_— $65644_{3½}^{{}^{\circ}}$	
2156.42	4	20	46358.6	+0.1	*a* ^4^D_2½_— $61326_{3½}^{{}^{\circ}}$	
2157.798	4	20	46328.98	+0.05	*a* ^6^D_3½_— $51045_{3½}^{{}^{\circ}}$	
2159.96	4	8	46282.6	+0.1	*a* ^4^P_1½_— $56874_{2½}^{{}^{\circ}}$	
2160.69	2		46267.0	+0.4	*a* ^4^D_1½_— $60900_{2½}^{{}^{\circ}}$	
2161.83	2	3	46242.6	+0.3	19442_6½_— $65684_{5½}^{{}^{\circ}}$	
2162.36		5	46231.2	0.0	20039_3½_— $66270_{4½}^{{}^{\circ}}$	6
2163.49		3	46207.1	+0.3	18000_3½_— $64207_{4½}^{{}^{\circ}}$	
2163.880	6	30	46198.78	+0.20	*a* ^4^F_4½_— $61055_{4½}^{{}^{\circ}}$	
2164.800		5	46179.15	−0.12	*a* ^4^D_3½_— $61326_{3½}^{{}^{\circ}}$	
2165.264		6	46169.25	+0.10	20534_5½_— $66703_{5½}^{{}^{\circ}}$	
2165.56	3		46163.0	+0.2	*a* ^4^G_4½_— $62715_{4½}^{{}^{\circ}}$	
2166.316	40	80	46146.84	+0.11	*a* ^6^D_3½_— $50863_{4½}^{{}^{\circ}}$	
2167.185	10	20	46128.33	+0.21	*a* ^6^D_4½_— $52275_{3½}^{{}^{\circ}}$	
2167.276		1	46126.40	+0.09	*a* ^4^G_3½_— $62715_{4½}^{{}^{\circ}}$	
2168.590	4	10	46098.45	+0.09	*a* ^4^G_2½_— $62333_{2½}^{{}^{\circ}}$	
2169.936	15	40	46069.86	$\left\{ \begin{array}{l} \;0.00\\ +0.28 \end{array}\right.$	*a* ^6^D_1½_— $47588_{1½}^{{}^{\circ}}$ *a* ^6^D_2½_— $49242_{2½}^{{}^{\circ}}$	
2172.206		12	46021.72	−0.43	*a* ^4^D_1½_— $60656_{2½}^{{}^{\circ}}$	
2172.926		6	46006.47	−0.15	19637_2½_— $65644_{3½}^{{}^{\circ}}$	
2173.26	1	2	45999.40	+0.04	18990_1½_— $64990_{2½}^{{}^{\circ}}$	
2173.55	25	50	45993.27	−0.08	*a* ^4^F_1½_— $54704_{2½}^{{}^{\circ}}$	
2173.825	5	15	45987.45	+0.07	*a* ^4^F_3½_— $59399_{4½}^{{}^{\circ}}$	
2175.494	12	8	45952.17	$\left\{ \begin{array}{l} +0.17\\ +0.29 \end{array}\right.$	*a* ^6^D_2½_— $49124_{3½}^{{}^{\circ}}$ *a* ^4^P_1½_— $56544_{2½}^{{}^{\circ}}$	
2175.54		3	45951.1	+0.2	*a* ^4^F_2½_— $57252_{2½}^{{}^{\circ}}$	
2176.424	2	12	45932.54	+0.02	19070_4½_— $65003_{4½}^{{}^{\circ}}$	
2176.88	3	15	45922.9	−0.2	20780_4½_— $66703_{5½}^{{}^{\circ}}$	
2177.13	3	4	45917.65	+0.01	*a* ^6^S_2½_— $53338_{3½}^{{}^{\circ}}$	
2177.52	4	l	45909.42	+0.14	*a* ^6^S_2½_— $53329_{1½}^{{}^{\circ}}$	
2177.546		30}	45908.88	+0.10	*a* ^4^D_3½_— $61055_{4½}^{{}^{\circ}}$	
2178.04	2		45898.46	+0.04	19070_4½_— $64969_{5½}^{{}^{\circ}}$	
2178.72	10	50	45884.14	+0.24	*a* ^4^G_4½_— $62437_{4½}^{{}^{\circ}}$	
2178.92	10	30	45879.9	+0.5	18000_3½_— $63880_{2½}^{{}^{\circ}}$	
2179.634		8	45864.9	$\left\{ \begin{array}{l} +0.1\\ -0.1 \end{array}\right.$	*a* ^4^F_3½_— $59276_{2½}^{{}^{\circ}}$ 19276_2½_— $65141_{2½}^{{}^{\circ}}$	
2180.46	20	40	45847.53	+0.16	*a* ^4^G_3½_— $62437_{4½}^{{}^{\circ}}$	
2180.68		8	45842.9	+0.2	*a* ^4^P_2½_— $59276_{3½}^{{}^{\circ}}$	
2181.49	15*A*	3	45825.9	+0.4	19070_4½_— $64896_{3½}^{{}^{\circ}}$	
2182.225		40	45810.45	+0.11	*a* ^4^D_2½_— $48982_{1½}^{{}^{\circ}}$	
2183.32		40	45787.48	0.02	18000_3½_— $63788_{3½}^{{}^{\circ}}$	
2183.94	2		45774.48	+0.14	*a* ^4^F_1½_— $54485_{0½}^{{}^{\circ}}$	
2185.42	–	50	45743.49	−0.04	*a* ^4^G_3½_— $62333_{2½}^{{}^{\circ}}$	
2185.75	5	40	45736.58	−0.02	20534_5½_— $66270_{4½}^{{}^{\circ}}$	
2186.738	30	40	45715.92	+0.05	*a* ^6^D_4½_— $51868_{3½}^{{}^{\circ}}$	
2186.835	4	10	45713.89	+0.09	19276_2½_— $64990_{2½}^{{}^{\circ}}$	
2187.82	3	1	45693.3	+0.2	*a* ^6^S_2½_— $53113_{2½}^{{}^{\circ}}$	
2188.06		2	45688.3	−0.4	*a* ^4^D_2½_— $60656_{2½}^{{}^{\circ}}$	
2189.19	30*A*	30	45664.7	+0.1	*a* ^4^F_1½_— $54375_{2½}^{{}^{\circ}}$	
2189.364	40	40	45661.09	0.07	*a* ^6^D_1½_— $47179_{1½}^{{}^{\circ}}$	
2189.494	50	50	45658.38	+0.20	*a* ^6^D_2½_— $48830_{3½}^{{}^{\circ}}$	
2189.740	6	10	45653.25	+0.31	*a* ^4^P_6½_— $54485_{0½}^{{}^{\circ}}$	
2189.850	40	80	45650.96	+0.08	*a* ^4^G_5½_— $63087_{6½}^{{}^{\circ}}$	
2190.80		6	45631.2	0.0	*a* ^4^F_2½_— $56932_{1½}^{{}^{\circ}}$	
2191.34	4	12	45619.9	+0.2	19276_2½_— $64896_{3½}^{{}^{\circ}}$	
2192.094	4	6	45604.23	—0.03	20039_3½_— $65444_{3½}^{{}^{\circ}}$	
2193.440	30	40	45576.25	+0.24	*a* ^6^D_3½_— $50292_{2½}^{{}^{\circ}}$	
2193.542	25	40	45574.13	+0.22	*a* ^4^F_2½_— $56874_{2½}^{{}^{\circ}}$	
2193.88		1	45567.1	+0.2	*a* ^4^F_4½_— $60424_{3½}^{{}^{\circ}}$	
2194.515	70	50	45593.92	+0.22	*a* ^4^D_0½_— $45553_{1½}^{{}^{\circ}}$	
2195.680		2	45529.76	+0.28	*a* ^4^G_5½_— $62966_{5½}^{{}^{\circ}}$	
2195.816		25	45526.94	+0.38	19442_6½_— $64969_{5½}^{{}^{\circ}}$	
2196.654		1	45509.57	+0.08	*a* ^4^D_3½_— $60656_{2½}^{{}^{\circ}}$	
2196.780	12	10	45506.96	+0.11	*a* ^4^D_2½_— $60474_{2½}^{{}^{\circ}}$	
2196.914		3	45504.18	0.00	19637_2½_— $65141_{2½}^{{}^{\circ}}$	
2197.504	8	10	45491.97	+0.19	*a* ^4^P_1½_— $56084_{1½}^{{}^{\circ}}$	
2197.585	1		45490.3	−0.3	20780_4½_— $66270_{4½}^{{}^{\circ}}$	
2198.008		6	45481.54	+0.23	*a* ^6^S_2½_— $52901_{3½}^{{}^{\circ}}$	
2198.676	40	60	45467.72	+0.19	*a* ^4^F_2½_— $56768_{3½}^{{}^{\circ}}$	
2199.166	40	20	45457.59	+0.57	*a* ^6^D_0½_— $45457_{0½}^{{}^{\circ}}$	
2199.742	2	8	45445.69	0.00	19070_4½_— $64516_{4½}^{{}^{\circ}}$	
2200.696	2		45426.0	+0.1	*a* ^4^F_1½_— $54137_{1½}^{{}^{\circ}}$	
2200.907		12	45421.64	+0.15	*a* ^4^F_4½_— $60278_{4½}^{{}^{\circ}}$	
2201.984		12	45399.42	+0.19	*a* ^4^F_4½_— $60256_{3½}^{{}^{\circ}}$	
2203.798	10	25	45362.06	+0.44	*a* ^4^F_4½_— $60218_{5½}^{{}^{\circ}}$	
2203.986	20	70	45358.19	+0.35	*a* ^4^D_1½_— $59992_{2½}^{{}^{\circ}}$	
2204.482	100	150	45347.98	+0.14	*a* ^6^D_4½_— *z* ${\;}^{6}F_{5½}^{{}^{\circ}}$	
2205.460	6	8	45327.88	+0.23	*a* ^4^D_3½_— $60474_{2½}^{{}^{\circ}}$	
2205.886	1	3	45319.12	+0.08	18990_1½_— $64310_{2½}^{{}^{\circ}}$	
2206.060	15	15	45315.55	−0.21	*a* ^4^G_2½_— $61550_{3½}^{{}^{\circ}}$	
2206.134	8	12	45314.03	+0.19	*a* ^4^P_2½_— $58747_{1½}^{{}^{\circ}}$	
2206.237	2	–	45311.91	+0.25	*a* ^4^F_2½_— $56612_{3½}^{{}^{\circ}}$	
2206.588	40	80	45304.71	+0.17	*a* ^4^P_0½_— $54137_{1½}^{{}^{\circ}}$	
2206.922	20	30	45297.85	+0.25	*a* ^4^F_3½_— $58709_{3½}^{{}^{\circ}}$	
2207.435	–	5	45287.33	+1.31	19070_4½_— $64356_{3½}^{{}^{\circ}}$	
2207.914	15	20	45277.50	−0.04	*a* ^4^G_5½_— $62714_{6½}^{{}^{\circ}}$	
2207.986	30	40	45276.02	+0.10	*a* ^4^F_3½_— $58687_{4½}^{{}^{\circ}}$	
2208.813		40	45259.07	+0.23	19637_2½_— $64896_{3½}^{{}^{\circ}}$	
2209.564	12	6	45243.69	+0.37	*a* ^4^F_2½_— $56544_{2½}^{{}^{\circ}}$	
2216.023	60	40	45111.84	−0.12	*a* ^4^D_2½_— $48284_{2½}^{{}^{\circ}}$	res
2216.296	8	5	45106.28	−0.21	*a* ^4^D_1½_— $46625_{0½}^{{}^{\circ}}$	
2217.582	4	3	45080.13	−0.05	19276_2½_— $64356_{3½}^{{}^{\circ}}$	
2217.765	12	15	45076.41	−0.03	*a* ^4^F_4½_— $59933_{3½}^{{}^{\circ}}$	
2219.588	6	12	45039.39	+0.01	18990_1½_— $64030_{2½}^{{}^{\circ}}$	
2219.740	10	15	45036.31	−0.01	*a* ^4^G_4½_— $61589_{5½}^{{}^{\circ}}$	
2219.882	2	2	45033.42	−0.06	19276_2½_— $64310_{2½}^{{}^{\circ}}$	
2220.938	30	60	45012.01	+0.09	*a* ^4^F_4½_— $59869_{3½}^{{}^{\circ}}$	
2221.525	10	15	45000.12	+0.10	*a* ^4^G_5½_— $62437_{4½}^{{}^{\circ}}$	
2221.652	5	5	44997.55	+0.09	*a* ^4^G_4½_— $61550_{3½}^{{}^{\circ}}$	
2222.070	10	30	44989.08	+0.18	18000_3½_— $62989_{2½}^{{}^{\circ}}$	
2223.206	2	2	44966.10	+0.26	*a* ^4^D_2½_— $59933_{3½}^{{}^{\circ}}$	
2223.326	4	6	44963.67	+0.21	20039_3½_— $65003_{4½}^{{}^{\circ}}$	
2223.450	12	12	44961.17	+0.24	*a* ^4^G_3½_— $61550_{3½}^{{}^{\circ}}$	
2225.230	15*A*	5	44925.20	+0.18	*a* ^4^F_3½_— $58336_{2½}^{{}^{\circ}}$	
2225.882	60	80	44912.05	+0.42	*a* ^6^D_0½_— $44911_{3½}^{{}^{\circ}}$	res
2226.320	12	15	44903.21	+0.33	*a* ^4^P_2½_— $58336_{2½}^{{}^{\circ}}$	
2226.40	8	15	44901.6	+0.3	*a* ^4^D_2½_— $59869_{3½}^{{}^{\circ}}$	
2226.56	50	60	44898.4	+0.3	*a* ^6^D_4½_— $51045_{3½}^{{}^{\circ}}$	
2226.68	20	25	44895.95	+0.46	*a* ^4^P_1½_— $55488_{1½}^{{}^{\circ}}$	
2226.77	20	70	44894.14			
2228.29	1	3	44863.5	−0.1	20780_2½_— $65644_{3½}^{{}^{\circ}}$	
2228.70	5	2	44855.26	+0.41	*a* ^6^S_2½_— $52275_{3½}^{{}^{\circ}}$	
2228.88	12	80	44851.64			
2229.026	10	6	44848.70	+0.22	*a* ^4^D_2½_— $59816_{1½}^{{}^{\circ}}$	
2229.620	75	100	44836.76	+0.14	*a* ^6^D_1½_— $46355_{2½}^{{}^{\circ}}$	
2229.730	8	25	44834.54	+0.32	*a* ^4^D_0½_— $58007_{1½}^{{}^{\circ}}$	
2231.080	12	30	44807.42	+0.02	*a* ^4^G_4½_— $61360_{4½}^{{}^{\circ}}$	
2231.84	15	5	44792.16	+0.11	20534_5½_— $65326_{5½}^{{}^{\circ}}$	
2232.11	3	3	44786.74	+0.10	*a* ^4^D_3½_— $59933_{3½}^{{}^{\circ}}$	
2232.284	6	4	44783.25	+0.03	*a* ^4^F_2½_— $56084_{1½}^{{}^{\circ}}$	
2232.56	4	8	44777.72	+0.09	23234_4½_— $68012_{5½}^{{}^{\circ}}$	
2232.80	3	2	44772.90	−0.25	*a* ^4^G_4½_— $61326_{3½}^{{}^{\circ}}$	
2234.63	5?	12	44736.24	−0.38	*a* ^4^G_3½_— $61326_{3½}^{{}^{\circ}}$	
2235.00	4	2	44728.84	−0.07	*a* ^4^F_1½_— $53440_{0½}^{{}^{\circ}}$	
2235.48	2		44719.24	−0.08	19637_2½_— $64356_{3½}^{{}^{\circ}}$	
2235.64	20	30	44716.03	+0,14	*a* ^4^D_4½_— $50863_{4½}^{{}^{\circ}}$	
2235.856	8	8	44711.71	−0.01	*a* ^4^F_1½_— $53422_{1½}^{{}^{\circ}}$	
2237.06	15	100	44687.65	−0.02	*a* ^4^G_4½_— $61240_{5½}^{{}^{\circ}}$	
2237.84	10	4	44672.08	−0.54	19637_2½_— $64310_{2½}^{{}^{\circ}}$	
2238.10	2	1	44666.8	+0.2	*a* ^6^S_2½_— $52087_{2½}^{{}^{\circ}}$	
2240.53	2?		44618.4	0.0	*a* ^4^F_1½_— $53329_{1½}^{{}^{\circ}}$	
2241.080	30	50	44607.5	0.0	*a* ^4^P_0½_— $53440_{0½}^{{}^{\circ}}$	
2241.282	504	10	44603.48	−0.10	19276_2½_— $63880_{2½}^{{}^{\circ}}$	6
2242.71	7	7	44575.08	+0.12	*a* ^4^F_3½_— $57986_{4½}^{{}^{\circ}}$	
2242.965	10	12	44570.02	+0.24	*a* ^4^P_1½_— $55162_{2½}^{{}^{\circ}}$	
2244.15	10	25	44546.48	+0.46	20780_4½_— $65326_{5½}^{{}^{\circ}}$	
2244.75	2	3	44534.6	+0.2	20455_1½_— $64990_{2½}^{{}^{\circ}}$	
2245.19	25	40	44525.8	0.0	*a* ^6^D_3½_— $49242_{2½}^{{}^{\circ}}$	
2245.90	5	3	44511.8	+0.1	19276_2½_— $63788_{3½}^{{}^{\circ}}$	
2246.35	8	10	44502.86	+0.20	*a* ^4^G_4½_— $61055_{4½}^{{}^{\circ}}$	
2246.64	15	20	44497.12	+0.07	*a* ^4^P_0½_— $53329_{1½}^{{}^{\circ}}$	
2247.67	6	40	44476.73	+0.10	20039_3½_— $64516_{4½}^{{}^{\circ}}$	
2248.27	25	40	44464.86	+0.14	*a* ^6^D_3½_— $49181_{4½}^{{}^{\circ}}$	*d*
2248.75	60*A*	100	44455.37	+0.19	*a* ^6^D_0½_— $44455_{0½}^{{}^{\circ}}$	
2249.28	6	5	44444.9	+0.2	*a* ^4^F_3½_— $57856_{2½}^{{}^{\circ}}$	
2249.38	15	12	44442.9	4−0.3	*a* ^6^S_2½_— $51863_{3½}^{{}^{\circ}}$	
2249.702	5	5	44436.56	4−0.22	18000_3½_— $62437_{4½}^{{}^{\circ}}$	
2249.804	20*A*	?	44434.54	−0.21	20534_5½_— $64969_{5½}^{{}^{\circ}}$	
2250.45	1	8	44421.79	4−0.12	*a* ^4^G_2½_— $60656_{2½}^{{}^{\circ}}$	
2250.560	3	3	44419.62	+0.03	*a* ^4^F_4½_— $59276_{3½}^{{}^{\circ}}$	
2250.730	30	50	44416.26	4−0.14	*a* ^6^D_2½_— $47588_{1½}^{{}^{\circ}}$	res
2251.14	30	40	44408.17	−0.03	*a* ^6^D_3½_— $49124_{3½}^{{}^{\circ}}$	6
2251.434	18	25	44402.38	4−0.12	*a* ^4^F_1½_— $53113_{2½}^{{}^{\circ}}$	
2251.924	5	25	44392.71	−0.25	19637_2½_— $64030_{2½}^{{}^{\circ}}$	
2254.98	10	20	44332.56	+0.06	18000_3½_— $62333_{2½}^{{}^{\circ}}$	
2255.715	10*A*	10	44318.12	4−0.16	*a* ^4^F_3½_— $57729_{3½}^{{}^{\circ}}$	
2255.770	1	1	44317.04	+0.08	20039_3½_— $64356_{3½}^{{}^{\circ}}$	
2256.18	2	6	44308.98	−0.01	*a* ^4^D_2½_— $59276_{3½}^{{}^{\circ}}$	
2256.85	12	30	44295.8	0.0	*a* ^4^P_2½_— $57729_{3½}^{{}^{\circ}}$	
2258.12	40	30	44270.9	+0.6	20039_3½_— $64310_{2½}^{{}^{\circ}}$	
2259.07	10	25	44252.3	0.0	*a* ^4^D_3½_— $59399_{4½}^{{}^{\circ}}$	
2259.56	10?	15	44242.7	0.0	19637_2½_— $63880_{2½}^{{}^{\circ}}$	
2259.66	3	3	44240.8	0.0	*a* ^6^D_2½_— $47413_{2½}^{{}^{\circ}}$	
2259.72	6	20	44239.6	−0.2	*a* ^4^G_2½_— $60474_{2½}^{{}^{\circ}}$	
2260.58	4	6	44222.8	0.0	20780_4½_— $65003_{4½}^{{}^{\circ}}$	
2261.946	1	25	44196.04	+0.42	19070_3½_— $63266_{3½}^{{}^{\circ}}$	
2262.30	6	20	44189.13	−0.17	*a* ^4^G_2½_— $60424_{3½}^{{}^{\circ}}$	
2262.406	4	4	44187.06	+0.13	*a* ^4^F_2½_— $55488_{1½}^{{}^{\circ}}$	
2263.40	1	2?	44167.66	$\left\{ \begin{array}{l} +0.12\\ -0.10 \end{array}\right.$	*a* ^4^P_1½_— $54760_{0½}^{{}^{\circ}}$ 20039_3½_— $64207_{4½}^{{}^{\circ}}$	
2263.53	15	80	44165.12	−0.04	*a* ^4^G_5½_— $61602_{6½}^{{}^{\circ}}$	
2264.178	8	35	44152.48	+0.04	*a* ^4^G_5½_— $61589_{5½}^{{}^{\circ}}$	
2265.338	7	35	44129.87	+0.08	*a* ^4^D_3½_— $59276_{3½}^{{}^{\circ}}$	
2265.67	35	20	44123.4	−0.1	23955_5½_— $68078_{6½}^{{}^{\circ}}$	
2266.05	3	8?	44116.0	+0.2	20780_4½_— $64896_{3½}^{{}^{\circ}}$	
2266.12	15	80	44114.6	+0.2	*a* ^6^D_3½_— $48830_{3½}^{{}^{\circ}}$	
2266.25	15	80	44112.1	0.0	*a* ^4^P_1½_— $54704_{2½}^{{}^{\circ}}$	
2267.30	4	4	44091.69	+0.05	*a* ^4^F_1½_— $52803_{1½}^{{}^{\circ}}$	
2268.064	2	2	44076.84	−0.03	22194_3½_— $66270_{4½}^{{}^{\circ}}$	
2269.08	2	4	44057.1	0.0	23955_5½_— $68012_{5½}^{{}^{\circ}}$	
2270.232	25	125	44034.75	$\left\{ \begin{array}{l} -0.17\\ +0.23 \end{array}\right.$	*a* ^6^D_1½_— $45553_{1½}^{{}^{\circ}}$ *a* ^4^F_4½_— $58891_{5½}^{{}^{\circ}}$	
2270.905	6	6	44021.70	+0.1	*a* ^4^G_2½_— $60256_{3½}^{{}^{\circ}}$	
2271.105	10	12	44017.82	+0.22	*a* ^6^S_2½_— $51438_{2½}^{{}^{\circ}}$	
2271.64	2	–	44007.4	0.0	*a* ^6^D_2½_— $47179_{1½}^{{}^{\circ}}$	
2272.09	–	3	43998.74	+0.10	18990_1½_— $62989_{2½}^{{}^{\circ}}$	
2272.510	8	25	43990.61	+0.01	20039_3½_— $64030_{2½}^{{}^{\circ}}$	
2272.96	5?	15	43981.9	−0.1	20534_5½_— $64516_{4½}^{{}^{\circ}}$	
2273.55	6	10	43970.49	+0.15	*a* ^4^P_0½_— $52803_{1½}^{{}^{\circ}}$	
2275.22	5	?	43938.2	0.0	*a* ^6^D_1½_— $45457_{0½}^{{}^{\circ}}$	
2275.98	3	2	43923.5	0.0	*a* ^4^G_5½_— $61360_{4½}^{{}^{\circ}}$	
2277.416	3	4	43895.86	+0.04	19070_4½_— $62966_{5½}^{{}^{\circ}}$	
2277.583	60*A*	20	43892.64	−0.44	*a* ^4^P_1½_— $54485_{0½}^{{}^{\circ}}$	
2277.977	10	35	43885.05	+0.05	*a* ^4^G_3½_— $60474_{2½}^{{}^{\circ}}$	
2278.108	25	30	43882.52	+0.06	*a* ^4^F_1½_— $52593_{0½}^{{}^{\circ}}$	
2278.704	10	12	43871.05	+0.05	*a* ^4^G_4½_— $60424_{3½}^{{}^{\circ}}$	
2279.213	10	3	43861.25	+0.03	*a* ^4^F_2½_— $55162_{2½}^{{}^{\circ}}$	
2279.672	1–	–?	43852.42	+0.08	*a* ^4^F_4½_— $58709_{3½}^{{}^{\circ}}$	
2280.300	8	30	43840.34	−0.02	20039_3½_— $63880_{2½}^{{}^{\circ}}$	
2280.621	30	12	43834.18	+0.21	*a* ^4^S_2½_— $51254_{1½}^{{}^{\circ}}$	
2280.802	10?	10	43830.7	0.0	*a* ^4^F_4½_— $58687_{0½}^{{}^{\circ}}$	
2282.202	25	75	43803.81	+0.02	*a* ^4^G_5½_— $61240_{5½}^{{}^{\circ}}$	6
2283.266	25*A*	5	43783.40	+0.10	*a* ^4^P_1½_— $54375_{2½}^{{}^{\circ}}$	
2283.437	4	3	43780.12	0.00	*a* ^4^D_2½_— $58747_{1½}^{{}^{\circ}}$	
2284.436	20	18	43760.98	−0.08	*a* ^4^P_0½_— $52593_{0½}^{{}^{\circ}}$	6
2284.619	10	40	43757.47	+0.11	*a* ^4^G_1½_— $59992_{2½}^{{}^{\circ}}$	6
2285.09	–?	1	43748.45	−0.01	20039_3½_— $63788_{3½}^{{}^{\circ}}$	
2285.75	1	3	43735.82	−0.17	20780_4½_— $64516_{4½}^{{}^{\circ}}$	
2286.28	5?	8	43725.69	+0.12	*a* ^4^G_4½_— $60278_{4½}^{{}^{\circ}}$	
2286.476	6	4	43721.94	+0.16	*a* ^4^F_2½_— $55022_{3½}^{{}^{\circ}}$	
2286.94	1–	1	43713.07	−0.01	19276_2½_— $62989_{2½}^{{}^{\circ}}$	
2287.46	8	5	43703.13	−0.18	*a* ^4^G_4½_— $60256_{3½}^{{}^{\circ}}$	
2289.01	12*A*	8	43673.5	+0.3	20534_5½_— $64207_{4½}^{{}^{\circ}}$	
2289.41	5	7	43665.9	+0.2	*a* ^4^G_4½_— $60218_{5½}^{{}^{\circ}}$	
2290.48	2	2	43645.5	+0.1	19442_6½_— $63087_{6½}^{{}^{\circ}}$	
2290.564	15	20	43643.92	+0.07	*a* ^4^F_1½_— $52355_{0½}^{{}^{\circ}}$	
2291.376	4	7	43628.45	−0.47	19637_2½_— $63266_{3½}^{{}^{\circ}}$	
2291.56	7	8	43625.0	+0.2	*a* ^6^S_2½_— $51045_{3½}^{{}^{\circ}}$	
2294.20	–	3	43574.7	+0.3	20455_1½_— $64030_{2½}^{{}^{\circ}}$	
2294.55	10?	35	43568.1	−0.1	*a* ^6^D_3½_— $48984_{2½}^{{}^{\circ}}$	res
2294.84	10	20	43562.6	+0.1	*a* ^4^D_3½_— $58709_{3½}^{{}^{\circ}}$	
2295.52	3	3	43549.7	−0.2	18000_3½_— $61550_{3½}^{{}^{\circ}}$	
2295.78	15	20	43544.8	+0.1	*a* ^4^P_1½_— $54137_{1½}^{{}^{\circ}}$	6
2295.985	15	25	43540.88	+0.02	*a* ^4^D_3½_— $58687_{4½}^{{}^{\circ}}$	
2296.873	4	5	43524.05	+0.09	19442_6½_— $62966_{5½}^{{}^{\circ}}$	
2296.956	8	6	43522.47	+0.02	*a* ^4^P_0½_— $52355_{0½}^{{}^{\circ}}$	
2297.930	1	10	43504.03	0.00	22140_2½_— $65644_{3½}^{{}^{\circ}}$	
2298.23	8	20	43498.4	+0.2	*a* ^4^P_2½_— $56932_{1½}^{{}^{\circ}}$	
2299.807	6	18	43468.53	−0.10	23234_4½_— $66703_{5½}^{{}^{\circ}}$	
2300.078	1	5	43463.40	+0.37	*a* ^4^F_3½_— $56874_{2½}^{{}^{\circ}}$	
2301.284	3	5	43440.63	−0.26	*a* ^4^P_2½_— $56874_{2½}^{{}^{\circ}}$	
2301.642	20	30	43433.87	+0.15	*a* ^4^P_1½_— $54026_{2½}^{{}^{\circ}}$	res
2302.139	–	1	43424.49	+0.32	20455_1½_— $63880_{2½}^{{}^{\circ}}$	
2303.274	25	50	43403.10	+0.57	*a* ^4^G_3½_— $59992_{2½}^{{}^{\circ}}$	
2303.819	25	75	43392.83	−0.02	*a* ^4^D_1½_— $44911_{1½}^{{}^{\circ}}$	res
2304.474	–	2	43380.50	−0.02	*a* ^4^G_4½_— $59933_{3½}^{{}^{\circ}}$	
2305.219	4	8	43366.48	+0.12	19070_4½_— $62437_{4½}^{{}^{\circ}}$	
2305.553	–	1	43360.20	+0.36	18000_3½_— $61360_{4½}^{{}^{\circ}}$	
2305.705	–	1	43357.34	+0.69	*a* ^4^F_3½_— $56768_{3½}^{{}^{\circ}}$	
2305.972	1	1	43352.32	+0.10	19637_2½_— $62989_{2½}^{{}^{\circ}}$	
2306.419	2	3	43343.92	−0.07	*a* ^4^G_3½_— $59933_{3½}^{{}^{\circ}}$	
2306.511	–?	4	43342.19	−0.05	18990_1½_— $62333_{2½}^{{}^{\circ}}$	
2306.918	15	40	43334.55	+0.04	*a* ^4^P_2½_— $56768_{3½}^{{}^{\circ}}$	res
2307.392	2	3	43325.64	+0.05	18000_3½_— $61326_{3½}^{{}^{\circ}}$	
2307.930	5	10	43315.54	−0.46	*a* ^4^G_4½_— $59869_{3½}^{{}^{\circ}}$	
2309.850	8	25	43279.54	+0.07	*a* ^4^G_3½_— $59869_{3½}^{{}^{\circ}}$	
2310.244	4	12	43272.16	+0.14	19442_6½_— $62714_{6½}^{{}^{\circ}}$	
2312.800	–	4	43224.35	+0.20	23046_3½_— $66270_{4½}^{{}^{\circ}}$	
2312.907	6	20	43222.35	+0.01	*a* ^4^D_1½_— $57856_{2½}^{{}^{\circ}}$	
2314.084	–	6	43200.36	−0.42	*a* ^4^F_3½_— $56612_{3½}^{{}^{\circ}}$	
2314.637	10	25	43190.04	+0.08	*a* ^4^D_3½_— $58336_{2½}^{{}^{\circ}}$	
2315.022	20	50	43182.87	−0.01	*a* ^6^D_2½_— $46355_{2½}^{{}^{\circ}}$	
2315.246	3	4	43178.69	+0.05	*a* ^4^P_2½_— $56612_{3½}^{{}^{\circ}}$	
2317.722	4	6	43132.57	+0.13	*a* ^4^F_3½_— $56544_{2½}^{{}^{\circ}}$	
2317.88	6	10	43129.62	−0.08	*a* ^4^F_4½_— $57986_{4½}^{{}^{\circ}}$	
2318.916	3	6	43110.36	+0.06	*a* ^4^P_2½_— $56544_{2½}^{{}^{\circ}}$	
2320.832	6	5	43074.76	+0.02	*a* ^4^F_2½_— $54375_{2½}^{{}^{\circ}}$	
2321.807	–	1	43056.68	0.00	19276_2½_— $62333_{2½}^{{}^{\circ}}$	
2322.594	2	3	43042.10	+0.13	*a* ^4^G_2½_— $59276_{3½}^{{}^{\circ}}$	
2322.720	5	8	43039.76	−0.02	*a* ^4^D_2½_— $58007_{1½}^{{}^{\circ}}$	
2323.038	15	25	43033.87	−0.01	*a* ^6^D_4½_— $49181_{4½}^{{}^{\circ}}$	6
2324.291	7	5	43010.67	+0.15	*a* ^6^S_2½_— $50430_{1½}^{{}^{\circ}}$	
2324.714	1	1	43002.84	−0.06	*a* ^6^D_2½_— $46175_{3½}^{{}^{\circ}}$	
2324.776	3	10	43001.70	$\left\{ \begin{array}{l} +0.02\\ +0.11 \end{array}\right.$	*a* ^4^F_3½_— $56413_{4½}^{{}^{\circ}}$ 22139_2½_— $65141_{2½}^{{}^{\circ}}$	
2326.091	15	60	42977.38	+0.02	*a* ^6^D_4½_— $49124_{3½}^{{}^{\circ}}$	
2327.592	8*A*	5	42949.67	−0.19	20039_3½_— $62989_{2½}^{{}^{\circ}}$	
2327.737	–	1	42947.0	−0.5	22194_3½_— $65141_{2½}^{{}^{\circ}}$	
2328.314	20	35	42936.35	−0.05	*a* ^6^D_1½_— $44455_{0½}^{{}^{\circ}}$	res
2329.691	6	15	42910.98	+0.06	*a* ^4^D_0½_— $56084_{1½}^{{}^{\circ}}$	
2330.89	5	8	42888.91	+0.03	*a* ^4^D_2½_— $57856_{2½}^{{}^{\circ}}$	
2331.778	3	4	42872.57	−0.13	*a* ^4^F_4½_— $57729_{3½}^{{}^{\circ}}$	
2331.816	1	1	42871.87	−0.03	*a* ^4^S_2½_— $50292_{2½}^{{}^{\circ}}$	
2333.146	25*A*	12	42847.44	−0.21	*a* ^4^P_1½_— $53440_{0½}^{{}^{\circ}}$	
2333.462	–	1	42841.64	−0.05	*a* ^4^G_5½_— $60278_{4½}^{{}^{\circ}}$	
2333.57	3	8	42839.66	+0.24	*a* ^4^D_3½_— $57986_{4½}^{{}^{\circ}}$	
2333.770	15	35	42835.98	−0.06	*a* ^6^D_1½_— $44354_{2½}^{{}^{\circ}}$	res
2334.070	5	5	42830.48	+0.02	*a* ^4^P_1½_— $53422_{1½}^{{}^{\circ}}$	
2335.205	6	25	42809.66	−0.01	*a* ^4^G_3½_— $59399_{4½}^{{}^{\circ}}$	
2335.548		5	42803.38	+0.16	25209_4½_— $68012_{5½}^{{}^{\circ}}$	
2335.933	2	8	42796.32	+0.08	22194_3½_— $64990_{2½}^{{}^{\circ}}$	
2336.716	6	15	42781.98	+0.16	*a* ^4^G_5½_— $60218_{5½}^{{}^{\circ}}$	
2337.799	6	20	42762.17	+0.07	*a* ^4^D_2½_— $57729_{3½}^{{}^{\circ}}$	
2338.125	–?	2	42756.21	−0.04	22139_2½_— $64896_{3½}^{{}^{\circ}}$	
2338.582		1	42747.86	−0.24	23955_5½_— $66703_{5½}^{{}^{\circ}}$	
2339.16	15	30	42737.29	+0.10	*a* ^4^P_1½_— $53329_{1½}^{{}^{\circ}}$	
2339.732	10	8	42726.84	+0.07	*a* ^4^F_1½_— $51438_{2½}^{{}^{\circ}}$	
2339.817	1	?	42725.29	+0.13	*a* ^4^F_2½_— $54026_{2½}^{{}^{\circ}}$	
2339.904	6	20	42723.70	+0.03	*a* ^4^G_4½_— $59276_{3½}^{{}^{\circ}}$	
2340.668	4	6	42709.76	+0.08	*a* ^4^D_3½_— $57856_{2½}^{{}^{\circ}}$	
2341.074	4	15	42702.35	+0.21	22194_3½_— $64896_{3½}^{{}^{\circ}}$	
2341.368	25	35	42696.99	−0.02	*a* ^6^D_3½_— $47413_{2½}^{{}^{\circ}}$	res
2341.442	1–	2?	42695.64	−0.18	19637_2½_— $62333_{2½}^{{}^{\circ}}$	
2341.903	1	6	42687.24	+0.10	*a* ^4^G_3½_— $59276_{3½}^{{}^{\circ}}$	
2346.864	1	5	42597.01	−0.15	23046_3½_— $65644_{3½}^{{}^{\circ}}$	
2347.644	1	3	42582.86	−0.04	*a* ^4^D_3½_— $57729_{3½}^{{}^{\circ}}$	
2348.015		7	42576.13	+0.39	18990_1½_— $61566_{2½}^{{}^{\circ}}$	
2349.262	3	20	42553.53	−0.02	20534_5½_— $63087_{6½}^{{}^{\circ}}$	
2349.839	6	10	42543.09	−0.05	*a* ^4^F_1½_— $51254_{1½}^{{}^{\circ}}$	6
2350.362	2	10	42533.62	−0.05	20455_1½_— $62989_{2½}^{{}^{\circ}}$	
2351.052	2	6	42521.14	+0.14	*a* ^4^P_1½_— $53113_{2½}^{{}^{\circ}}$	
2351.168	1–	1	42519.04	+0.26	19070_4½_— $61589_{5½}^{{}^{\circ}}$	
2351.496	4	12	42513.11	+0.01	*a* ^4^G_2½_— $58747_{2½}^{{}^{\circ}}$	
2352.924		4	42487.31	+0.07	22503_1½_— $64990_{2½}^{{}^{\circ}}$	
2353.334	2	8	42479.91	−0.01	19070_4½_— $61550_{3½}^{{}^{\circ}}$	
2353.667	1	5	42473.90	−0.07	18000_3½_— $60474_{2½}^{{}^{\circ}}$	
2354.018		2	42467.57	+0.46	23803_3½_— $66270_{4½}^{{}^{\circ}}$	
2355.019		4	42449.52	−0.41	23234_4½_— $65684_{5½}^{{}^{\circ}}$	
2356.469	1	2	42423.40	−0.04	18000_3½_— $60424_{3½}^{{}^{\circ}}$	
2357.268	1	5	42409.02	−0.11	23234_4½_— $65644_{3½}^{{}^{\circ}}$	
2357.931		1	42397.10	−0.20	20039_3½_— $62437_{4½}^{{}^{\circ}}$	
2358.816	12	18	42381.19	+0.01	*a* ^6^D_2½_— $45553_{1½}^{{}^{\circ}}$	res
2361.194	12*A*	10	42338.51	−0.09	*a* ^4^G_4½_— $58891_{5½}^{{}^{\circ}}$	
2362.108	3	15	42322.13	−0.16	22194_3½_— $64516_{4½}^{{}^{\circ}}$	
2362.484	1	10	42315.40	−0.15	23955_5½_— $66270_{4½}^{{}^{\circ}}$	
2362.528	1	4?	42314.61	−0.02	*a* ^4^D_0½_— $55488_{1½}^{{}^{\circ}}$	
2363.464	8	14	42297.85	−0.06	*a* ^4^D_1½_— $56932_{1½}^{{}^{\circ}}$	
2363.721		1	42293.25	−0.21	20039_3½_— $62333_{2½}^{{}^{\circ}}$	
2363.910	6	30	42289.87	+0.01	19070_4½_— $61360_{4½}^{{}^{\circ}}$	
2364.225	10	50	42284.24	+0.06	*a* ^4^D_2½_— $57252_{2½}^{{}^{\circ}}$	
2364.577	3	1	42277.94	−0.07	18000_3½_— $60278_{4½}^{{}^{\circ}}$	
2365.832		7	42255.52	−0.09	19070_4½_— $61326_{3½}^{{}^{\circ}}$	
2366.684	4	6	42240.31	−0.32	*a* ^4^D_1½_— $56874_{2½}^{{}^{\circ}}$	
2367.132	1	3	42232.32	+0.08	*a* ^4^F_4½_— $57089_{4½}^{{}^{\circ}}$	
2368.021		2	42216.46	−0.27	22139_2½_— $64356_{3½}^{{}^{\circ}}$	
2368.36	8	14	42210.42	−0.06	*a* ^4^P_1½_— $52803_{1½}^{{}^{\circ}}$	
2369.755	6	8	42185.57	0.00	*a* ^6^D_4½_— $48332_{5½}^{{}^{\circ}}$	
2370.056	10	45	42180.22	+0.01	20534_5½_— $62714_{6½}^{{}^{\circ}}$	
2370.624	5	12	42170.11	−0.02	19070_4½_— $61240_{5½}^{{}^{\circ}}$	
2371.058	1	7	42162.40	−0.22	22194_3½_— $64356_{3½}^{{}^{\circ}}$	
2371.218	2	5	42159.55	−0.09	19442_6½_— $61602_{6½}^{{}^{\circ}}$	
2371.936	5	20	42146.79	−0.13	19442_6½_— $61589_{5½}^{{}^{\circ}}$	
2372.610	5	30	42134.82	+0.08	*a* ^4^G_4½_— $58687_{4½}^{{}^{\circ}}$	
2373.305		1	42122.48	−0.58	*a* ^4^F_2½_— $53422_{1½}^{{}^{\circ}}$	
2373.462		4	42119.69	−0.20	*a* ^4^G_3½_— $58709_{3½}^{{}^{\circ}}$	
2373.679	1	3	42115.84	−0.08	22194_3½_— $64310_{2½}^{{}^{\circ}}$	
2374.454	5?	35	42102.10	−0.04	*a* ^4^G_2½_— $58336_{2½}^{{}^{\circ}}$	
2375.044	4	20	42091.64	+0.11	23234_4½_— $65326_{5½}^{{}^{\circ}}$	
2377.172	6	12	42053.96	+0.05	*a* ^4^P_2½_— $55488_{1½}^{{}^{\circ}}$	res
2377.393	6	8	42050.06	+0.29	19276_2½_— $61326_{3½}^{{}^{\circ}}$	
2378.130	8	15	42037.02	+0.03	*a* ^4^F_2½_— $53338_{3½}^{{}^{\circ}}$	
2378.603	10	15	42028.67	+0.04	*a* ^4^F_2½_— $53329_{1½}^{{}^{\circ}}$	
2379.466	1	2	42013.42	0.00	22194_3½_— $64207_{4½}^{{}^{\circ}}$	
2380.159	4	9	42001.19	−0.01	*a* ^4^P_1½_— $52593_{0½}^{{}^{\circ}}$	
2380.708	3	5	41991.51	+0.01	18000_3½_— $59992_{2½}^{{}^{\circ}}$	
2381.065	1	2	41985.22	+0.10	19070_4½_— $61055_{4½}^{{}^{\circ}}$	
2381.333	6	8	41980.47	+0.06	*a* ^4^F_3½_— $55392_{4½}^{{}^{\circ}}$	
2382.243	2	2	41964.45	0.00	*a* ^4^D_2½_— $56932_{1½}^{{}^{\circ}}$	
2382.364	7	20	41962.32	0.00	*a* ^4^G_5½_— $59399_{4½}^{{}^{\circ}}$	
2382.700		25	41956.40	0.00	23046_3½_— $65003_{4½}^{{}^{\circ}}$	
2383.500	2	3	41942.32	−0.12	*a* ^4^D_3½_— $57089_{4½}^{{}^{\circ}}$	
2383.884	1‒	1	41935.57	−0.03	20780_4½_— $62715_{4½}^{{}^{\circ}}$	
2384.033	3	6	41932.95	−0.01	18000_3½_— $59933_{3½}^{{}^{\circ}}$	
2385.253	7	15	41911.48	+0.09	*a* ^4^F_4½_— $56768_{3½}^{{}^{\circ}}$	
2385.335	5	7	41910.04	0.00	*a* ^4^D_1½_— $56544_{2½}^{{}^{\circ}}$	
2385.500	8	12	41907.14	−0.03	*a* ^4^D_2½_— $56874_{2½}^{{}^{\circ}}$	6
2386.447	2	9	41890.51	+0.14	22139_2½_— $64030_{2½}^{{}^{\circ}}$	6
2387.708	2	5	41868.39	−0.05	18000_3½_— $59869_{3½}^{{}^{\circ}}$	
2388.536	2	8	41853.88	+0.12	26158_4½_— $68012_{5½}^{{}^{\circ}}$	
2388.798	2	8	41849.28	−0.14	23046_3½_— $64896_{3½}^{{}^{\circ}}$	
2389.541		1	41836.28	+0.02	22194_3½_— $64030_{2½}^{{}^{\circ}}$	
2390.371	25	75	41821.75	+0.08	*a* ^6^S_2½_— $49242_{2½}^{{}^{\circ}}$	
2390.890	4	30	41812.68	+0.24	*a* ^4^F_2½_— $53113_{2½}^{{}^{\circ}}$	
2391.218	1	3	41806.94	0.00	22503_1½_— $64310_{2½}^{{}^{\circ}}$	
2391.574	2	3	41800.71	−0.08	*a* ^4^D_2½_— $56768_{3½}^{{}^{\circ}}$	
2391.717	4	8	41798.22	−0.05	19442_6½_— $61240_{5½}^{{}^{\circ}}$	
2392.932	20	60	41777.00	−0.11	*a* ^6^D_3½_— *z* ${\;}^{6}F_{4½}^{{}^{\circ}}$	res
2393.171	4	10	41772.83	+0.07	*a* ^4^G_2½_— $58007_{1½}^{{}^{\circ}}$	
2393.768	1	3	41762.41	−0.18	*a* ^4^P_1½_— $52855_{0½}^{{}^{\circ}}$	
2394.162	7	25	41755.54	+0.02	*a* ^4^F_4½_— $56612_{3½}^{{}^{\circ}}$	
2394.444	12	7	41750.62	+0.28	*a* ^4^F_3½_— $55162_{2½}^{{}^{\circ}}$	
2394.636	1	7	41747.27	−0.04	*a* ^4^G_3½_— $58336_{2½}^{{}^{\circ}}$	
2395.036	1	5	41740.30	+0.17	22139_2½_— $63880_{2½}^{{}^{\circ}}$	
2395.104	9	10	41739.12	+0.01	*a* ^6^D_2½_— $44911_{1½}^{{}^{\circ}}$	
2395.384	–	1	41734.24	+0.01	23234_4½_— $64969_{5½}^{{}^{\circ}}$	
2395.664	1	3	41729.36	−0.04	23955_5½_— $65684_{5½}^{{}^{\circ}}$	
2395.730	5	8	41728.21	$\left\{ \begin{array}{l} +0.01\\ +0.24 \end{array}\right.$	*a* ^4^P_2½_— $55162_{2½}^{{}^{\circ}}$ *a* ^4^D_3½_— $56874_{2½}^{{}^{\circ}}$	
2396.221	7	10	41719.66	−0.03	*a* ^4^F_1½_— $50430_{1½}^{{}^{\circ}}$	res
2397.097		200	41704.42	−0.24	*a* ^6^D_2½_— *z* ${\;}^{6}F_{2½}^{{}^{\circ}}$	res
2397.997		15	41688.77	−0.14	19637_2½_— $61326_{3½}^{{}^{\circ}}$	
2398.149		7	41686.12	+0.10	22194_3½_— $63880_{2½}^{{}^{\circ}}$	
2399.332	4	12	41665.57	+0.02	18990_1½_— $60656_{2½}^{{}^{\circ}}$	
2399.574	1	4	41661.37	+0.02	23234_4½_— $64896_{3½}^{{}^{\circ}}$	
2400.358	1	6	41647.76	−0.47	22139_2½_— $63788_{3½}^{{}^{\circ}}$	
2400.519	5	8	41644.97	+0.05	*a* ^4^D_2½_— $56612_{3½}^{{}^{\circ}}$	
2400.866	4	2	41638.95	−0.13	*a* ^6^D_3½_— $46355_{2½}^{{}^{\circ}}$	
2401.863	5	12	41621.67	+0.08	*a* ^4^D_3½_— $56768_{3½}^{{}^{\circ}}$	
2402.480	2	7	41610.98	+0.08	*a* ^4^F_3½_— $55022_{3½}^{{}^{\circ}}$	
2403.074	5	10	41600.69	+0.03	*a* ^4^F_2½_— $52901_{3½}^{{}^{\circ}}$	
2403.222	10	15	41598.13	−0.16	*a* ^4^P_0½_— $50430_{1½}^{{}^{\circ}}$	
2403.455	4	10	41594.10	−0.02	22194_3½_— $63788_{3½}^{{}^{\circ}}$	
2403.762	1	2	41588.79	+0.03	*a* ^4^P_2½_— $55022_{3½}^{{}^{\circ}}$	
2405.280		8	41562.54	+0.11	*a* ^6^S_2½_— $48982_{1½}^{{}^{\circ}}$	
2405.631	–?	1	41556.48	+0.06	*a* ^4^F_4½_— $56413_{4½}^{{}^{\circ}}$	
2406.576	1–	1	41540.16	+0.34	23450_2½_— $64990_{2½}^{{}^{\circ}}$	
2407.286	1	6	41527.92	+0.64	22503_1½_— $64030_{2½}^{{}^{\circ}}$	
2407.787	5	12	41519.27	+0.04	*a* ^4^F_4½_— $56376_{5½}^{{}^{\circ}}$	
2408.282	12*A*	15	41510.73	−0.13	20039_3½_— $61550_{3½}^{{}^{\circ}}$	
2409.226	5	20	41494.48	−0.02	*a* ^4^P_1½_— $52087_{2½}^{{}^{\circ}}$	res
2409.474	3	18	41490.21			
2409.827		4	41484.13	+0.42	18990_1½_— $60474_{2½}^{{}^{\circ}}$	
2410.694	–	2	41469.21	−0.36	23046_3½_— $64516_{4½}^{{}^{\circ}}$	
2410.854	1	1	41466.46	+0.18	24804_3½_— $66270_{4½}^{{}^{\circ}}$	
2411.287	7	3	41459.01	−0.09	*a* ^6^D_3½_— $46175_{3½}^{{}^{\circ}}$	
2411.538	6	20	41454.70	−0.02	*a* ^4^G_5½_— $58891_{5½}^{{}^{\circ}}$	
2411.820	10	25	41449.86	−0.08	*a* ^4^D_1½_— $56084_{1½}^{{}^{\circ}}$	
2412.064	2	2	41445.66	−0.06	23450_2½_— $64896_{3½}^{{}^{\circ}}$	
2412.76	5	8	41433.72	−0.06	*a* ^4^G_4½_— $57986_{4½}^{{}^{\circ}}$	
2414.118	4	7	41410.40	+0.13	*a* ^6^S_2½_— $48830_{3½}^{{}^{\circ}}$	
2414.806	6	25	41398.60	−0.04	18000_3½_— $59399_{4½}^{{}^{\circ}}$	4
2414.888	3	3	41397.20	−0.05	*a* ^4^G_3½_— $57986_{4½}^{{}^{\circ}}$	
2416.063	1	3	41377.06	+0.02	22503_1½_— $63880_{2½}^{{}^{\circ}}$	
2416.416		1	41371.02	+0.02	23955_5½_— $65326_{5½}^{{}^{\circ}}$	
2417.430	2	4	41353.67	+0.21	19070_4½_— $60424_{3½}^{{}^{\circ}}$	
2419.350	8	35	41320.85	+0.05	20039_3½_— $61360_{4½}^{{}^{\circ}}$	
2419.848	4	5	41312.34	+0.12	*a* ^4^D_0½_— $54485_{0½}^{{}^{\circ}}$	
2419.987	3	8	41309.98	+0.08	23046_3½_— $64356_{3½}^{{}^{\circ}}$	
2420.990	12	35	41292.86	+0.21	*a* ^4^F_3½_— $54704_{2½}^{{}^{\circ}}$	
2421.358	1	3	41286.59	+0.04	20039_3½_— $61326_{3½}^{{}^{\circ}}$	
2421.662	3	12	41281.40	−0.10	23234_4½_— $64516_{4½}^{{}^{\circ}}$	
2421.974	5	12	41276.09	−0.02	18000_3½_— $59276_{3½}^{{}^{\circ}}$	
2422.292	30*A*	20	41270.67	+0.16	*a* ^4^P_2½_— $54704_{2½}^{{}^{\circ}}$	
2422.529	8	25	41266.63	+0.01	*a* ^4^D_½_— $56413_{4½}^{{}^{\circ}}$	
2422.726	1	4	41263.28	+0.08	23046_3½_— $64310_{2½}^{{}^{\circ}}$	
2425.975	10*A*	6	41208.02	−0.01	19070_4½_— $60278_{4½}^{{}^{\circ}}$	
2426.482	1	4	41199.42	+0.06	23803_3½_— $65003_{4½}^{{}^{\circ}}$	
2426.558	4	10	41198.13	−0.02	19276_2½_— $60474_{2½}^{{}^{\circ}}$	6
2427.493	10	40	41182.26	−0.04	*a* ^6^D_2½_— $44354_{2½}^{{}^{\circ}}$	6 res
2427.807	4	6	41176.93	+0.15	*a* ^4^G_½_— $57729_{3½}^{{}^{\circ}}$	
2428.758	4	10	41160.80	+0.10	23046_3½_— $64207_{4½}^{{}^{\circ}}$	
2429.396	4	10	41149.99	+0.43	26929_5½_— $68078_{6½}^{{}^{\circ}}$	
2429.489	4	6	41148.42	+0.26	19070_½_— $60218_{5½}^{{}^{\circ}}$	
2429.528	3	8	41147.76	+0.14	19276_2½_— $60424_{3½}^{{}^{\circ}}$	
2429.971	1	6	41140.26	+0.01	*a* ^4^G_3½_— $57729_{3½}^{{}^{\circ}}$	6
2430.788	3	10	41126.44	+0.11	22139_2½_— $63266_{3½}^{{}^{\circ}}$	
2431.373	5	18	41116.54	+0.06	*a* ^4^D_2½_— $56084_{1½}^{{}^{\circ}}$	
2431.714	5	12	41110.78	+0.01	20455_1½_— $61566_{2½}^{{}^{\circ}}$	
2432.797		6	41092.47	+0.09	23803_3½_— $64896_{3½}^{{}^{\circ}}$	
2433.139	5	8	41086.70	+0.09	*a* ^4^F_3½_— $54498_{3½}^{{}^{\circ}}$	6 res
2433.982	25*A*	20	41072.47	+0.25	22194_3½_— $63266_{3½}^{{}^{\circ}}$	
2434.254	10*A*	12	41067.88	+0.05	20534_5½_— $61602_{6½}^{{}^{\circ}}$	
2434.453	5	8	41064.53	+0.06	*a* ^4^P_2½_— $54498_{3½}^{{}^{\circ}}$	
2435.008	10	50	41055.17	+0.06	20534_5½_— $61589_{5½}^{{}^{\circ}}$	
2435.445	4	20	41047.80	0.00	23955_5½_— $65003_{4½}^{{}^{\circ}}$	
2437.155	1‒	1	41019.00	−0.13	19637_2½_— $60656_{2½}^{{}^{\circ}}$	
2437.332	3	6	41016.02	−0.04	20039_3½_— $61055_{4½}^{{}^{\circ}}$	
2437.471	4	15	41013.68	−0.02	23955_5½_— $64969_{5½}^{{}^{\circ}}$	6
2439.473	4	12	40980.03	+0.10	19276_2½_— $60256_{3½}^{{}^{\circ}}$	
2439.808	2	7	40974.40	+0.20	*a* ^4^F_2½_— $52275_{3½}^{{}^{\circ}}$	
2439.913	2	7	40972.64	+0.01	23234_4½_— $64207_{4½}^{{}^{\circ}}$	
2440.432	10	20	40963.93	+0.11	*a* ^4^D_0½_— $54137_{1½}^{{}^{\circ}}$	
2441.612	7	12	40944.13			6
2441.790		7	40941.14	−0.58	*a* ^4^P_2½_— $54375_{2½}^{{}^{\circ}}$	
2443.858		2	40906.51	+0.31	23450_2½_— $64356_{3½}^{{}^{\circ}}$	
2446.394	25	120	40864.10	+0.05	*a* ^6^S_2½_— $48284_{2½}^{{}^{\circ}}$	6
2446.570	1	1	40861.16	−0.07	20039_3½_— $60900_{2½}^{{}^{\circ}}$	
2447.255	4	12	40849.73	+0.10	22139_2½_— $62989_{2½}^{{}^{\circ}}$	
2447.52		1	40845.3	−0.2	*a* ^4^P_1½_— $51438_{2½}^{{}^{\circ}}$	
2448.00	5	15	40837.3	0.0	19637_2½_— $60474_{2½}^{{}^{\circ}}$	
2448.237	4	50	40833.34	+0.04	23046_3½_— $63880_{2½}^{{}^{\circ}}$	
2448.662	4	8	40826.26	+0.07	20534_5½_— $61360_{4½}^{{}^{\circ}}$	
2448.719	3	4	40825.31	−0.03	18990_2½_— $59816_{1½}^{{}^{\circ}}$	
2449.694	4	20	40809.06	−0.02	20780_4½_— $61589_{5½}^{{}^{\circ}}$	
2450.324	2	7	40798.57	+0.11	19070_4½_— $59869_{3½}^{{}^{\circ}}$	
2451.032	3	7	40786.78	+0.02	19637_2½_— $60424_{3½}^{{}^{\circ}}$	
2451.468		50	40779.53	+0.11	*a* ^6^D_1½_— $42298_{1½}^{{}^{\circ}}$	
2451.660		1	40776.33	+0.03	19442_6½_— $60218_{5½}^{{}^{\circ}}$	
2453.760	3	10	40741.44	0.04	23046_3½_— $63788_{3½}^{{}^{\circ}}$	
2455.506	40*A*	10	40712.48	−0.05	23803_3½_— $64516_{4½}^{{}^{\circ}}$	
2455.722	2	5	40708.90	+0.04	18000_3½_— $58709_{3½}^{{}^{\circ}}$	
2455.866	6	35	40706.51	+0.05	20534_5½_— $61240_{5½}^{{}^{\circ}}$	6
2456.072	4	8	40703.09	−0.01	*a* ^4^P_2½_— $54137_{1½}^{{}^{\circ}}$	
2457.043	1	3	40687.01	−0.17	18000_3½_— $58687_{4½}^{{}^{\circ}}$	
2458.529	3	4?	40662.42	+0.09	*a* ^4^G_3½_— $57252_{2½}^{{}^{\circ}}$	
2458.564	5	30	40661.84	−0.04	*a* ^4^P_1½_— $51254_{1½}^{{}^{\circ}}$	
2458.838	3	20	40657.31	+0.17	19276_2½_— $59933_{3½}^{{}^{\circ}}$	
2459.602	20	10	40644.68	+0.10	*a* ^4^F_3½_— $54056_{4½}^{{}^{\circ}}$	
2459.876	4	30	40640.15	0.00	*a* ^4^G_2½_— $56874_{2½}^{{}^{\circ}}$	6
2461.148	5	20	40619.15	+0.08	19637_2½_— $60256_{3½}^{{}^{\circ}}$	
2461.294	3	8	40616.74	−0.03	20039_3½_— $60656_{2½}^{{}^{\circ}}$	
2461.442	5	15	40614.30	+0.02	*a* ^4^F_3½_— $54026_{2½}^{{}^{\circ}}$	
2464.616	15	40	40562.00	+0.05	*a* ^4^F_2½_— $51863_{3½}^{{}^{\circ}}$	
2464.70	1	4	40560.6	−0.4	23955_5½_— $64516_{4½}^{{}^{\circ}}$	
2465.148		15	40553.25	−0.08	23234_4½_— $63788_{3½}^{{}^{\circ}}$	
2465.598	2	7	40545.85	−0.06	20780_2½_— $61326_{3½}^{{}^{\circ}}$	
2465.667	2	20	40544.72	−0.04	26158_4½_— $66703_{5½}^{{}^{\circ}}$	7
2465.962	4	30	40539.87	+0.09	19276_2½_— $59816_{1½}^{{}^{\circ}}$	7
2466.176	2	5	40536.35	+0.03	*a* ^4^G_4½_— $57089_{4½}^{{}^{\circ}}$	
2466.330	5	10	40533.82	+0.05	*a* ^4^G_2½_— $56768_{3½}^{{}^{\circ}}$	
2466.522	35	80	40530.66	$\left\{ \begin{array}{l} -0.01\\ -0.18 \end{array}\right.$	*a* ^6^D_1½_— *z* ${\;}^{6}F_{2½}^{{}^{\circ}}$ *a* ^6^F_1½_— $49242_{2½}^{{}^{\circ}}$	res
2467.078	3	9	40521.53	+0.08	20534_5½_— $61055_{4½}^{{}^{\circ}}$	
2468.006	2	10	40506.29	+0.13	23803_3½_— $64310_{2½}^{{}^{\circ}}$	
2468.404	6	15	40499.76	−0.03	*a* ^4^G_3½_— $57089_{4½}^{{}^{\circ}}$	
2469.206	2	6	40486.61	+0.07	22503_1½_— $62989_{2½}^{{}^{\circ}}$	
2469.873	3	25	40475.67	+0.15	25209_4½_— $65684_{5½}^{{}^{\circ}}$	4
2470.804	8	70	40460.43	0.00	20780_4½_— $61240_{5½}^{{}^{\circ}}$	
2471.740	15	40	40445.11	+0.07	20455_1½_— $60900_{2½}^{{}^{\circ}}$	
2472.380	1	3	40434.64	−0.29	20039_2½_— $60474_{2½}^{{}^{\circ}}$	res
2473.752		1	40412.21	−0.01	19404_0½_— $59816_{1½}^{{}^{\circ}}$	
2474.278		2	40403.62	−0.04	23803_3½_— $64207_{4½}^{{}^{\circ}}$	
2475.474	1	3	40384.43	+0.03	20039_3½_— $60424_{3½}^{{}^{\circ}}$	
2475.588	3	50	40382.24			
2475.844	4	10	40378.07	+0.17	*a* ^4^G_2½_— $56612_{3½}^{{}^{\circ}}$	
2477.284	3	15	40354.60	−0.22	19637_2½_— $59992_{2½}^{{}^{\circ}}$	
2477.796	30	200	40346.26	−0.01	*a* ^4^D_4½_— *z* ${\;}^{6}F_{4½}^{{}^{\circ}}$	6
2478.313	2	20	40337.84	+0.14	23450_2½_— $63788_{3½}^{{}^{\circ}}$	
2478.402	2	6	40336.40	+0.12	18000_3½_— $58336_{2½}^{{}^{\circ}}$	
2478.874	4	10	40328.72	+0.06	19070_4½_— $59399_{4½}^{{}^{\circ}}$	
2480.041	3	4	40309.74	+0.18	*a* ^4^G_2½_— $56544_{2½}^{{}^{\circ}}$	
2480.867	3	6	40296.32	+0.04	19637_2½_— $59933_{3½}^{{}^{\circ}}$	
2481.546	10	30	40285.30	−0.02	*a* ^4^G_3½_— $56874_{2½}^{{}^{\circ}}$	
2482.154		3	40275.43	+0.01	20780_4½_— $61055_{4½}^{{}^{\circ}}$	
2482.390	3	2	40271.60	0.00	*a* ^4^F_1½_— $48982_{1½}^{{}^{\circ}}$	
2482.688	3	4	40266.77	−0.02	*a* ^4^D_0½_— $53440_{0½}^{{}^{\circ}}$	
2483.592	4	12	40252.11	+0.01	23955_5½_— $64207_{4½}^{{}^{\circ}}$	
2483.744	5	7	40249.64	+0.04	*a* ^4^D_0½_— $53422_{1½}^{{}^{\circ}}$	
2484.008	7	20	40245.37	+0.02	*a* ^4^D_3½_— $55392_{4½}^{{}^{\circ}}$	
2484.404	7	40	40238.95	−0.02	20039_3½_— $60278_{4½}^{{}^{\circ}}$	
2484.848	2	4	40231.76	0.00	19637_2½_— $59869_{3½}^{{}^{\circ}}$	
2485.160	1	2	40226.71	+0.21	23803_3½_— $64030_{2½}^{{}^{\circ}}$	
2485.606	1	3	40219.50	0.00	23046_3½_— $63266_{3½}^{{}^{\circ}}$	
2485.779	5	15	40216.70	−0.01	20039_3½_— $60256_{3½}^{{}^{\circ}}$	res
2486.429	6	18	40206.18	+0.05	19070_4½_— $59276_{3½}^{{}^{\circ}}$	
2486.776	3	6	40200.57	−0.01	20455_1½_— $60656_{2½}^{{}^{\circ}}$	
2487.155	2	1	40194.45	−0.03	*a* ^4^D_2½_— $55162_{2½}^{{}^{\circ}}$	
2487.231	3	5	40193.22	−0.01	22139_2½_— $62333_{2½}^{{}^{\circ}}$	
2488.120	8	30	40178.86	$\left\{ \begin{array}{l} -0.08\\ -0.06 \end{array}\right.$	*a* ^4^G_3½_— $56768_{3½}^{{}^{\circ}}$ 19637_2½_— $59816_{1½}^{{}^{\circ}}$	7
2488.780	30	120	40168.21	0.00	*a* ^6^S_2½_— $47588_{1½}^{{}^{\circ}}$	4
2488.932	15	7	40165.76	+0.12	*a* ^4^F_4½_— $55022_{3½}^{{}^{\circ}}$	
2489.231	40	200	40160.93	+0.07	*a* ^6^D_3½_— *z* ${\;}^{6}F_{3½}^{{}^{\circ}}$	res
2489.514	5	12	40156.37	+0.04	*a* ^4^D_0½_— $53329_{1½}^{{}^{\circ}}$	
2489.900	5	10	40150.14	−0.06	*a* ^4^P_0½_— $48982_{1½}^{{}^{\circ}}$	
2490.586	4	15	40139.08	−0.04	22194_3½_— $62333_{2½}^{{}^{\circ}}$	
2490.718	12	25	40136.95	0.00	*a* ^4^F_2½_— $51438_{2½}^{{}^{\circ}}$	
2492.252	4	14	40112.25	+0.04	26158_4½_— $66270_{4½}^{{}^{\circ}}$	
2492.928	8	80	40101.38	+0.02	*a* ^4^F_4½_— $54958_{5½}^{{}^{\circ}}$	
2493.54	3	7	40091.54	−0.01	24804_3½_— $64896_{3½}^{{}^{\circ}}$	
2494.738		2	40072.29	+0.07	24918_1½_— $64990_{2½}^{{}^{\circ}}$	
2494.872		7	40070.14	−0.11	*a* ^4^D_1½_— $54704_{2½}^{{}^{\circ}}$	
2495.522	5	10	40059.70	+0.10	*a* ^4^G_4½_— $56612_{3½}^{{}^{\circ}}$	
2496.648	50	120	40041.63	−0.15	*a* ^6^D_3½_— $44758_{4½}^{{}^{\circ}}$	5
2497.480	35	75	40028.29	+0.03	*a* ^6^D_4½_— $46175_{3½}^{{}^{\circ}}$	5
2498.076	2	3	40018.74	0.00	20455_1½_— $60474_{2½}^{{}^{\circ}}$	
2499.223	8	16	40000.38	+0.09	19276_2½_— $59276_{3½}^{{}^{\circ}}$	
2499.330	2	4	39998.67	−0.09	24991_1½_— $64990_{2½}^{{}^{\circ}}$	
2499.692	15	100	39992.88	−0.02	*a* ^6^S_2½_— $47413_{2½}^{{}^{\circ}}$	
2499.934	3	4	39989.00	+0.12	*a* ^4^P_2½_— $53422_{1½}^{{}^{\circ}}$	
2500.11	12	30	39986.19	−0.03	18000_3½_— $57986_{4½}^{{}^{\circ}}$	
2500.217	1	3	39984.48	+0.12	23803_3½_— $63788_{3½}^{{}^{\circ}}$	
2501.020	1	10	39971.64	−0.05	25169_1½_— $65141_{2½}^{{}^{\circ}}$	res
2501.877		10	39957.95	−0.06	*a* ^4^F_3½_— $53369_{4½}^{{}^{\circ}}$	
2502.072	2	10	39954.84	+0.11	*a* ^4^G_3½_— $56544_{3½}^{{}^{\circ}}$	
2502.162	3	4?	39953.40	+0.08	*a* ^4^F_2½_— $51254_{1½}^{{}^{\circ}}$	
2502.836	1	3	39942.64	−0.16	23046_3½_— $62989_{2½}^{{}^{\circ}}$	
2505.264		1	39903.93	−0.04	*a* ^4^P_2½_— $53338_{3½}^{{}^{\circ}}$	
2505.790	3	5	39895.56	−0.05	*a* ^4^P_2½_— $53329_{1½}^{{}^{\circ}}$	
2506.048	–?	80	39891.45	+0.15	28187_6½_— $68078_{6½}^{{}^{\circ}}$	6
2507.994	12′	30	39860.50	0.00	*a* ^4^G_4½_— $56413_{4½}^{{}^{\circ}}$	6
2508.274	2	3	39856.05	+0.05	18000_3½_— $57856_{2½}^{{}^{\circ}}$	
2508.582	1	3	39851.16	−0.08	*a* ^4^D_1½_— $54485_{0½}^{{}^{\circ}}$	
2508.690	–?	4	39849.44	−0.02	*a* ^4^G_2½_— $56084_{1½}^{{}^{\circ}}$	
2509.386	2	3	39838.39	−0.04	*a* ^4^P_1½_— $50430_{1½}^{{}^{\circ}}$	
2509.955	6	40	39829.35	−0.05	20039_3½_— $59869_{3½}^{{}^{\circ}}$	7
2510.246		3	39824.74	−0.16	28187_6½_— $68012_{5½}^{{}^{\circ}}$	
2510.348	–?	2	39823.12	−0.19	*a* ^4^G_4½_— $56376_{5½}^{{}^{\circ}}$	
2510.482	8	80	39821.00	−0.06	19070_4½_— $58891_{5½}^{{}^{\circ}}$	
2510.799		3	39815.97	+0.17	23450_2½_— $63266_{3½}^{{}^{\circ}}$	
2512.186	2	4	39793.99	+0.07	25209_4½_— $65003_{4½}^{{}^{\circ}}$	
2513.435	1	30	39774.22	4−0.06	26929_5½_— $66703_{5½}^{{}^{\circ}}$	6
2514.358	2	4	39759.62	+0.11	*a* ^6^S_2½_— $47179_{1½}^{{}^{\circ}}$	
2514.526	3	20	39756.96	−0.02	18990_1½_— $58747_{1½}^{{}^{\circ}}$	res
2515.324	3	30	39744.35	$\left\{ \begin{array}{l} +0.18\\ -0.01 \end{array}\right.$	*a* ^4^F_2½_— $51045_{3½}^{{}^{\circ}}$ 20534_5½_— $60278_{4½}^{{}^{\circ}}$	
2515.508	5	8	39741.44	−0.02	*a* ^4^D_1½_— $54375_{2½}^{{}^{\circ}}$	
2515.806	2	10	39736.73	−0.06	*a* ^4^D_2½_— $54704_{2½}^{{}^{\circ}}$	
2516.138		10	39731.49	−0.14	23234_4½_— $62966_{5½}^{{}^{\circ}}$	
2516.284	1	3	39729.18	−0.04	18000_3½_— $57729_{3½}^{{}^{\circ}}$	
2517.406	1	25	39711.48	−0.22	24804_3½_— $64516_{4½}^{{}^{\circ}}$	5
2518.144	6	50	39699.84	+0.03	*a* ^4^P_1½_— $50292_{2½}^{{}^{\circ}}$	
2518.973		4	39686.78	−0.16	25209_4½_— $64896_{3½}^{{}^{\circ}}$	
2519.126	4	7	39684.37	−0.12	20534_5½_— $60218_{5½}^{{}^{\circ}}$	
2519.444	8	30	39679.36	−0.06	*a* ^4^P_2½_— $53113_{2½}^{{}^{\circ}}$	res
2520.10	1	3	39669.0	−0.2	23046_3½_— $62715_{4½}^{{}^{\circ}}$	
2521.156	3	10	39652.42	−0.02	*a* ^4^G_5½_— $57089_{4½}^{{}^{\circ}}$	
2521.686	3*A*	3	39644.09	+0.33	20780_4½_— $60424_{3½}^{{}^{\circ}}$	
2521.853	3	3	39641.46	+0.11	*a* ^4^F_4½_— $54498_{3½}^{{}^{\circ}}$	
2522.039	30	60	39638.54	+0.04	*a* ^6^D_3½_— $44354_{2½}^{{}^{\circ}}$	
2522.270	3	8	39634.91	+0.21	28377_5½_— $68012_{5½}^{{}^{\circ}}$	
2526.208	4	10	39573.12	−0.10	*a* ^4^F_1½_— $48284_{2½}^{{}^{\circ}}$	res
2527.200	2	15	39557.59	0.00.	*a* ^4^D_3½_— $54704_{2½}^{{}^{\circ}}$	
2527.552	2	10	39552.08	+0.05	24804_3½_— $64356_{3½}^{{}^{\circ}}$	
2528.560	–?	8	39536.32	+0.05	20455_1½_— $59992_{2½}^{{}^{\circ}}$	
2528.913	5	20	39530.80	+0.05	*a* ^4^D_2½_— $54498_{3½}^{{}^{\circ}}$	res
2529.209	2	8	39526.17	+0.11	26158_4½_— $65684_{5½}^{{}^{\circ}}$	
2530.54	1	4	39505.38	+0.05	24804_3½_— $64310_{2½}^{{}^{\circ}}$	
2530.70	10	8	39502.88	+0.04	*a* ^4^D_1½_— $54137_{1½}^{{}^{\circ}}$	
2530.986	5	50	39498.42	+0.09	20780_4½_— $60278_{4½}^{{}^{\circ}}$	
2532.085	1	1	39481.28	+0.17	23234_4½_— $62715_{4½}^{{}^{\circ}}$	
2532.414	4	7	39476.15	+0.08	20780_4½_— $60256_{3½}^{{}^{\circ}}$	
2532.711	2	5	39471.52	+0.10	19276_2½_— $58747_{1½}^{{}^{\circ}}$	
2532.842	2	3	39469.48	+0.08	25672_2½_— $65141_{2½}^{{}^{\circ}}$	
2532.960	6	15	39467.64	0.00	*a* ^4^P_2½_— $52901_{3½}^{{}^{\circ}}$	
2533.278	2	7	39462.69	+0.23	23803_3½_— $63266_{3½}^{{}^{\circ}}$	
2534.140	5	30	39449.27	+0.07	19442_6½_— $58891_{5½}^{{}^{\circ}}$	7
2534.829	5	50	39438.55	+0.09	20780_4½_— $60218_{5½}^{{}^{\circ}}$	7
2535.178		3	39433.12	+0.08	19276_2½_— $58709_{3½}^{{}^{\circ}}$	
2535.573	1	10	39426.98	+0.25	22139_2½_— $61566_{2½}^{{}^{\circ}}$	*4*
2536.000	6	40	39420.34	0.00	*a* ^4^D_0½_— $52593_{0½}^{{}^{\circ}}$	
2536.622	2	25	39410.67	+0.04	22139_2½_— $61550_{3½}^{{}^{\circ}}$	
2536.789	1	1	39408.07	+0.07	*a* ^4^D_2½_— $54375_{2½}^{{}^{\circ}}$	
2537.133	1	30	39402.73	−0.10	24804_3½_— $64207_{3½}^{{}^{\circ}}$	*7*
2539.313	7	35	39368.91	+0.01	*a* ^4^P_2½_— $52803_{1½}^{{}^{\circ}}$	
2539.869	1	8	39360.29	−0.08	20455_1½_— $59816_{1½}^{{}^{\circ}}$	
2539.919	3	20	39359.52	−0.08	20039_3½_— $59399_{4½}^{{}^{\circ}}$	4
2540.101	1		39356.70	+0.18	22194_3½_— $61550_{3½}^{{}^{\circ}}$	
2540.431	1	4	39351.58	+0.03	*a* ^4^D_3½_— $54498_{3½}^{{}^{\circ}}$	
2540.807		2	39345.76	−0.26	18990_1½_— $58336_{2½}^{{}^{\circ}}$	
2540.925	10*A*	4	39343.93	+0.07	19404_0½_— $58747_{1½}^{{}^{\circ}}$	
2541.063	6	20	39341.80	+0.19	26929_5½_— $66270_{4½}^{{}^{\circ}}$	
2542.598	1	30	39318.05	−0.11	25672_2½_— $64990_{2½}^{{}^{\circ}}$	6
2543.308	2	25	39307.07	−0.02	25209_4½_— $64516_{4½}^{{}^{\circ}}$	6
2544.658		1	39286.22	−0.18	23046_3½_— $62333_{2½}^{{}^{\circ}}$	
2546.283	3	30	39261.15			
2546.790	1	15	39253.33	+0.16	*a* ^4^G_2½_— $55488_{1½}^{{}^{\circ}}$	res
2546.912	1	2	39251.45	+0.15	18000_3½_— $57252_{2½}^{{}^{\circ}}$	
254/.838		5	39237.19	+0.12	20039_3½_— $59276_{3½}^{{}^{\circ}}$	
2548.378	6	20	39228.88	+0.08	*a* ^4^D_3½_— $54375_{2½}^{{}^{\circ}}$	
2548.690	1	6	39224.08	+0.02	25672_2½_— $64896_{3½}^{{}^{\circ}}$	
2549.096	5	15	39217.83	+0.08	*a* ^6^D_2½_— $42390_{3½}^{{}^{\circ}}$	res
2550.10		4	39202.38	+0.21	23235_4½_— $62437_{4½}^{{}^{\circ}}$	
2550.295	5	8	39199.39	+0.07	*a* ^4^F_4½_— $54056_{4½}^{{}^{\circ}}$	
2551.157	1	10	39186.15	−0.17	22139_2½_— $61326_{3½}^{{}^{\circ}}$	
2551.450		15	39181.65	−0.08	*a* ^4^D_0½_— $52355_{0½}^{{}^{\circ}}$	res
2552.249	5	5	39169.38	0.00	*a* ^4^D_2½_— $54137_{1½}^{{}^{\circ}}$	
2552.362	4	40	39167.65	−0.01	26158_4½_— $65326_{5½}^{{}^{\circ}}$	7
2553.168	70*A*	50	39155.29	+0.10	*a* ^4^F_3½_— $52567_{4½}^{{}^{\circ}}$	res
2553.308	1	7	39153.14	−0.14	20780_4½_— $59933_{3½}^{{}^{\circ}}$	
2553.693		1	39147.24	−0.18	25209_4½_— $64356_{3½}^{{}^{\circ}}$	
2554.665	15	70	39132.34	$\left\{ \begin{array}{l} -0.16\\ +0.13 \end{array}\right.$	23955_5½_— $63087_{6½}^{{}^{\circ}}$ 22194_3½_— $61326_{3½}^{{}^{\circ}}$	
2554.864	40	60	39129.30	−0.11	*a* ^6^D_0½_— *z* ${\;}^{6}F_{1½}^{{}^{\circ}}$	
2555.106	40	100	39125.59	−0.09	*a* ^6^D_2½_— $42298_{1½}^{{}^{\circ}}$	
2556.092		6	39110.50	−0.06	19637_2½_— $58747_{1½}^{{}^{\circ}}$	
2557.511		2	39088.80	+0.04	18000_3½_— $57089_{4½}^{{}^{\circ}}$	
2558.582		12	39072.44	+0.26	19637_2½_— $58709_{3½}^{{}^{\circ}}$	
2559.170		1	39063.46	−0.18	22503_1½_— $61566_{2½}^{{}^{\circ}}$	
2559.500	7	40	39058.43	+0.01	*a* ^4^D_2½_— $54026_{2½}^{{}^{\circ}}$	6
2560.788		9	39038.78	0.00	24991_1½_— $64030_{2½}^{{}^{\circ}}$	
2562.244	1	9	39016.60	−0.04	18990_1½_— $58007_{1½}^{{}^{\circ}}$	6
2562.594	1	8	39011.27	+0.17	23955_5½_— $62966_{5½}^{{}^{\circ}}$	
2563.166	20	200	39002.57	−0.01	*a* ^4^G_5½_— $56439_{6½}^{{}^{\circ}}$	4
2563.432		5	38998.52	+0.30	25209_4½_— $64207_{4½}^{{}^{\circ}}$	
2563.534	1	4	38996.97	−0.12	27273_3½_— $66270_{4½}^{{}^{\circ}}$	
2563.914	12	30	38991.19	−0.06	*a* ^4^F_2½_— $50292_{2½}^{{}^{\circ}}$	6 res
2564.424		8	38983.43	−0.10	24804_3½_— $63788_{3½}^{{}^{\circ}}$	7
2565.834		7	38962.01	+0.01	24918_1½_— $63880_{2½}^{{}^{\circ}}$	
2567.320		1	38939.46	+0.03	*a* ^4^G_5½_— $56376_{5½}^{{}^{\circ}}$	
2567.620	10	30	38934.92	−0.05	*a* ^6^S_2½_— $46355_{2½}^{{}^{\circ}}$	6 res
2568.108	2	10	38927.52	+0.06	*a* ^4^G_2½_— $55162_{2½}^{{}^{\circ}}$	
2568.850	10	30	38916.28	+0.03	19070_4½_— $57986_{4½}^{{}^{\circ}}$	6
2569.123	1	3	38912.14	0.00	23803_3½_— $62715_{4½}^{{}^{\circ}}$	
2569.298	8	80	38909.49	−0.03	*a* ^4^D_3½_— $54056_{4½}^{{}^{\circ}}$	5
2570.701		2	38888.26	−0.28	24991_1½_— $63880_{2½}^{{}^{\circ}}$	
2571.459	50	150	38876.79	−0.14	*a* ^6^D_2½_— *z* ${\;}^{6}F_{2½}^{{}^{\circ}}$	res
2571.632	3	15	38874.18	−0.11	18000_3½_— $56874_{2½}^{{}^{\circ}}$	
2572.240	8	45	38864.99	0.00	20534_5½_— $59399_{4½}^{{}^{\circ}}$	
2572.366	8	30	38863.08	−0.24	*a* ^4^F_3½_— $52275_{3½}^{{}^{\circ}}$	
2572.572		25	38859.97	−0.50	25169_1½_— $64030_{2½}^{{}^{\circ}}$	
2573.605		9	38844.38	−0.08	26158_4½_— $65003_{4½}^{{}^{\circ}}$	
2573.820	3	12	38841.13	−0.05	*a* ^4^P_2½_— $52275_{3½}^{{}^{\circ}}$	
2573.952	15*A*	7	38839.14	−0.09	*a* ^4^G_4½_— $53924_{4½}^{{}^{\circ}}$	
2576.168	*7*	30	38805.73	−0.08	*a* ^4^D_1½_— $53440_{0½}^{{}^{\circ}}$	
2576.372	8	40	38802.66	−0.04	*a* ^4^G_3½_— $55392_{4½}^{{}^{\circ}}$	
2577.308		5	38788.57	−0.05	*a* ^4^D_1½_— $53422_{1½}^{{}^{\circ}}$	
2578.695	8	3	38767.71	−0.20	18000_3½_— $56768_{3½}^{{}^{\circ}}$	
2579.266	20	70	38759.13	−0.03	23955_5½_— $62714_{6½}^{{}^{\circ}}$	7
2579.497		20	38755.66	$\left\{ \begin{array}{l} -0.32\\ +0.20 \end{array}\right.$	*a* ^4^P_0½_— $47588_{1½}^{{}^{\circ}}$ 26029_5½_— $65684_{5½}^{{}^{\circ}}$	
2579.542	20	100	38754.98	−0.01	*a* ^4^S_2½_— $46175_{3½}^{{}^{\circ}}$	res
2581.140	2	8	38730.99	−0.09	19276_2½_— $58007_{1½}^{{}^{\circ}}$	
2581.206	18	30	38730.00	−0.02	*a* ^6^D_4½_— *z* ${\;}^{6}F_{3½}^{{}^{\circ}}$	
2582.527	1	5	38710.19	−0.04	25169_1½_— $63880_{2½}^{{}^{\circ}}$	
2582.746	1	4	38706.90	+0.01	22194_3½_— $60900_{2½}^{{}^{\circ}}$	
2583.520	3	5	38695.31	−0.04	*a* ^4^D_1½_— $53329_{1½}^{{}^{\circ}}$	6
2584.236	12*A*	5	38684.59	+0.05	25672_2½_— $64356_{3½}^{{}^{\circ}}$	
2585.146		4	38670.97	+0.03	29341_4½_— $68012_{5½}^{{}^{\circ}}$	
2585.934	6	30	38659.18	−0.06	19070_4½_— $57729_{3½}^{{}^{\circ}}$	
2586.350	8	25	38652.97	+0.05	*a* ^4^P_2½_— $52087_{2½}^{{}^{\circ}}$	6
2586.586	1	5	38649.44	−0.14	*a* ^4^P_1½_— $49242_{2½}^{{}^{\circ}}$	
2587.367		6	38637.78	−0.06	25672_2½_— $64310_{2½}^{{}^{\circ}}$	
2587.676	2	9	38633.17	−0.03	23803_3½_— $62437_{4½}^{{}^{\circ}}$	
2589.171	30	90	38610.86	−0.08	*a* ^6^D_4½_— $44758_{4½}^{{}^{\circ}}$	
2589.661	4	9	38603.56	+0.04	19404_0½_— $58007_{1½}^{{}^{\circ}}$	
2591.229		2	38580.20	+0.02	19276_2½_— $57856_{2½}^{{}^{\circ}}$	
2591.492	14	12	38576.28	−0.04	*a* ^6^D_0½_— *z* ${\;}^{2}S_{0½}^{{}^{\circ}}$	
2591.738	1	4	38572.62	−0.01	*a* ^4^G_3½_— $55162_{2½}^{{}^{\circ}}$	
2592.458	3	3	38561.91	−0.07	*a* ^4^P_1½_— $49154_{0½}^{{}^{\circ}}$	
2593.686	1	2	38543.65	−0.05	18000_3½_— $56544_{2½}^{{}^{\circ}}$	
2594.645		2	38529.41	+0.05	23803_3½_— $62333_{2½}^{{}^{\circ}}$	
2595.506	2	7	38516.63	+0.09	22139_2½_— $60656_{2½}^{{}^{\circ}}$	7
2595.580	5	5	38515.53	−0.37	28187_6½_— $66703_{5½}^{{}^{\circ}}$	
2595.764	3	8	38512.80	+0.05	*a* ^4^F_4½_— $53369_{4½}^{{}^{\circ}}$	6
2596.867	3	15	38496.44	+0.01	20780_4½_— $59276_{3½}^{{}^{\circ}}$	7
2597.866	2	25	38481.64	0.00	23955_5½_— $62437_{4½}^{{}^{\circ}}$	7
2598.672	7*A*	7	38469.71	$\left\{ \begin{array}{l} -0.06\\ -0.01 \end{array}\right.$	*a* ^4^G_2½_— $54704_{2½}^{{}^{\circ}}$ *a* ^4^G_4½_— $55022_{3½}^{{}^{\circ}}$	
2598.748	20	35	38468.58	−0.10	*a* ^4^F_1½_— $47179_{1½}^{{}^{\circ}}$	
2599.174	1–	1	38462.27	−0.16	22194_3½_— $60656_{2½}^{{}^{\circ}}$	
2599.652	3	7	38455.20	+0.04	*a* ^4^D_2½_— $53422_{1½}^{{}^{\circ}}$	
2599.772	4	20	38453.42	+0.02	10276_2½_— $57729_{3½}^{{}^{\circ}}$	res
2601.141	1	6	38433.19	0.00	*a* ^4^G_3½_— $55022_{3½}^{{}^{\circ}}$	res
2601.430	8	30	38428.92	−0.01	*a* ^4^P_2½_— $51863_{3½}^{{}^{\circ}}$	
2602.166	2	1	38418.06	+0.03	*a* ^6^D_1½_— $39936_{4½}^{{}^{\circ}}$	
2602.516	12	75	38412.89	−0.05	18000_3½_— $56413_{4½}^{{}^{\circ}}$	
2603.018	20	120	38405.48	+0.04	*a* ^4^G_4½_— $54958_{5½}^{{}^{\circ}}$	7
2603.609		3	38396.76	−0.30	26929_5½_— $65326_{5½}^{{}^{\circ}}$	
2604.042	2	6	38390.38	+0.04	*a* ^4^P_1½_— $48982_{1½}^{{}^{\circ}}$	6
2605.410	–?	5	38370.23	$\left\{ \begin{array}{l} -0.02\\ +0.01 \end{array}\right.$	*a* ^4^D_2½_— $53338_{3½}^{{}^{\circ}}$ 19637_2½_— $58007_{1½}^{{}^{\circ}}$	
2605.973	7	15	38361.94	+0.05	*a* ^4^D_2½_— $53329_{1½}^{{}^{\circ}}$	res
2606.227		2?	38358.20	+0.02	25672_2½_— $64030_{2½}^{{}^{\circ}}$	
2606.273	2	20	38357.52	$\left\{ \begin{array}{l} -0.02\\ +0.01 \end{array}\right.$	26158_4½_— $64516_{4½}^{{}^{\circ}}$ 20534_5½_— $58891_{5½}^{{}^{\circ}}$	
2606.472	4	40	38354.59	0.00	23234_4½_— $61589_{5½}^{{}^{\circ}}$	
2606.974	4	12	38347.21	−0.07	*a* ^4^P_0½_— $47179_{1½}^{{}^{\circ}}$	
2607.822		20	38334.74	+0.04	22139_2½_— $60474_{2½}^{{}^{\circ}}$	7
2608.440		5	38325.66	−0.04	28377_3½_— $66703_{5½}^{{}^{\circ}}$	
2609.116	2	7	38315.72	−0.01	23234_4½_— $61550_{3½}^{{}^{\circ}}$	
2609.250	4	40	38313.76	+0.02	23046_3½_— $61360_{4½}^{{}^{\circ}}$	5
2610.370	1	3	38297.32	−0.08	20039_3½_— $58336_{2½}^{{}^{\circ}}$	
2611.264	1	8	38284.21	+0.04	22139_2½_— $60424_{3½}^{{}^{\circ}}$	
2611.510		1	38280.60	+0.01	22194_3½_— $60474_{2½}^{{}^{\circ}}$	
2611.592	1	6	38279.40	−0.09	23046_3½_— $61326_{3½}^{{}^{\circ}}$	6
2612.667	4	15	38263.65	−0.08	*a* ^4^G_2½_— $54498_{3½}^{{}^{\circ}}$	
2612.848		2	38261.00	−0.04	18990_1½_— $57252_{2½}^{{}^{\circ}}$	
2614.956		2	38230.16	+0.10	22194_3½_— $60424_{3½}^{{}^{\circ}}$	
2615.446	20	80	38223.00	+0.05	*a* ^4^D_3½_— $53369_{4½}^{{}^{\circ}}$	
2615.701	3	15	38219.27	−0.05	19637_2½_— $57856_{2½}^{{}^{\circ}}$	
2617.162	1	2	38197.94	−0.02	26158_4½_— $64356_{3½}^{{}^{\circ}}$	
2617.636		5	38191.02	−0.03	*a* ^4^D_3½_— $53838_{3½}^{{}^{\circ}}$	6
2618.072	1	7	38184.66	−0.27	24804_3½_— $62989_{2½}^{{}^{\circ}}$	
2619.18	40*A*	15	38168.50	−0.14	*a* ^4^D_1½_— $52803_{1½}^{{}^{\circ}}$	
2620.215	50*A*	60	38153.43	−0.02	22503_1½_— $60656_{2½}^{{}^{\circ}}$	4
2620.757	*7*	30	38145.54	−0.16	*a* ^4^D_2½_— $53113_{2½}^{{}^{\circ}}$	res
2621.074		2	38140.93	−0.05	*a* ^4^G_2½_— $54375_{2½}^{{}^{\circ}}$	
2621.601	5	12	38133.26	−0.01	*a* ^6^S_2½_— $45558_{1½}^{{}^{\circ}}$	
2621.826		1	38129.99	+0.29	26227_2½_— $64356_{3½}^{{}^{\circ}}$	
2622.778	1	5	38116.15	−0.05	23450_2½_— $61566_{2½}^{{}^{\circ}}$	
2622.873	1	2	38114.77	−0.17	*a* ^4^G_3½_— $54704_{2½}^{{}^{\circ}}$	
2623.114	6	50	38111.27	−0.09	20780_4½_— $58891_{5½}^{{}^{\circ}}$	
2623.890	2	8	38100.00	−0.10	23450_2½_— $61550_{3½}^{{}^{\circ}}$	res
2624.401		3	38092.58	+0.04	19637_2½_— $57729_{3½}^{{}^{\circ}}$	
2624.488	2	15	38091.32	−0.10	23234_4½_— $61326_{3½}^{{}^{\circ}}$	
2624.952		4	38084.59	−0.04	22194_3½_— $60278_{4½}^{{}^{\circ}}$	
2625.058		3	38083.05	+0.05	26227_2½_— $64310_{2½}^{{}^{\circ}}$	
2625.863		4	38071.38	−0.12	24918_1½_— $62989_{2½}^{{}^{\circ}}$	
2626.486		12	38062.35	−0.02	22194_3½_— $60256_{3½}^{{}^{\circ}}$	6
2626.851		3	38057.06	+0.04	25209_4½_— $63266_{3½}^{{}^{\circ}}$	
2627.722	4	10	38044.44	−0.08	*a* ^4^F_4½_— $52901_{3½}^{{}^{\circ}}$	
2628.045	1	8	38039.76	0.00	26929_5½_— $64969_{5½}^{{}^{\circ}}$	
2628.996	7	35	38026.00	−0.07	*a* ^4^F_3½_— $51438_{2½}^{{}^{\circ}}$	4
2629.496	1	5	38018.78	0.00	19070_4½_— $57089_{4½}^{{}^{\circ}}$	6
2630.177	3	7	38008.93	−0.07	23046_3½_— $61055_{4½}^{{}^{\circ}}$	7
2630.384	3	20	38005.94	0.00	23234_4½_— $61240_{5½}^{{}^{\circ}}$	
2630.534	6	9	38003.78	−0.15	*a* ^4^P_2½_— $51438_{2½}^{{}^{\circ}}$	res
2630.946		2	37997.83	−0.21	24991_1½_— $62989_{2½}^{{}^{\circ}}$	
2632.485	35*A*	7	37975.61	+0.13	19276_2½_— $57252_{2½}^{{}^{\circ}}$	
2632.768	7?	7	37971.53	−0.08	22503_1½_— $60474_{2½}^{{}^{\circ}}$	
2633.886	4	25	37955.41	+0.06	*a* ^4^G_5½_— $55392_{4½}^{{}^{\circ}}$	
2634.578	6	35	37945.45	+0.02	*a* ^4^G_4½_— $54498_{3½}^{{}^{\circ}}$	
2635.379	6	30	37933.91	−0.01	*a* ^4^D_2½_— $52901_{3½}^{{}^{\circ}}$	
2635.723		1	37928.96	−0.22	20780_4½_— $58709_{3½}^{{}^{\circ}}$	
2636.772	1	2	37913.87	−0.14	*a* ^4^F_1½_— $46625_{0½}^{{}^{\circ}}$	
2636.953	1	20	37911.27	−0.04	24804_3½_— $62715_{4½}^{{}^{\circ}}$	
2637.209	–?	10	37907.59	+0.09	20780_4½_— $58687_{4½}^{{}^{\circ}}$	
2637.576	15*A*	25	37902.32	−0.04	*a* ^4^G_2½_— $54137_{1½}^{{}^{\circ}}$	res
2638.220		7	37893.06	−0.09	28377_5½_— $66270_{4½}^{{}^{\circ}}$	
2638.870		1	37883.73	−0.30	18990_1½_— $56874_{2½}^{{}^{\circ}}$	
2639.052	1	3	37881.12	+0.07	20455_1½_— $58336_{2½}^{{}^{\circ}}$	
2639.432		1	37875.67	−0.12	23450_2½_— $61326_{3½}^{{}^{\circ}}$	
2641.076	2	8	37852.09	−0.14	22139_2½_— $59992_{2½}^{{}^{\circ}}$	6
2643.086	–?	10	37823.31	−0.13	*a* ^4^F_2½_— $49124_{3½}^{{}^{\circ}}$	
2643.253	1	10	37820.92	−0.01	23234_4½_— $61055_{4½}^{{}^{\circ}}$	
2643.296	4	30	37820.31	+0.01	*a* ^4^P_2½_— $51254_{1½}^{{}^{\circ}}$	
2645.692	25*A*	7	37786.06	−0.09	*a* ^4^G_3½_— $54375_{2½}^{{}^{\circ}}$	
2647.309	1	4	37762.98	+0.12	23803_3½_— $61566_{2½}^{{}^{\circ}}$	
2647.726	7	70	37757.03	−0.19	25209_4½_— $62966_{5½}^{{}^{\circ}}$	4
2647.894	1	2	37754.63	−0.09	*a* ^4^D_3½_— $52901_{3½}^{{}^{\circ}}$	
2648.453		2	37746.67	−0.09	23803_3½_— $61550_{3½}^{{}^{\circ}}$	
2648.958		2	37739.47	−0.11	22194_3½_— $59933_{3½}^{{}^{\circ}}$	
2649.682	1	10	37729.16	−0.01	22139_2½_— $59869_{3½}^{{}^{\circ}}$	
2650.276	3	7	37720.70	−0.05	*a* ^4^D_1½_— $52355_{0½}^{{}^{\circ}}$	
2650.580	1	3	37716.38	−0.08	27273_3½_— $64990_{2½}^{{}^{\circ}}$	
2651.035	1	8	37709.90	−0.03	*a* ^4^F_4½_— $52567_{4½}^{{}^{\circ}}$	6
2651.881	4	30	37697.87	−0.06	19070_4½_— $56768_{3½}^{{}^{\circ}}$	
2542.298	1	3	37691.95	−0.01	*a* ^4^P_1½_— $48284_{2½}^{{}^{\circ}}$	
2652.433		4	37690.03	−0.15	20039_3½_— $57729_{3½}^{{}^{\circ}}$	
2653.015	10*A*	8	37681.76	−0.02	*a* ^4^F_2½_— $48982_{1½}^{{}^{\circ}}$	
2653.424	7	35	37675.96	+0.04	*a* ^4^G_4½_— $54229_{5½}^{{}^{\circ}}$	
2653.568	10	35	37673.91	−0.04	*a* ^6^D_3½_— $42390_{3½}^{{}^{\circ}}$	
2654.841	1	5	37655.84	+0.09	19276_2½_— $56932_{1½}^{{}^{\circ}}$	
2655.485	3	25	37646.71	−0.07	23955_5½_— $61602_{6½}^{{}^{\circ}}$	
2655.667	15	20	37644.13	−0.01	*a* ^4^F_1½_— $46355_{2½}^{{}^{\circ}}$	
2656.027		10	27639.03	−0.06	28631_3½_— $66270_{4½}^{{}^{\circ}}$	
2656.435	–?	8	37633.25	−0.04	*a* ^4^F_3½_— $51045_{3½}^{{}^{\circ}}$	
2656.706		10	37629.41	−0.05	26158_4½_— $63788_{3½}^{{}^{\circ}}$	
2657.745	1	6	37614.71	+0.09	19637_2½_— $57252_{2½}^{{}^{\circ}}$	
2658.036	25	100	37610.59	−0.04	*a* ^6^D_1½_—*z* ${\;}^{6}F_{1½}^{{}^{\circ}}$	res
2659.171	1?	10	37594.53	+0.39	25672_3½_— $63266_{3½}^{{}^{\circ}}$	
2659.703	1	15	37587.02	−0.01	26929_5½_— $64516_{4½}^{{}^{\circ}}$	
2661.852	1	20	37556.67	−0.03	23803_3½_— $61360_{4½}^{{}^{\circ}}$	
2662.214	3	30	37551.57	−0.10	20155_1½_— $58007_{1½}^{{}^{\circ}}$	res
2663.874	2	15	37528.17	−0.02	19404_0½_— $56932_{1½}^{{}^{\circ}}$	
2664.346	80	200	37521.52	−0.04	*a* ^4^G_5½_— $54958_{5½}^{{}^{\circ}}$	
2665.644	2	8	27503.25	−0.15	*a* ^4^G_4½_— $54056_{4½}^{{}^{\circ}}$	
2666.086	1	40	37497.03	−0.17	28187_6½_— $65684_{5½}^{{}^{\circ}}$	
2666.446	8	?	37491.97	−0.12	19276_2½_— $56768_{3½}^{{}^{\circ}}$	
2666.493	20	60	37491.31	+0.11	*a* ^4^S_2½_— $44911_{1½}^{{}^{\circ}}$	
2668.245	1	5	37466.69	−0.18	*a* ^4^G_3½_— $54056_{4½}^{{}^{\circ}}$	
2668.961	5	5	37456.64	−0.11	*a* ^6^S_2½_— *z* ${\;}^{6}F_{3½}^{{}^{\circ}}$	
2669.250	10	25	37452.59	−0.07	*a* ^4^D_1½_— $52087_{2½}^{{}^{\circ}}$	
2669.371	5	30	37450.89	−0.20	*a* ^4^F_3½_— $50863_{4½}^{{}^{\circ}}$	
2670.395	10	50	37436.52	−0.04	*a* ^4^G_3½_— $54026_{2½}^{{}^{\circ}}$	
2671.040		2	37427.49	−0.38	23046_3½_— $60474_{2½}^{{}^{\circ}}$	
2671.580	8	18	37419.92	−0.21	*a* ^4^D_3½_— $52567_{4½}^{{}^{\circ}}$	
2672.647		15	37404.98	−0.16	23955_5½_— $61360_{4½}^{{}^{\circ}}$	
2672.956	1	3	37400.66	−0.11	20455_1½_— $57856_{2½}^{{}^{\circ}}$	
2673.608	20	60	37391.54	−0.13	18000_3½_— $55392_{4½}^{{}^{\circ}}$	res
2674.471		4	37379.48	0.00	30633_4½_— $68012_{5½}^{{}^{\circ}}$	
2674.628		10	37377.29	−0.05	23046_3½_— $60424_{3½}^{{}^{\circ}}$	
2675.734	1	20	37361.84	−0.10	29341_4½_— $66703_{5½}^{{}^{\circ}}$	
2677.579	3	6	37336.10	−0.12	19276_2½_— $56612_{3½}^{{}^{\circ}}$	
2677.796	20	60	37333.07	−0.06	*a* ^6^D_3½_— *z* ${\;}^{6}F_{2½}^{{}^{\circ}}$	
2679.638	20	70	37307.41	−0.05	*a* ^4^D_2½_— $52275_{3½}^{{}^{\circ}}$	
2679.758	3	18	37305.74	−0.03	19070_4½_— $56376_{5½}^{{}^{\circ}}$	
2680.546		10	37294.77	−0.12	19637_2½_— $56932_{1½}^{{}^{\circ}}$	
2681.568		2	37280.55	+0.05	22535_0½_— $59816_{1½}^{{}^{\circ}}$	
2681.730		1	37278.30	+0.14	26920_5½_— $64207_{4½}^{{}^{\circ}}$	
2683.226	8	80	37257.52	−0.05	*a* ^4^D_0½_— $50430_{1½}^{{}^{\circ}}$	
2683.512	1	35	37253.56			
2683.632	4	30	37251.89	−0.07	23803_3½_— $61055_{4½}^{{}^{\circ}}$	
2684.301	12	20	37242.60	+0.09	27273_3½_— $64516_{4½}^{{}^{\circ}}$	
2685.068	12	25	37231.96	+0.05	23046_3½_— $60278_{4½}^{{}^{\circ}}$	
2685.366	6	12	37227.84	+0.08	25209_4½_— $62437_{4½}^{{}^{\circ}}$	
2686.946	4	100	37205.95	−0.06	23450_2½_— $60656_{2½}^{{}^{\circ}}$	res
2687.000	5	70	37205.20	−0.06	22194_3½_— $59399_{4½}^{{}^{\circ}}$	
2688.230	7	35	37188.17	+0.03	*a* ^4^G_2½_— $53422_{1½}^{{}^{\circ}}$	
2690.036		2	37163.21	−0.12	25169_1½_— $62333_{2½}^{{}^{\circ}}$	
2690.153	–?	1	37161.59	−0.01	18000_3½_— $55162_{2½}^{{}^{\circ}}$	
2690.710	2	35	37153.90			
2691.952	2	5	37136.74	−0.10	22139_2½_— $59276_{3½}^{{}^{\circ}}$	
2692.358	4	18	37131.14	−0.09	19637_2½_— $56768_{3½}^{{}^{\circ}}$	
2693.228		1	37119.14	−0.06	*a* ^4^D_2½_— $52087_{2½}^{{}^{\circ}}$	
2694.080		4?	37107.40	−0.16	26158_4½_— $63266_{3½}^{{}^{\circ}}$	
2694.382	20	60	37103.24	+0.01	*a* ^4^G_2½_— $53338_{3½}^{{}^{\circ}}$	
2694.594	6	70	37100.32	−0.08	23955_5½_— $61055_{4½}^{{}^{\circ}}$	
2694.828	2	6	37097.10	−0.03	23803_3½_— $60900_{2½}^{{}^{\circ}}$	
2694.994	6	30	37094.82	−0.05	*a* ^4^G_2½_— $53329_{1½}^{{}^{\circ}}$	
2695.111		1	37093.21	−0.13	18990_1½_— $56081_{1½}^{{}^{\circ}}$	
2695.874	3	10	37082.72	−0.01	22194_3½_— $59276_{3½}^{{}^{\circ}}$	
2696.914	2	35	37068.41			6
2697.714	80	160	37057.42	−0.12	*a* ^6^D_1½_— *z* ${\;}^{2}S_{0½}^{{}^{\circ}}$	res
2698.265	1	1?	37049.86	+0.14	20039_3½_— $57089_{4½}^{{}^{\circ}}$	
2698.706	1	3	37043.80	−0.04	23234_4½_— $60278_{4½}^{{}^{\circ}}$	
2699.041	8	12	37039.21	−0.09	26227_2½_— $63266_{3½}^{{}^{\circ}}$	
2699.270	1	10	37036.06	−0.08	27273_3½_— $64310_{2½}^{{}^{\circ}}$	
2700.320	3	50	37021.67	+0.09	23234_4½_— $60256_{3½}^{{}^{\circ}}$	res
2701.036	8*A*	10	37011.85	−0.29	28631_3½_— $56443_{3½}^{{}^{\circ}}$	
2701.485	20	60	37005.70	−0.11	*a* ^4^F_4½_— $51863_{3½}^{{}^{\circ}}$	
2702.115	25	250	36997.07	+0.01	19442_6½_— $56439_{6½}^{{}^{\circ}}$	6
2702.188	4	?	36996.07	−0.05	*a* ^4^P_1½_— $47588_{1½}^{{}^{\circ}}$	
2703.066	8	60	36984.05	+0.08	23234_4½_— $60218_{5½}^{{}^{\circ}}$	
2703.118	8	10	36983.36	−0.04	*a* ^4^F_2½_— $48284_{2½}^{{}^{\circ}}$	
2703.467	12	120	36978.57	+0.13	31100_5½_— $68078_{6½}^{{}^{\circ}}$	5
2703.698	1	5	36975.41	+0.05	19637_2½_— $56612_{3½}^{{}^{\circ}}$	
2703.829		1	36973.62	−0.02	23450_2½_— $60424_{3½}^{{}^{\circ}}$	
2705.599	10	35	36949.43	−0.11	20780_4½_— $57729_{3½}^{{}^{\circ}}$	
2705.895		12	36945.40	0.00	23046_3½_— $59992_{2½}^{{}^{\circ}}$	
2706.702	6	?	36934.38	−0.01	*a* ^6^S_2½_— $44354_{2½}^{{}^{\circ}}$	
2706.733		50	36933.95	+0.04	19442_6½_— $56376_{5½}^{{}^{\circ}}$	
2707.067		25	36929.40	+0.01	29341_4½_— $66270_{4½}^{{}^{\circ}}$	6
2708.360	4*A*	2	36911.77	−0.27	31100_5½_— $68012_{5½}^{{}^{\circ}}$	
2709.582	20	80	36895.12	−0.09	*a* ^4^D_2½_— $51863_{3½}^{{}^{\circ}}$	
2710.678	2	1	36880.20	−0.17	*a* ^4^F_3½_— $50292_{2½}^{{}^{\circ}}$	
2710.792	5	40	36878.65	−0.03	*a* ^4^G_2½_— $53113_{2½}^{{}^{\circ}}$	6 res
2711.336		3	36871.26	−0.16	28118_2½_— $64990_{2½}^{{}^{\circ}}$	
2712.704	5	50	36852.66	−0.01	23803_3½_— $60656_{2½}^{{}^{\circ}}$	
2713.478	4	2	36842.15	−0.29	*a* ^4^F_1½_— $45553_{1½}^{{}^{\circ}}$	
2714.016		1	36834.85	−0.40	20039_3½_— $56874_{2½}^{{}^{\circ}}$	
2714.950	1	5	36822.18	−0.16	23046_3½_— $59869_{3½}^{{}^{\circ}}$	
2715.346	25	80	36816.81	−0.02	*a* ^4^G_4½_— $53369_{4½}^{{}^{\circ}}$	6
2716.020	2	3	36807.67	$\left\{ \begin{array}{l} -0.11\\ -0.09 \end{array}\right.$	19276_2½_— $56084_{1½}^{{}^{\circ}}$ 26158_4½_— $62966_{5½}^{{}^{\circ}}$	
2716.147	3	15	36805.95	0.00	23450_2½_— $60256_{3½}^{{}^{\circ}}$	
2716.322	30	80	36803.58	−0.09	*a* ^4^D_1½_— $51438_{2½}^{{}^{\circ}}$	
2716.890	35*A*	35	36795.90	−0.17	20455_1½_— $57252_{2½}^{{}^{\circ}}$	
2717.180	10	40	36791.96	−0.08	*a* ^4^G_5½_— $54229_{5½}^{{}^{\circ}}$	6 res
2717.702	3	18	36784.90	−0.03	*a* ^4^G_4½_— $53338_{3½}^{{}^{\circ}}$	
2718.044	30	120	36780.26	−0.04	*a* ^4^G_3½_— $53369_{4½}^{{}^{\circ}}$	4
2718.257		2	36777.38	+0.06	28118_2½_— $64896_{3½}^{{}^{\circ}}$	
2719.232	8	3	36764.20	−0.09	*a* ^6^D_2½_— $59936_{2½}^{{}^{\circ}}$	
2719.392		10	36762.03	0.00	24804_3½_— $61566_{2½}^{{}^{\circ}}$	
2719.798		1	36756.54	+0.06	27273_3½_— $64030_{2½}^{{}^{\circ}}$	
2720.404	6	30	36748.36	−0.04	*a* ^4^G_3½_— $53338_{3½}^{{}^{\circ}}$	6
2720.594		40	36745.80	+0.04	*a* ^4^F_1½_— $45457_{0½}^{{}^{\circ}}$	
2721.850	4	15	36728.83	−0.04	20039_3½_— $56768_{3½}^{{}^{\circ}}$	
2722.805	20	70	36715.95	−0.06	*a* ^4^D_3½_— $51863_{3½}^{{}^{\circ}}$	6
2723.704	3	5	36703.84	−0.07	18000_3½_— $54704_{2½}^{{}^{\circ}}$	
2724.081	5	60	36698.75	−0.04	23234_4½_— $59933_{3½}^{{}^{\circ}}$	
2725.457		8	36680.23	+0.01	19404_0½_— $56084_{1½}^{{}^{\circ}}$	
2726.445	3	4	36666.94	+0.04	*a* ^4^G_2½_— $52901_{3½}^{{}^{\circ}}$	
2728.880	1	6	36634.22	−0.05	23234_4½_— $59869_{3½}^{{}^{\circ}}$	
2729.532	1	6	36625.48	+0.08	28377_5½_— $65003_{4½}^{{}^{\circ}}$	
2729.620	25	75	36624.30	−0.06	*a* ^4^P_0½_— $45457_{0½}^{{}^{\circ}}$	res
2729.936	5	30	36620.06	+0.02	*a* ^4^D_1½_— $51254_{1½}^{{}^{\circ}}$	
2730.846	3	8	36607.86	−0.11	22139_2½_— $58747_{1½}^{{}^{\circ}}$	
2732.078	2	8	36591.35	+0.05	28377_5½_— $64969_{5½}^{{}^{\circ}}$	6
2732.378	4	4	36587.33	−0.09	*a* ^4^P_1½_— $47179_{1½}^{{}^{\circ}}$	6
2733.448	5	35	36573.01	+0.01	20039_3½_— $56612_{3½}^{{}^{\circ}}$	6
2733.704		3	36569.58	−0.01	22139_2½_— $58709_{3½}^{{}^{\circ}}$	
2734.627		3	36557.24	0.00	26158_4½_— $62715_{4½}^{{}^{\circ}}$	
2734.737	5	40	36555.77	−0.10	24804_3½_— $61360_{4½}^{{}^{\circ}}$	
2735.784	2	10	36541.78	+0.08	23450_2½_— $59992_{2½}^{{}^{\circ}}$	6
2737.135	1	3	36523.74	−0.11	*a* ^4^G_3½_— $53113_{2½}^{{}^{\circ}}$	
2737.756	2	5	36515.46	−0.02	22194_3½_— $58709_{3½}^{{}^{\circ}}$	
2737.840		3	36514.34	0.00	27273_3½_— $63788_{3½}^{{}^{\circ}}$	
2738.962	1	5	36499.38	+0.06	28491_1½_— $64990_{2½}^{{}^{\circ}}$	7
2739.080	4	4	36497.81	−0.06	18000_3½_— $54498_{3½}^{{}^{\circ}}$	
2739.140	4	15	36497.01	−0.04	18990_1½_— $54881_{1½}^{{}^{\circ}}$	7
2739.384	6	30	36493.76	−0.04	22194_3½_— $58687_{4½}^{{}^{\circ}}$	
2740.187	6	20	36483.06	−0.10	23450_2½_— $59933_{3½}^{{}^{\circ}}$	
2740.801	20	200	36474.90	+0.03	23803_3½_— $60278_{4½}^{{}^{\circ}}$	4
2742.471	15	30	36452.68	+0.07	23803_3½_— $60256_{3½}^{{}^{\circ}}$	6 res
2742.903	8	40	36446.94	+0.02	19637_2½_— $56084_{1½}^{{}^{\circ}}$	
2745.036	2	30	36418.62	−0.02	23450_2½_— $59869_{3½}^{{}^{\circ}}$	4 res
2748.312	20	15	36375.21	+0.09	18000_3½_— $54375_{2½}^{{}^{\circ}}$	res
2748.418	2	12	36373.82	−0.08	20039_3½_— $56413_{4½}^{{}^{\circ}}$	
2750.023	1	9	36352.61	+0.07	23046_3½_— $59399_{4½}^{{}^{\circ}}$	
2750.325	10	20	36348.60	0.00	*a* ^4^G_4½_— $52901_{3½}^{{}^{\circ}}$	res
2750.728	2	15	36343.28	+0.04	29341_4½_— $65684_{5½}^{{}^{\circ}}$	
2750.880	3	12	36341.28	−0.04	25209_4½_— $61550_{3½}^{{}^{\circ}}$	
2752.242	5	30	36323.28	−0.03	23955_5½_— $60278_{4½}^{{}^{\circ}}$	7
2752.360	1?	2	36321.72	+0.03	19070_4½_— $55392_{4½}^{{}^{\circ}}$	
2753.094		6	36312.03	−0.04	*a* ^4^G_3½_— $52901_{3½}^{{}^{\circ}}$	
2753.320	6	25	36309.05	−0.03	20780_4½_— $57089_{4½}^{{}^{\circ}}$	7
2753.826		6	36302.37	−0.07	29341_4½_— $65644_{3½}^{{}^{\circ}}$	
2754.695	1	2	36290.93	−0.08	*a* ^4^D_3½_— $51438_{2½}^{{}^{\circ}}$	
2755.036	1	3	36286.44	−0.14	*a* ^4^D_2½_— $51254_{1½}^{{}^{\circ}}$	
2755.661		6	36278.21	−0.09	26158_4½_— $62437_{4½}^{{}^{\circ}}$	
2756.776	2	20	36263.54	+0.10	23955_5½_— $60218_{5½}^{{}^{\circ}}$	6
2757.7LO		6	36251.26	+0.13	24804_3½_— $61055_{4½}^{{}^{\circ}}$	
2758.182		4	36245.05	+0.17	22503_1½_— $58747_{1½}^{{}^{\circ}}$	
2758.333	18	20	36234.07	−0.04	*a* ^6^D_4½_— $42390_{3½}^{{}^{\circ}}$	res
2759.338	1	10	36229.87	−0.14	23046_3½_— $59276_{3½}^{{}^{\circ}}$	7
2760.691	3	15	36212.11	−0.03	22535_0½_— $58747_{1½}^{{}^{\circ}}$	6
2760.742	12*A*	10	36211.44	−0.05	19276_2½_— $55488_{1½}^{{}^{\circ}}$	
2761.587	40	100	36200.37	0.00	*a* ^4^F_1½_— $44911_{1½}^{{}^{\circ}}$	res
2761.837	2	6	36197.09	+0.08	22139_2½_— $58336_{2½}^{{}^{\circ}}$	
2762.499	6	15	36188.42	+0.06	23803_3½_— $59992_{2½}^{{}^{\circ}}$	
2764.263	200	400	36165.33	−0.02	*a* ^6^G_0½_— *z* ${\;}^{6}F_{0½}^{{}^{\circ}}$	
2764.768	8	50	36158.72	+0.16	26929_5½_— $63087_{6½}^{{}^{\circ}}$	5
2765.340	2	10	36151.24	−0.02	25209_4½_— $61360_{4½}^{{}^{\circ}}$	6
2765.986		1	36142.80	−0.10	22194_3½_— $58336_{2½}^{{}^{\circ}}$	
2766.318	1	35	36138.46	−0.11	28377_5½_— $64516_{4½}^{{}^{\circ}}$	7
2766.981	4	35	36129.80	−0.02	23803_3½_— $59933_{3½}^{{}^{\circ}}$	6
2767.967	4	25	36116.93	−0.08	25209_4½_— $61326_{3½}^{{}^{\circ}}$	
2768.326	30	50	36112.25	0.00	*a* ^4^F_2½_— $47413_{2½}^{{}^{\circ}}$	6
2770.496	2	5	36083.96	+0.03	19404_0½_— $55488_{1½}^{{}^{\circ}}$	
2771.005	5	20	36077.34	−0.09	*a* ^4^D_2½_— $51045_{3½}^{{}^{\circ}}$	
2771.531	5	3	36070.50	+0.02	30633_4½_— $66703_{5½}^{{}^{\circ}}$	
2771.927	2	6	36065.34	+0.04	23803_3½_— $59869_{3½}^{{}^{\circ}}$	
2772.658′	2	8	36055.83	−0.01	18000_3½_— $54056_{4½}^{{}^{\circ}}$	
2773.849	5	25	36040.35	−0.09	*a* ^4^G_2½_— $52275_{3½}^{{}^{\circ}}$	
2774.092	?	30	36037.20	+0.04	26929_5½_— $62966_{5½}^{{}^{\circ}}$	
2774.433		10?	36032.76	+0.01	*a* ^4^P_1½_— $46625_{0½}^{{}^{\circ}}$	
2774.988	6	20	36025.56	+0.02	18000_3½_— $54026_{2½}^{{}^{\circ}}$	
2775.878	1	5	36014.01	0.00	*a* ^4^G_4½_— $52567_{4½}^{{}^{\circ}}$	
2776.509	20	100	36005.84	+0.01	*a* ^4^F_4½_— $50863_{2½}^{{}^{\circ}}$	6
2777.536	2	10	35992.51	+0.07	27273_3½_— $63266_{3½}^{{}^{\circ}}$	
2777.870	8	30	35988.18	−0.05	20780_4½_— $56768_{3½}^{{}^{\circ}}$	
2778.124		2	35984.90	+0.06	29341_4½_— $65326_{5½}^{{}^{\circ}}$	
2778.281		3	35982.86	−0.01	24918_1½_— $60900_{2½}^{{}^{\circ}}$	
2778.429	5	6	35980.95	−0.17	*a* ^4^D_0½_— $49154_{0½}^{{}^{\circ}}$	
2778.694	20	80	35977.51	+0.03	*a* ^4^G_3½_— $52567_{4½}^{{}^{\circ}}$	res
2780.285	60*A*	40	35956.94	+0.05	*a* ^6^D_2½_— *z* ${\;}^{6}F_{1½}^{{}^{\circ}}$	res
2782.142	20	80	35932.93	−0.02	*a* ^4^G_5½_— $53369_{4½}^{{}^{\circ}}$	
2783.979	2	6	35909.22	−0.19	24991_1½_— $60900_{2½}^{{}^{\circ}}$	
2784.290	2	15	35905.21	−0.04	20534_5½_— $56439_{6½}^{{}^{\circ}}$	
2785.126	1	3	35894.43	−0.11	25672_2½_— $61566_{2½}^{{}^{\circ}}$	
2785.634	12	50	35887.90	0.00	19070_4½_— $54958_{5½}^{{}^{\circ}}$	7
2786.300 2786.340	$\begin{array}{l} 4\\ 3 \end{array}\}$	20	35879.31 35878.80	+0.02 −0.06	20534_5½_— $56413_{4½}^{{}^{\circ}}$ *a* ^4^F_2½_— $47179_{1½}^{{}^{\circ}}$	
2788.403		2	35852.25	+0.07	*a* ^4^G_2½_— $52087_{2½}^{{}^{\circ}}$	
2788.536	4	6	35850.54	−0.09	19637_2½_— $55488_{1½}^{{}^{\circ}}$	
2789.197	10	20	35842.04	−0.06	20534_5½_— $56376_{5½}^{{}^{\circ}}$	7
2790.160	2	18	35829.67	−0.03	28377_5½_— $64207_{4½}^{{}^{\circ}}$	
2790.433	5	50	35826.17	−0.14	23450_2½_— $59276_{3½}^{{}^{\circ}}$	res
2790.998	3	3	35818.92	−0.08	28491_1½_— $64310_{2½}^{{}^{\circ}}$	
2791.740	2	5	35809.40	−0.08	*a* ^4^D_0½_— $48982_{1½}^{{}^{\circ}}$	
2791.850	—?	1	35807.98	−0.02	*a* ^4^P_2½_— $49242_{2½}^{{}^{\circ}}$	
2793.622	1	4	35785.28	+0.06	26929_5½_— $62714_{6½}^{{}^{\circ}}$	
2796.674	1	3	35746.23	−0.11	19276_2½_— $55022_{3½}^{{}^{\circ}}$	
2796.866	3	6	35743.77	−0.15	*a* ^4^F_1½_— $44455_{0½}^{{}^{\circ}}$	res
2797.298		2	35738.25	−0.16	24918_1½_— $60656_{2½}^{{}^{\circ}}$	
2797.879		1	35730.83	−0.27	25169_1½_— $60900_{2½}^{{}^{\circ}}$	
2798.353		3	35724.78	−0.06	28631_3½_— $64356_{3½}^{{}^{\circ}}$	
2799.042	20	100	35715.98	−0.05	*a* ^4^D_3½_— $50863_{4½}^{{}^{\circ}}$	5
2799.248	1		35713.36	−0.29	18990_1½_— $54704_{2½}^{{}^{\circ}}$	
2799.312	1		35712.54	−0.02	*a* ^4^F_3½_— $49124_{3½}^{{}^{\circ}}$	
2801.058	4	30	35690.28	−0.14	*a* ^4^P_2½_— $49124_{3½}^{{}^{\circ}}$	
2801.430		10	35685.54	−0.07	*a* ^4^G_3½_— $52275_{3½}^{{}^{\circ}}$	6 res
3802.705		3	35669.31	+0.01	28118_2½_— $63788_{3½}^{{}^{\circ}}$	
2803.068		4	35664.70	−0.25	24991_1½_— $60656_{2½}^{{}^{\circ}}$	
2803.233	4	10	35662.60	−0.02	22194_3½_— $57856_{2½}^{{}^{\circ}}$	
2803.302	1	3	35661.71	+0.07	29341_2½_— $65003_{4½}^{{}^{\circ}}$	
2803.604	8	25	35657.87	−0.10	*a* ^4^D_1½_— $50292_{2½}^{{}^{\circ}}$	res
2803.682		3	35656.88	+0.01	23234_4½_— $58891_{5½}^{{}^{\circ}}$	
2803.909		5	35654.00	−0.13	25672_2½_— $61326_{3½}^{{}^{\circ}}$	
2804.922	3	12	35641.12	+0.04	23046_3½_— $58687_{4½}^{{}^{\circ}}$	
2805.177	2	15	35637.88	−0.05	30633_4½_— $66270_{4½}^{{}^{\circ}}$	6
2805.546		7	35633.20	−0.06	20780_4½_— $56413_{4½}^{{}^{\circ}}$	6
2805.936	20	120	35628.24	$\left\{ \begin{array}{l} -0.13\\ +0.05 \end{array}\right.$	20455_1½_— $56084_{1½}^{{}^{\circ}}$ *a* ^4^G_2½_— $51863_{3½}^{{}^{\circ}}$	
2806.392	3	4	35622.45	−0.07	*a* ^4^P_0½_— $44455_{0½}^{{}^{\circ}}$	
2806.626	3	10	35619.48	+0.01	24804_3½_— $60424_{3½}^{{}^{\circ}}$	6
2808.474	4	40	35596.04	−0.03	20780_4½_— $56376_{5½}^{{}^{\circ}}$	res
2808.956	3	50	35589.93	−0.02	22139_2½_— $57729_{3½}^{{}^{\circ}}$	res
2810.085		3	35575.64	0.00	28631_3½_— $64207_{4½}^{{}^{\circ}}$	
2811.743	1	6	35554.65	−0.01	29341_4½_— $64896_{3½}^{{}^{\circ}}$	
2812.210	8	50	35548.76	0.00	*a* ^4^P_2½_— $48982_{1½}^{{}^{\circ}}$	res
2813.228	3	15	35535.90	+0.06	22194_3½_— $57729_{3½}^{{}^{\circ}}$	
2814.106		2	35524.81	−0.11	19637_2½_— $55162_{2½}^{{}^{\circ}}$	
2814.798	4	30	35516.07	+0.03	19442_6½_— $54958_{5½}^{{}^{\circ}}$	5
2815.720		2	35504.44	−0.10	22503_1½_— $58007_{1½}^{{}^{\circ}}$	
2816.283	6	10	35497.35	0.00	*a* ^4^G_3½_— $52087_{2½}^{{}^{\circ}}$	
2816.478		2	35494.90	+0.26	18990_1½_— $54485_{0½}^{{}^{\circ}}$	
2817.422	1	3	35483.00	−0.11	24991_1½_— $60474_{2½}^{{}^{\circ}}$	
2819.002	4	12	35463.11	−0.02	*a* ^4^D_2½_— $50430_{1½}^{{}^{\circ}}$	7
2819.897	4	20	35451.85	+0.07	24804_3½_— $60256_{3½}^{{}^{\circ}}$	res
2820.532	3	8	35443.87	−0.07	23955_5½_— $59399_{4½}^{{}^{\circ}}$	
2821.800	5	35	35427.95	−0.14	19276_2½_— $54704_{2½}^{{}^{\circ}}$	res
2822.542	25	125	35418.64	−0.10	*a* ^4^F_3½_— $48830_{3½}^{{}^{\circ}}$	res
2824.144		4	35398.54	+0.06	28631_3½_— $64030_{2½}^{{}^{\circ}}$	
2824.306	4	4	35396.52	−0.08	*a* ^4^P_2½_— $48830_{3½}^{{}^{\circ}}$	
2824.680	2	6	35391.83	−0.03	26158_4½_— $61550_{3½}^{{}^{\circ}}$	
2824.896	1	7	35389.12	+0.02	2840l_1½_— $63880_{2½}^{{}^{\circ}}$	
2825.188	1	6	35385.47	−0.01	19637_2½_— $55022_{3½}^{{}^{\circ}}$	
2826.482	3	15	35369.27	0.00	18000_3½_— $58869_{4½}^{{}^{\circ}}$	7
2827.542		8	35356.00	+0.02	19404_0½_— $54760_{0½}^{{}^{\circ}}$	
2830.064	12	80	35324.50	−0.01	*a* ^4^D_2½_— $50292_{2½}^{{}^{\circ}}$	6
2831.236	7	35	35309.88	−0.01	*a* ^4^G_4½_— $51863_{3½}^{{}^{\circ}}$	
2831.643	1	2	35304.80	0.00	25160_1½_— $60474_{2½}^{{}^{\circ}}$	
2832.215		3	35297.67	+0.23	23450_2½_— $58747_{1½}^{{}^{\circ}}$	
2832.848		1	35289.80	−0.38	23046_3½_— $58336_{2½}^{{}^{\circ}}$	
2834.208		50	35272.85	−0.51	*a* ^4^G_3½_— $51863_{3½}^{{}^{\circ}}$	6
2835.334	8*A*	5	35258.85	−0.21	23450_2½_— $58709_{3½}^{{}^{\circ}}$	
2838.422	5	4	35220.50	−0.01	*a* ^6^D_3½_— $39936_{2½}^{{}^{\circ}}$	
2839.820	3	10	35203.15	−0.04	*a* ^4^G_2½_— $51438_{2½}^{{}^{\circ}}$	
2839.936	1	10	35201.71	−0.09	26158_4½_— $61360_{4½}^{{}^{\circ}}$	
2841.080	2	6	35187.54	+0.01	24804_3½_— $59992_{2½}^{{}^{\circ}}$	
2842.460		6	35170.46	−0.03	31100_5½_— $66270_{4½}^{{}^{\circ}}$	
2842.700	2	15	35167.50	−0.05	26158_4½_— $61326_{3½}^{{}^{\circ}}$	
2843.045	2	2	35163.22	+0.04	27273_3½_— $62437_{4½}^{{}^{\circ}}$	
2843.449	2	5	35158.23	−0.15	19070_4½_— $54229_{5½}^{{}^{\circ}}$	
2843.603	1	12	35156.32	−0.02	28631_3½_— $63788_{3½}^{{}^{\circ}}$	6
2844.426		1	35146.15	−0.09	18990_1½_— $54137_{1½}^{{}^{\circ}}$	
2844.500	2	8	35145.24	−0.07	*a* ^4^D_3½_— $50292_{2½}^{{}^{\circ}}$	res
2845.725	3	10	35130.11	−0.02	*a* ^4^G_5½_— $52567_{4½}^{{}^{\circ}}$	res
2846.357		1	35122.31	−0.25	20039_3½_— $55162_{2½}^{{}^{\circ}}$	
2847.140	3	18	35112.65	−0.17	18000_3½_— $53113_{2½}^{{}^{\circ}}$	res
2848.234	2	5	35099.16	$\left\{ \begin{array}{l} -0.14\\ -0.13 \end{array}\right.$	19276_2½_— $54375_{2½}^{{}^{\circ}}$ 26227_2½_— $61326_{3½}^{{}^{\circ}}$	
2849.678		1	35081.38	−0.04	19404_0½_— $54485_{0½}^{{}^{\circ}}$	
2850.808	35*A*	8	35067.48	+0.25	19637_2½_— $54704_{2½}^{{}^{\circ}}$	
2851.068		4	35064.28	−0.19	24804_3½_— $59869_{3½}^{{}^{\circ}}$	
2851.554	3	3	35058.30	+0.38	22194_3½_— $57252_{2½}^{{}^{\circ}}$	
2852.083	3	70	35051.80	+0.02	30633_4½_— $65684_{5½}^{{}^{\circ}}$	5
2852.460		1	35047.17	0.00	25209_4½_— $60256_{3½}^{{}^{\circ}}$	
2853.440	3	20	35035.13	−0.15	18990_1½_— $54026_{2½}^{{}^{\circ}}$	res
2853.694		4	35032.02	−0.06	20455_1½_— $55488_{1½}^{{}^{\circ}}$	
2854.719	3	4	35019.44	−0.12	*a* ^4^G_2½_— $51254_{1½}^{{}^{\circ}}$	
2855.524		25	35009.56	0.00	25209_4½_— $60218_{5½}^{{}^{\circ}}$	
2856.256		2	35000.60	−0.04	24991_1½_— $59992_{2½}^{{}^{\circ}}$	
2857.470		1	34985.72	−0.14	19070_4½_— $54056_{4½}^{{}^{\circ}}$	
2857.695	2	6	34982.97	−0.15	20039_3½_— $55022_{2½}^{{}^{\circ}}$	6
2859.484	8	30	34961.08	−0.10	*a* ^4^P_1½_— $45553_{1½}^{{}^{\circ}}$	res
2860.898		12	34943.80			
2861.058	2	6	34941.85	−0.01	*a* ^4^G_4½_—*z* ${\;}^{6}F_{5½}^{{}^{\circ}}$	
2861.21	2	10	34939.99	−0.13	23046_3½_— $57986_{4½}^{{}^{\circ}}$	
2861.518		2	34936.23	−0.11	23955_5½_— $58891_{5½}^{{}^{\circ}}$	
2864.036		2	34905.52	−0.20	23803_3½_— $58709_{3½}^{{}^{\circ}}$	
2864.395	1		34901.14	+0.10	18000_3½_— $52901_{3½}^{{}^{\circ}}$	
2864.473	2	15	34900.20	−0.10	28187_6½_— $63087_{6½}^{{}^{\circ}}$	6
2864.744	1	8	34896.90	−0.16	26158_4½_— $61055_{4½}^{{}^{\circ}}$	7
2864.858	3	10	34895.51	+0.13	22194_3½_— $57089_{4½}^{{}^{\circ}}$	
2865.603		6	34886.43	−0.05	23450_2½_— $58336_{2½}^{{}^{\circ}}$	6
2865.804	3	20	34884.00	−0.04	23803_3½_— $58687_{4½}^{{}^{\circ}}$	
2866.322	8	10	34877.68	−0.09	*a* ^6^S_2½_— $42298_{1½}^{{}^{\circ}}$	
2866.600	4	8	34874.30	−0.04	*a* ^4^F_2½_— $46175_{3½}^{{}^{\circ}}$	
2866.751	6	20	34872.46	−0.06	*a* ^4^F_3½_— $48284_{2½}^{{}^{\circ}}$	res
2866.910		1	34870.53	−0.17	28118_2½_— $62989_{2½}^{{}^{\circ}}$	
2867.409	6	15	34864.46	−0.04	*a* ^4^P_1½_— $45457_{0½}^{{}^{\circ}}$	res
2867.697	4	3	34860.96	$\left\{ \begin{array}{l} -0.23\\ +0.28 \end{array}\right.$	19637_2½_— $54798_{3½}^{{}^{\circ}}$ 19276_2½_— $54137_{1½}^{{}^{\circ}}$	
2867.934	4	20	34858.08	+0.06	20534_5½_— $55392_{4½}^{{}^{\circ}}$	
2868.736	8	80	34848.33	−0.03	*a* ^4^G_3½_— $51438_{2½}^{{}^{\circ}}$	
2870.906	18*A*	10	34822.00	−0.33	25169_1½_— $59992_{2½}^{{}^{\circ}}$	
2871.902	2	12	34809.92	+0.02	23046_3½_— $57856_{2½}^{{}^{\circ}}$	res
2873.352	2?	10	34792.35	+0.05	22139_2½_— $56932_{1½}^{{}^{\circ}}$	
2873.836	2	12	34786.50	−0.02	19442_6½_— $54229_{5½}^{{}^{\circ}}$	
2874.478		12	34778.72	−0.18	28187_6½_— $62966_{5½}^{{}^{\circ}}$	
2875.110		4	34771.08	−0.02	25045_0½_— $59816_{1½}^{{}^{\circ}}$	
2876.684	1–	2	34752.06	+0.08	25672_2½_— $60424_{1½}^{{}^{\circ}}$	
2876.934	20*A*	10	34749.01	+0.07	22503_1½_— $57252_{2½}^{{}^{\circ}}$	6
2877.827		2	34738.26	−0.18	19637_2½_— $54375_{2½}^{{}^{\circ}}$	
2878.083	10*A*	8	34735.16	+0.14	22139_2½_— $56874_{2½}^{{}^{\circ}}$	6
2878.316	2	40	34732.35	−0.13	23955_5½_— $58687_{4½}^{{}^{\circ}}$	
2880.164	1–	8	34710.07	−0.03	28377_5½_— $63087_{6½}^{{}^{\circ}}$	
2881.538		8	34693.52	+0.14	30633_4½_— $65326_{5½}^{{}^{\circ}}$	
2882.400	3	20	34683.14	+0.02	23046_3½_— $57729_{3½}^{{}^{\circ}}$	6
2883.152	3	3	34674.10	+0.13	26227_2½_— $60900_{2½}^{{}^{\circ}}$	
2883.247	6	4	34672.95	+0.11	26929_5½_— $61602_{6½}^{{}^{\circ}}$	
2883.915		2	34664.92	+0.05	20039_3½_— $54704_{2½}^{{}^{\circ}}$	
2884.310	2	20	34660.18	+0.06	26929_5½_— $61589_{5½}^{{}^{\circ}}$	6
2885.463	1	2	34646.33	−0.24	*a* ^6^D_1½_—*z* ${\;}^{6}F_{0½}^{{}^{\circ}}$	
2886.464		6	34634.31	−0.13	28631_3½_— $63266_{3½}^{{}^{\circ}}$	
2886.896	5	$\begin{array}{l} 35 \end{array}\}$	34629.13	+0.11	*a* ^6^S_2½_—*z* ${\;}^{6}F_{2½}^{{}^{\circ}}$	
2886.923			34628.81	+0.17	22139_2½_— $56768_{3½}^{{}^{\circ}}$	
2888.318	15*A*	8	34612.08	+0.09	20780_4½_— $55392_{4½}^{{}^{\circ}}$	*d*
2888.699		4	34607.52	−0.22	*a* ^6^D_1½_— $49242_{2½}^{{}^{\circ}}$	
2889.780	4	50	34594.58	−0.09	24804_3½_— $59399_{4½}^{{}^{\circ}}$	
2890.634	2	25	34584.35	+0.01	31100_5½_— $65684_{5½}^{{}^{\circ}}$	6
2891.456	3	20	34574.52	−0.01	22194_3½_— $56768_{3½}^{{}^{\circ}}$	6
2892.912	2	10	34557.12	+0.02	23450_2½_— $58007_{1½}^{{}^{\circ}}$	res
2894.924	2	10	34533.10	−0.04	23803_3½_— $58336_{2½}^{{}^{\circ}}$	
2895.441	2	10	34526.94	−0.02	28187_6½_— $62714_{6½}^{{}^{\circ}}$	6
2897.726	2	3	34499.71	−0.11	19637_2½_— $54137_{1½}^{{}^{\circ}}$	
2898.102		2	34495.24	+0.19	23234_4½_— $57729_{3½}^{{}^{\circ}}$	
2898.360		1	34492.17	+0.06	*a* ^4^G_4½_— $51045_{3½}^{{}^{\circ}}$	
2901.174	2	25	34458.71	−0.12	20039_3½_— $54498_{3½}^{{}^{\circ}}$	
2901.448		3	34455.46	−0.12	*a* ^4^G_3½_— $51045_{3½}^{{}^{\circ}}$	
2902.201	30*A*.	20	34446.52	−0.12	29341_4½_— $63788_{3½}^{{}^{\circ}}$	
2903.500		50	34431.11	−0.09	26929_5½_— $61360_{4½}^{{}^{\circ}}$	
2904.081	8	80	34424.22	−0.01	20534_5½_— $54958_{5½}^{{}^{\circ}}$	6
2904.556	2	8	34418.58	−0.08	22194_3½_— $56612_{3½}^{{}^{\circ}}$	6
2904.843	–?	5	34415.18	−0.08	*a* ^4^D_0½_— $47588_{1½}^{{}^{\circ}}$	
2905.602	8*A*	12	34406.20	0.00	23450_2½_— $57856_{2½}^{{}^{\circ}}$	6
2906.416	2	12	34396.57	+0.10	22535_0½_— $56932_{1½}^{{}^{\circ}}$	
2908.504	3	25	34371.87	−0.06	22503_1½_— $56874_{2½}^{{}^{\circ}}$	
2910.332	2	10	34350.30	−0.02	22194_3½_— $56544_{2½}^{{}^{\circ}}$	
2911.542	2	20	34336.01	$\left\{ \begin{array}{l} -0.07\\ -0.07 \end{array}\right.$	20039_3½_— $54375_{2½}^{{}^{\circ}}$ 30633_4½_— $64969_{5½}^{{}^{\circ}}$	
2912.464	2	25	34325.15			
2912.584	3	5	34323.73	−0.09	*a* ^4^F_4½_— $49181_{4½}^{{}^{\circ}}$	
2912.986	1		34319.00	−0.11	*a* ^4^P_1½_— $44911_{1½}^{{}^{\circ}}$	
2913.614		2	34311.60	+0.13	26929_5½_— $61240_{5½}^{{}^{\circ}}$	
2913.748	12	30	34310.02	+0.11	*a* ^4^G_4½_— $50863_{4½}^{{}^{\circ}}$	6
2914.272		30	34303.85	−0.28	20455_1½_— $54760_{0½}^{{}^{\circ}}$	
2914.655	2	15	34299.34	+0.05	19070_4½_— $53369_{4½}^{{}^{\circ}}$	6
2916.347		5	34279.44	+0.02	23450_2½_— $57729_{3½}^{{}^{\circ}}$	
2916.584	3	8	34276.66	−0.08	27273_3½_— $61550_{3½}^{{}^{\circ}}$	
2916.768	3	10	34274.50	$\left\{ \begin{array}{l} +0.22\\ -0.08 \end{array}\right.$	*a* ^4^D_2½_— $49242_{2½}^{{}^{\circ}}$ 18000_3½_— $52275_{3½}^{{}^{\circ}}$	
2916.896	2	12	34273.00	−0.38	*a* ^4^G_3½_— $50863_{4½}^{{}^{\circ}}$	
2917.389	3	5	34267.20	−0.10	*a* ^4^F_4½_— $49124_{3½}^{{}^{\circ}}$	
2917.540	1	5	34265.42	+0.02	26158_4½_— $60424_{3½}^{{}^{\circ}}$	
2917.895		3	34261.25	−0.25	25672_2½_— $59933_{3½}^{{}^{\circ}}$	
2918.633	25	100	34252.58	−0.04	*a* ^4^F_2½_— $45553_{1½}^{{}^{\circ}}$	7
2918.976	2	10	34248.57	−0.11	20455_1½_— $54704_{2½}^{{}^{\circ}}$	
2919.508	2	12	34242.33	−0.15	20780_4½_— $55022_{3½}^{{}^{\circ}}$	
2920.903	—	6	34225.98	+0.04	31100_5½_— $65326_{5½}^{{}^{\circ}}$	
2921.908	10*A*	12	34214.21	−0.09	28118_2½_— $62333_{2½}^{{}^{\circ}}$	*d*
2922.674	—	5	34205.24	+0.04	23046_3½_— $57252_{2½}^{{}^{\circ}}$	
2923.382	5*A*	10	34196.95	−0.03	25672_2½_— $59869_{3½}^{{}^{\circ}}$	
2923.451	1	10	34196.15	+0.04	*a* ^4^G_2½_— $50430_{1½}^{{}^{\circ}}$	
2923.980	3	12	34189.96	−0.10	25209_4½_— $59399_{4½}^{{}^{\circ}}$	6
2924.996	4	40	34178.08	−0.12	20780_4½_— $54958_{5½}^{{}^{\circ}}$	5
2925.834	5	125	34168.30	−0.04	33910_5½_— $68078_{6½}^{{}^{\circ}}$	5
2926.836	3	15	34156.60	−0.10	*a* ^4^D_2½_— $49124_{3½}^{{}^{\circ}}$	res
2926.990	40*A*	10	34154.80	+0.26	*a* ^4^P_2½_— $47588_{1½}^{{}^{\circ}}$	
2927.710	3	20	34146.40	−0.06	19276_2½_— $53422_{1½}^{{}^{\circ}}$	7
2929.984	3	20	34119.90	−0.07	26158_4½_— $60278_{1½}^{{}^{\circ}}$	6
2931.530	2	20	34101.90	−0.04	33910_5½_— $68012_{5½}^{{}^{\circ}}$	6
2931.895	5	35	34097.65	−0.06	26158_4½_— $60256_{3½}^{{}^{\circ}}$	res
2932.864	3	20	34086.39	+0.07	18000_3½_— $52087_{2½}^{{}^{\circ}}$	res
2933.068	1	1	34083.80	−0.32	28361_3½_— $62715_{4½}^{{}^{\circ}}$	
2935.201	2	6	34059.25	+0.01	28377_5½_— $62437_{4½}^{{}^{\circ}}$	
2935.358	8	25	34057.43	−0.06	*a* ^4^G_2½_— $50292_{2½}^{{}^{\circ}}$	6
2935.726	2	10	34053.16	−0.03	19276_2½_— $53329_{1½}^{{}^{\circ}}$	
2935.806	—	4	34052.23	−0.20	27273_3½_— $61326_{3½}^{{}^{\circ}}$	
2936.61	40*A*	125	34042.92	+0.26	23046_3½_— $57089_{4½}^{{}^{\circ}}$	
2937.200	8?	15	34036.07	−0.02	19404_0½_— $53440_{0½}^{{}^{\circ}}$	6
2937.61	10*A*	12	34031.33	−0.19	22395_5½_— $57986_{4½}^{{}^{\circ}}$	
2937.774	2	4	34029.42	−0.03	26227_2½_— $60856_{3½}^{{}^{\circ}}$	
2938.685	?	4	34018.87	−0.03	19404_0½_— $53422_{1½}^{{}^{\circ}}$	
2938.872	2	12	34016.71	−0.09	20039_3½_— $54056_{4½}^{{}^{\circ}}$	
2939.757	6	35	34006.47	−0.09	*a* ^4^D_0½_— $47179_{1½}^{{}^{\circ}}$	
2940.204	8	60	34001.30	−0.07	*a* ^4^F_3½_— $47413_{2½}^{{}^{\circ}}$	
2941.476	4	5	33986.60	+0.10	20039_3½_— $54026_{2½}^{{}^{\circ}}$	
2942.128	12*A*	10	33979.07	−0.16	*a* ^4^P_2½_— $47413_{2½}^{{}^{\circ}}$	
2942.263	2	10	33977.49	−0.01	*a* ^4^D_3½_— $49124_{3½}^{{}^{\circ}}$	
2942.618	3	10	33973.41	−0.07	*a* ^4^F_4½_— $48830_{3½}^{{}^{\circ}}$	
2945.137	–	6	33944.35	+0.02	22139_2½_— $56084_{1½}^{{}^{\circ}}$	
2946.454	–	25	33929.18			
2947.136	–?	2	33921.33	−0.15	34091_4½_— $68012_{5½}^{{}^{\circ}}$	
2950.453	4	40	33883.20	−0.01	24804_3½_— $58687_{4½}^{{}^{\circ}}$	res
2952.262	75*A*	100	33862.44	$\left\{ \begin{array}{l} -0.22\\ +0.11 \end{array}\right.$	*a* ^4^P_1½_— $44455_{0½}^{{}^{\circ}}$ 18000_3½_— $51863_{3½}^{{}^{\circ}}$	*d*
2952.974	*4A*	4	33854.27	−0.32	23234_4½_— $57089_{4½}^{{}^{\circ}}$	
2954.070	*4A*	3	33841.71	−0.49	28491_1½_— $62333_{2½}^{{}^{\circ}}$	
2954.482	4	15	33836.99	−0.01	19276_2½_— $53113_{2½}^{{}^{\circ}}$	6 res
2955.007	8	15	33830.98	−0.08	19070_4½_— $52901_{3½}^{{}^{\circ}}$	
2955.256	2	10	33828.13	−0.06	23046_3½_— $56874_{2½}^{{}^{\circ}}$	
2956.67	20*A*	10	33811.96	−0.08	18990_1½_— $52803_{1½}^{{}^{\circ}}$	
2957.266	1	12	33805.12	−0.06	28631_3½_— $62437_{1½}^{{}^{\circ}}$	
2957.590	1		33801.43	−0.07	23450_2½_— $57252_{2½}^{{}^{\circ}}$	
2958.972		4	33785.65	+0.05	19637_2½_— $53422_{1½}^{{}^{\circ}}$	
2959.312	–	2	33781.77	−0.17	27273_3½_— $61055_{4½}^{{}^{\circ}}$	
2959.910	–	10	33774.94	+0.02	26158_4½_— $59933_{3½}^{{}^{\circ}}$	
2960.771	–	1	33765.12	−0.08	26227_2½_— $59992_{2½}^{{}^{\circ}}$	
2961.020	12	50	33762.28	−0.02	*a* ^4^P_1½_— $44354_{2½}^{{}^{\circ}}$	
2961.558	–	2	33756.15	−0.23	24991_1½_— $58747_{1½}^{{}^{\circ}}$	
2964.412	–	1	33723.65	−0.03	30633_4½_− $64356_{3½}^{{}^{\circ}}$	
2964.898	2	20	33718.12	−0.07	20780_4½_— $54498_{3½}^{{}^{\circ}}$	res
2965.589	2	15	33710.27	−0.13	26158_4½_— $59869_{3½}^{{}^{\circ}}$	
2966.417	4*A*	2	33700.86	+0.17	19637_2__½_— $53338_{3½}^{{}^{\circ}}$	
2967.926	4	20	33683.73	+0.05	*a* ^4^D_3½_— $48830_{3½}^{{}^{\circ}}$	6
2970.908	2	5	33649.92	−0.20	*a* ^4^D_1½_— $48284_{2½}^{{}^{\circ}}$	
2973.092	–	3	33625.24	+0.02	19276_2½_— $52901_{3½}^{{}^{\circ}}$	
2974.377	20	35	33610.67	+0.12	*a* ^4^F_2½_— $44911_{1½}^{{}^{\circ}}$	res
2975.078	3	10	33602.75	−0.01	18990_1½_— $52593_{0½}^{{}^{\circ}}$	
2976.476	15	50	33586.97	+0.03	*a* ^4^F_1½_— $42298_{1½}^{{}^{\circ}}$	res
2976.985	–	2	33581.23	−0.01	22503_1½_— $56084_{1½}^{{}^{\circ}}$	
2977.442		3	33576.08	−0.02	*a* ^4^F_2½_— *z* ${\;}^{6}F_{3½}^{{}^{\circ}}$	
2977.580	4	40	33574.52	+0.04	30633_4½_— $64207_{4½}^{{}^{\circ}}$	6
2977.950	3	20	33570.35	+0.04	20445_1½_— $54026_{2½}^{{}^{\circ}}$	
2978.332	1	6	33566.05	+0.11	23046_3½_— $56612_{3½}^{{}^{\circ}}$	
2981.319	1		33532.41	+0.10	24804_3½_— $58336_{2½}^{{}^{\circ}}$	
2982.219	2	25	33522.29	+0.10	20534_5½_— $54056_{4½}^{{}^{\circ}}$	
2984.426		6	33497.50	−0.10	23046_3__½_— $56544_{2½}^{{}^{\circ}}$	
2985.830		6	33481.75	−0.02	23450_2½_— $56932_{1½}^{{}^{\circ}}$	
2986.103	5	25	33478.69	+0.09	25209_4½_— $58687_{4½}^{{}^{\circ}}$	6
2986.382	3	20	33475.57	+0.06	*a* ^4^F_4½_— $48332_{5½}^{{}^{\circ}}$	res
2987.294	15	60	33465.35	−0.19	*a* ^4^P_0½_— $42298_{1½}^{{}^{\circ}}$	res
2988.502	4	12	33451.82	−0.07	*a* ^4^D_0½_— $46625_{0½}^{{}^{\circ}}$	res
2988.771		12	33448.81	+ 0.13	20780_4½_— $54229_{5½}^{{}^{\circ}}$	
2990.850		12	33425.56	−0.47	*a* ^4^G_5½_— $50863_{4½}^{{}^{\circ}}$	
2991.470		4	33418.63	−0.25	24918_1½_— $58336_{2½}^{{}^{\circ}}$	
2991.855		3	33414.33	−0.25	28187_6½_— $61602_{6½}^{{}^{\circ}}$	
2992.043		18	33412.23			
2994.700	3	60	33382.59	−0.06	27273_3½_— $60656_{2½}^{{}^{\circ}}$	
2995.129		6	33377.81	−0.06	23234_4½_— $56612_{3½}^{{}^{\circ}}$	
2997.685		8	33349.35	−0.02	26929_5½_— $60278_{4½}^{{}^{\circ}}$	
2998.050		5	33345.29	−0.13	24991_1½_*—* $58336_{2½}^{{}^{\circ}}$	
2998.693	15	50	33338.14	−0.05	*a* ^4^F_1½_—*z* ${\;}^{6}F_{2½}^{{}^{\circ}}$	res
3000.499		3	33318.07	−0.04	23450_2½_— $56768_{3½}^{{}^{\circ}}$	
3000.624	5	50	33316.69	+ 0.03	*a* ^4^D_2½_— $48248_{2½}^{{}^{\circ}}$	res
3002.287	2	40	33298.23	−0.10	20039_3½_— $53338_{3½}^{{}^{\circ}}$	6 res
3003.070	4	10	33289.55	+ 0.05	26929_5½_*—* $60218_{5½}^{{}^{\circ}}$	6
3003.435		15	33285.51	−0.11	23803_3½_*—* $57089_{4½}^{{}^{\circ}}$	
3004.284		5	33276.10	−0.06	20780_4½_*—* $54056_{4½}^{{}^{\circ}}$	
3007.511		4?	33240.40	−0.20	26158_4½_— $59399_{4½}^{{}^{\circ}}$	
3008.946	2	25	33224.55	+ 0.17	28377_5½_— $61602_{6½}^{{}^{\circ}}$	7
3010.131		3	33211.47	−0.19	28377_5½_— $61589_{5½}^{{}^{\circ}}$	
3010.750	4	80	33204.64	+ 0.04	19070_4½_*—* $52275_{3½}^{{}^{\circ}}$	res
3011.102		2	33200.76	− 0.05	27273_3½_*—* $60474_{2½}^{{}^{\circ}}$	
3011.327		4	33198.27	−0.02	22194_3½_*—* $55392_{4½}^{{}^{\circ}}$	
3012.102	2	15	33189.74	+ 0.10	19404_0½_— $52593_{0½}^{{}^{\circ}}$	res
3013.093		1	33178.82	+ 0.05	23234_4½_— $56413_{4½}^{{}^{\circ}}$	
3014.200	6*A*	3	33166.63	−0.48	25169_1½_*—* $58336_{2½}^{{}^{\circ}}$	
3014.606	2	25	33162.17	− 0.07	23450_2½_— $56612_{3½}^{{}^{\circ}}$	res
3015.694	1	8	33150.20	−0.08	27273_3½_— $60424_{3½}^{{}^{\circ}}$	6
3017.148	2	25	33134.23	+ 0.17	23955_5½_— $57089_{4½}^{{}^{\circ}}$	
3018.624	1	20	33118.03	−0.04	26158_4½_— $59276_{3½}^{{}^{\circ}}$	5
3019.628		3	33107.08	+ 0.04	31100_5½_*—* $64207_{4½}^{{}^{\circ}}$	
3020.630	4	20	33096.04	−0.02	18990_1½_*—* $52087_{2½}^{{}^{\circ}}$	
3021.982	20	80	33081.23	−0.24	*a* ^4^F_3½_*—z* ${\;}^{6}F_{4½}^{{}^{\circ}}$	
3022.482		20	33075.76	$\{\;\begin{array}{c} -0.02\\ +0.06 \end{array}$	25672_2½_— $58747_{1½}^{{}^{\circ}}$ 28491_1½_— $61566_{2½}^{{}^{\circ}}$	
3022.672	3	30	33073.68	−0.10	20039_3½_— $53113_{2½}^{{}^{\circ}}$	
3022.906	2	6	33071.12	−0.03	23803_3½_— $56874_{2½}^{{}^{\circ}}$	
3024.502	25	250	33053.67	−0.07	*a* ^4^F_2½_— $44354_{2½}^{{}^{\circ}}$	res
3025.993		1	33037.38	−0.02	25672_2½_— $58709_{3½}^{{}^{\circ}}$	
3027.369		6	33022.33	0.00	22139_2½_— $55162_{2½}^{{}^{\circ}}$	
3028.750		10	33007.31	+ 0.05	*a* ^4^G_2½_— $49242_{2½}^{{}^{\circ}}$	
3028.975		8	33004.86	+ 0.01	27273_3½_— $60278_{4½}^{{}^{\circ}}$	
3029.524		4	32998.88	+ 0.12	19276_2½_*—* $52275_{3½}^{{}^{\circ}}$	
3030.793		2	32985.06	+ 0.11	22503_1½_*—* $55488_{1½}^{{}^{\circ}}$	
3031.000		25	32982.81	+ 0.07	28377_5½_— $61360_{4½}^{{}^{\circ}}$	
3032.450	3	15	32967.04	−0.01	20455_1½_— $53422_{1½}^{{}^{\circ}}$	res
3032.660		8	32964.76	−0.01	23803_3½_*—* $56768_{3½}^{{}^{\circ}}$	
3033.626		30	32954.26	−0.02	*a* ^4^D_1½_— $47288_{1½}^{{}^{\circ}}$	res
3033.790		2	32952.48	+ 0.27	22535_0½_— $55488_{1½}^{{}^{\circ}}$	
3033.913	4	20	32951.14	+ 0.11	19404_0½_— $52355_{0½}^{{}^{\circ}}$	
3036.311		1	32925.11	−0.14	24804_3½_*—* $57729_{3½}^{{}^{\circ}}$	
3036.670	5	50	32921.23	−0.07	*a* ^4^P_2½_*—* $46355_{2½}^{{}^{\circ}}$	
3039.578	3	20	32889.73	+ 0.05	*a* ^4^G_2½_— $49124_{3½}^{{}^{\circ}}$	res
3044.400		10	32837.64	− 0.09	25169_1½_*—* $58007_{1½}^{{}^{\circ}}$	6
3044.564		1	32835.88	+ 0.26	20534_5½_— $53369_{4½}^{{}^{\circ}}$	
3045.216		15	32828.85	+ 0.07	22194_3½_*—* $55022_{3½}^{{}^{\circ}}$	
3046.911	1	5	32810.57	+ 0.07	19276_2½_*—* $52087_{2½}^{{}^{\circ}}$	
3047.060	1	15	32808.97	+ 0.07	23803_3½_— $56612_{3½}^{{}^{\circ}}$	6
3048.604	4	40	32792.36	+ 0.01	19070_4½_— $51863_{3½}^{{}^{\circ}}$	res
3049.563	–?	3	32782.03	−0.04	28118_2½_*—* $60900_{2½}^{{}^{\circ}}$	
3049.850	50	50	32778.96	−0.01	*a* ^4^D_1½_— $47413_{2½}^{{}^{\circ}}$	
3051.29	25	250	32763.49	+ 0.03	*a* ^4^F_3½_— $46175_{3½}^{{}^{\circ}}$	res
3053.354	6	$\begin{array}{c} ?\\ 20 \end{array}\}$	32741.34	+ 0.02	*a* ^4^P_2½_*—* $46175_{3½}^{{}^{\circ}}$	
3053.420			32740.64	+ 0.08	23803_3½_— $56544_{2½}^{{}^{\circ}}$	
3054.129		1	32733.03	+ 0.03	26158_4½_*—* $58891_{5½}^{{}^{\circ}}$	
3058.452	1	20	32686.79	−0.04	25169_1½_*—* $57856_{2½}^{{}^{\circ}}$	res
3059.268		3	32678.06	+ 0.06	28377_5½_— $61055_{4½}^{{}^{\circ}}$	
3061.027		10	32659.28	+ 0.04	22503_1½_*—* $55162_{2½}^{{}^{\circ}}$	
3061.678	6	15	32652.35	−0.08	*a* ^4^G_3½_— $49242_{2½}^{{}^{\circ}}$	res
3063.422		8	32633.74	−0.06	23450_2½_— $56084_{1½}^{{}^{\circ}}$	
3063.972	4	30	32627.89	−0.01	*a* ^4^G_4½_— $49181_{4½}^{{}^{\circ}}$	res
3064.635		3	32620.83	+ 0.01	*a* ^4^D_2½_— $47588_{1½}^{{}^{\circ}}$	
3066.976	6	60	32595.93	+ 0.07	*a* ^4^G_2½_*—* $48830_{3½}^{{}^{\circ}}$	7
3067.410	3	6	32591.32	−0.05	*a* ^4^G_3½_— $49181_{4½}^{{}^{\circ}}$	
3067.572	5	50	32589.61	+ 0.02	20780_4½_— $53369_{4½}^{{}^{\circ}}$	6
3067.856	6	60	32586.59	+ 0.08	19276_2½_— $51863_{3½}^{{}^{\circ}}$	7
3069.288	8	80	32571.38	0.00	*a* ^4^G_4½_— $49124_{3½}^{{}^{\circ}}$	res
3071.234	1	15	32550.74	−0.08	26158_4½_— $58709_{3½}^{{}^{\circ}}$	res
3071.730	5	50	32545.48	−0.10	*a* ^4^D_1½_— $47179_{1½}^{{}^{\circ}}$	res
3072.740	4	30	32534.78	−0.07	*a* ^4^G_3½_— $49124_{3½}^{{}^{\circ}}$	res
3073.403		4	32527.77	+ 0.36	20039_3½_— $52567_{4½}^{{}^{\circ}}$	
3075.963		8	32500.70			
3077.520	30	300	32484.26	+ 0.06	23955_5½_— $56439_{6½}^{{}^{\circ}}$	5
3078.868	?	15	32470.03	+ 0.03	26929_5½_— $59399_{4½}^{{}^{\circ}}$	res
3079.992		20	32458.19	−0.05	23955_5½_— $56413_{4½}^{{}^{\circ}}$	4
3080.787		2	32449.81	+ 0.17	19637_2½_— $52087_{2½}^{{}^{\circ}}$	
3081.042	4	40	32447.13	+ 0.06	18990_1½_— $51438_{2½}^{{}^{\circ}}$	res
3081.215	1	3	32445.30	−0.21	*a* ^4^D_2½_— $47413_{2½}^{{}^{\circ}}$	
3083.520	2	20	32421.05	0.00	23955_5½_— $56376_{5½}^{{}^{\circ}}$	6
3087.394	10	50	32380.37	+ 0.05	*a* ^4^D_0½_— $43553_{1½}^{{}^{\circ}}$	res
3089.742		6	32355.77	0.00	28118_2½_— $60474_{2½}^{{}^{\circ}}$	
3090.712	2	2	32345.62	+ 0.07	23046_3½_— $55392_{4½}^{{}^{\circ}}$	
3091.870		3	32333.49	+ 0.01	30633_4½_— $62966_{5½}^{{}^{\circ}}$	
3094.629		4	32304.67	+ 0.18	22194_3½_— $54498_{3½}^{{}^{\circ}}$	
3095.870	5	50	32291.73	+ 0.10	18000_3½_— $50292_{2½}^{{}^{\circ}}$	res
3098.031		2	32269.20	+ 0.09	28631_3½_— $60900_{2½}^{{}^{\circ}}$	
3098.300	2	12	32266.40	+ 0.09	*a* ^4^D_3½_— $47413_{2½}^{{}^{\circ}}$	
3098.580	–	12	32263.48	+ 0.04	18990_1½_— $51254_{1½}^{{}^{\circ}}$	
3098.870	–	8	32260.47	+ 0.03	24991_1½_— $57252_{2½}^{{}^{\circ}}$	
3100.074		5	32247.93	+ 0.03	29341_4½_— $61589_{5½}^{{}^{\circ}}$	
3100.736	5	50	32241.05	+ 0.02	*a* ^4^G_3½_— $48830_{3½}^{{}^{\circ}}$	res
3101.224		4	32235.98	+ 0.13	22139_2½_— $54375_{2½}^{{}^{\circ}}$	
3102.204	5	50	32225.79	+0.14	19637_2½_— $51863_{3½}^{{}^{\circ}}$	res
3103.517	2	12	32212.16	+ 0.04	*a* ^4^D_2½_— $47179_{1½}^{{}^{\circ}}$	
3104.534		3	32201.61	+0.06	22503_1½_— $54704_{2½}^{{}^{\circ}}$	
3106.180		8	32184.55	+ 0.01	25672_2½_— $57856_{2½}^{{}^{\circ}}$	
3106.632		20	32179.86	−0.07	34091_4½_— $66270_{4½}^{{}^{\circ}}$	
3108.386		3	32161.71	+0.20	19404_2½_— $51438_{2½}^{{}^{\circ}}$	6
3108.778	6	60	32157.65	+ 0.15	23234_4½_— $55392_{4½}^{{}^{\circ}}$	res
3110.690	4	40	32137.89	+0.10	20455_1½_— $52593_{0½}^{{}^{\circ}}$	res
3111.887	1	4	32125.52	+ 0.04	27273_3½_— $59399_{4½}^{{}^{\circ}}$	
3112.463	1	4	32119.58	−0.02	*a* ^4^P_2½_— $45553_{1½}^{{}^{\circ}}$	
3112.860	20	50	32115.48	−0.02	23046_3½_— $55162_{2½}^{{}^{\circ}}$	res
3113.384		12	32110.08	+0.10	26227_2½_— $58337_{2½}^{{}^{\circ}}$	6
3116.100		3	32082.09	−0.04	25169_1½_— $57252_{2½}^{{}^{\circ}}$	
3117.263		1	32070.12	−0.20	24804_3½_— $56874_{2½}^{{}^{\circ}}$	
3118.475		5	32057.66	−0.10	25672_2½_— $57729_{3½}^{{}^{\circ}}$	
3118.981		1	32052.46	0.00	19442_6½_*—z* ${\;}^{6}F_{5½}^{{}^{\circ}}$	
3119.237	5	12	32049.83	+ 0.19	*a* ^4^G_2½_— $48284_{2½}^{{}^{\circ}}$	
3121.032		10	32031.40	+ 0.16	28187_6½_— $60218_{5½}^{{}^{\circ}}$	
3125.700	1	2	31983.59	−0.08	2849_1½_— $60474_{2½}^{{}^{\circ}}$	
3125.790	1	8	31982.64	+ 0.10	22503_1½_— $54485_{0½}^{{}^{\circ}}$	
3126.420	4	40	31976.20	+ 0.14	23046_3½_— $55022_{3½}^{{}^{\circ}}$	6 res
3127.750	1	50	31962.60	+ 0.20	26929_5½_— $58891_{5½}^{{}^{\circ}}$	6 res
3128.982	2	40	31950.01	+ 0.21	22535_0½_— $54485_{0½}^{{}^{\circ}}$	res
3129.682	1	25	31942.87			
3129.907	3	2	31940.57	−0.14	24991_1½_— $56932_{1½}^{{}^{\circ}}$	
3133.780		3	31901.10	+0.19	28377_5½_— $60278_{4½}^{{}^{\circ}}$	
3133.965		20	31899.22	$\{\begin{array}{c} -0.03\\ +0.04 \end{array}$	29341_4½_— $61240_{5½}^{{}^{\circ}}$ 20455_1½_— $52355_{0½}^{{}^{\circ}}$	
3135.156		4	31887.10	+0.03	25045_0½_— $56932_{1½}^{{}^{\circ}}$	
3135.238		4	31886.27	0.00	22139_2½_— $54026_{2½}^{{}^{\circ}}$	
3135.838	1	12	31880.17	−0.01	25209_4½_— $57089_{4½}^{{}^{\circ}}$	6
3137.224	4	8	31866.08	+ 0.04	31100_5½_— $62966_{5½}^{{}^{\circ}}$	
3137.57		2	31862.56	+ 0.10	22194_3½_— $54056_{4½}^{{}^{\circ}}$	
3138.770		6	31850.39	+ 0.07	19404_0½_— $51254_{1½}^{{}^{\circ}}$	res
3139.517	1	12	31842.81	0.00	28631_3½_— $60474_{2½}^{{}^{\circ}}$	
3140.577	2	2	31832.06	−0.10	22194_3½_— $54026_{2½}^{{}^{\circ}}$	
3140.96		2	31828.18	0.00	20158_4½_— $57986_{4½}^{{}^{\circ}}$	
3142.928		3	31808.25	+ 0.18	24804_3½_— $56612_{3½}^{{}^{\circ}}$	
3143.348	4	20	31804.00	−0.02	30633_4½_— $62437_{4½}^{{}^{\circ}}$	6
3143.670		4	31800.74	+ 0.09	19637_2½_— $51438_{2½}^{{}^{\circ}}$	res
3144.493		25	31792.42	$\{\begin{array}{c} +0.05\\ +0.14 \end{array}$	19070_4½_— $50863_{4½}^{{}^{\circ}}$ 28631_3½_— $60424_{3½}^{{}^{\circ}}$	
3145.768	20	200	31779.53	— 0.06	*a* ^4^G_4½_— $43332_{5½}^{{}^{\circ}}$	5
3146.292	?	20	31774.24	0.00	33910_5½_— $65684_{5½}^{{}^{\circ}}$	7
3146.836	2	4	31768.75	+ 0.02	19276_2½_— $51045_{3½}^{{}^{\circ}}$	
3147.474	–	3	31762.31	−0.09	25169_1½_— $56932_{1½}^{{}^{\circ}}$	
3147.825		15	31758.77	+ 0.23	26929_5½_— $58687_{4½}^{{}^{\circ}}$	
3148.678	1	10	31750.17	−0.07	28118_2½_— $59869_{3½}^{{}^{\circ}}$	res
3149.856	30	300	31738.29	+ 0.04	*a* ^4^D_0½_— $44611_{1½}^{{}^{\circ}}$	res
3151.304	5	125	31723.71	0.00	23234_4½_— $54958_{5½}^{{}^{\circ}}$	5
3151.568	5	20	31721.05	+ 0.01	*a* ^4^D_1½_— $46355_{2½}^{{}^{\circ}}$	
3152.238		4	31714.31	+ 0.07	29341_4½_— $61055_{4½}^{{}^{\circ}}$	
3152.484	25	100	31711.84	+ 0.04	23450_2½_— $55162_{2½}^{{}^{\circ}}$	6
3152.749	5	6	31709.17	+ 0.19	*a* ^6^S_2½_—*z* ${\;}^{6}F_{1½}^{{}^{\circ}}$	
3153.134		2	31705.30	+ 0.18	25169_1½_— $56874_{2½}^{{}^{\circ}}$	
3153.934		2	31697.28	−0.12	28118_2½_— $59816_{1½}^{{}^{\circ}}$	
3154.172	5	10	31694.87	+ 0.06	*a* ^4^G_3½_— $43284_{2½}^{{}^{\circ}}$	
3158.925	1	2	31647.18	+ 0.33	28631_3½_— $60278_{4½}^{{}^{\circ}}$	
3160.026	20	200	31636.15	−0.06	*a* ^4^F_4½_—*z* ${\;}^{6}F_{4½}^{{}^{\circ}}$	6
3160.673	1	4	31629.68	−0.02	26227_2½_— $57856_{2½}^{{}^{\circ}}$	6
3161.942		10	31616.98	−0.04	19637_2½_— $51254_{1½}^{{}^{\circ}}$	res
3162.225		15	31614.15	+ 0.05	31100_5½_— $62714_{6½}^{{}^{\circ}}$	
3164.262		10	31593.80	+ 0.02	34091_4½_— $65684_{5½}^{{}^{\circ}}$	
3164.794	1	30	31588.49	−0.04	23803_3½_— $55392_{4½}^{{}^{\circ}}$	
3165.657		4	31579.88	+ 0.04	25672_2½_— $57252_{2½}^{{}^{\circ}}$	
3166.534		12	31571.13	−0.05	26158_4½_— $57729_{3½}^{{}^{\circ}}$	
3167.736	1	6	31559.16	−0.17	25209_4½_— $56768_{3½}^{{}^{\circ}}$	
3168.372		2	31552.82	−0.02	24991_1½_— $56544_{2½}^{{}^{\circ}}$	
3171.355	1	6	31523.14	−0.04	22503_1½_— $54026_{2½}^{{}^{\circ}}$	
3173.564		8	31501.20	0.00	28491_1½_— $59992_{2½}^{{}^{\circ}}$	7
3174.221		1	31494.68	−0.22	20780_4½_— $52275_{3½}^{{}^{\circ}}$	
3175.958	20	200	31477.46	−0.07	*a* ^4^P_2½_— $44911_{1½}^{{}^{\circ}}$	res
3177.200	30	120	31465.15	−0.07	*a* ^4^F_3½_—*z* ${\;}^{6}F_{3½}^{{}^{\circ}}$	6
3178.036	6	60	31456.88	−0.05	*a* ^4^P_1½_—*z* ${\;}^{6}F_{2½}^{{}^{\circ}}$	res
3178.672		8	31450.58			
3179.436	15	120	31443.02	−0.06	*a* ^4^P_2½_—*z* ${\;}^{6}F_{3½}^{{}^{\circ}}$	res
3180.060	2	60	31436.85	−0.12	23955_5½_— $55392_{4½}^{{}^{\circ}}$	res
3182.194		30	31415.77	−0.07	33910_5½_— $65326_{5½}^{{}^{\circ}}$	
3183.000		2	31407.82	−0.05	19637_2½_— $51045_{3½}^{{}^{\circ}}$	
3183.441		12	31403.47	+ 0.01	25209_4½_— $56612_{3½}^{{}^{\circ}}$	
3183.965	3	10	31398.30	+ 0.01	20039_3½_— $51488_{2½}^{{}^{\circ}}$	
3185.057	2	20	31387.54	−0.04	*a* ^4^D_2½_— $46355_{2½}^{{}^{\circ}}$	6
3186.396	2	15	31374.35	−0.18	25169_1½_— $56544_{2½}^{{}^{\circ}}$	7
3187.116	1	80	31367.26			
3188.016		6	31358.40	−0.06	23803_3½_— $55162_{2½}^{{}^{\circ}}$	
3188.495	1	10	31353.69	−0.11	*a* ^4^G_2½_— $47588_{1½}^{{}^{\circ}}$	
3189.238	50*A*	100	31346.39	$\{\begin{array}{c} -0.02\\ +0.25 \end{array}$	*a* ^4^D_3½_—*z* ${\;}^{6}F_{4½}^{{}^{\circ}}$ *a* ^4^F_3½_— $44758_{4½}^{{}^{\circ}}$	
3191.016	2	20	31328.92	−0.10	23046_3½_— $54375_{2½}^{{}^{\circ}}$	res
3192.117		8	31318.12	−0.08	*a* ^4^F_4½_— $46175_{2½}^{{}^{\circ}}$	
3193.825		25	31301.37	0.00	18990_1½_— $50292_{2½}^{{}^{\circ}}$	
3195.708	2	5	31282.93	−0.08	22139_2½_— $53422_{1½}^{{}^{\circ}}$	
3200.380		6	31237.26	−0.02	28631_3½_— $59869_{2½}^{{}^{\circ}}$	
3200.565		6	31235.46	+ 0.08	34091_4½_— $65326_{5½}^{{}^{\circ}}$	
3201.583	20	60	31225.53	−0.02	*a* ^4^F_1½_— $39936_{2½}^{{}^{\circ}}$	res
3202.254		10	31218.98	−0.04	23803_3½_— $55022_{3½}^{{}^{\circ}}$	6
3203.343	12	60	31208.37	−0.01	*a* ^4^D_3½_— $46355_{2½}^{{}^{\circ}}$	res
3203.433	6	30	31207.49	−0.11	*a* ^4^D_2½_— $46175_{3½}^{{}^{\circ}}$	
3203.752		10	31204.39	+ 0.03	25209_4½_— $56413_{4½}^{{}^{\circ}}$	
3203.909	2		31202.86	+ 0.03	25672_2½_— $56874_{2½}^{{}^{\circ}}$	
3204.400	2	30	31198.07	−0.03	22139_2½_— $53338_{3½}^{{}^{\circ}}$	
3205.262		5	31189.69	−0.05	22139_2½_— $53329_{1½}^{{}^{\circ}}$	res
3206.420	6	60	31178.42	−0.07	*a* ^4^G_2½_— $47413_{2½}^{{}^{\circ}}$	res
3206.680	–?	12	31175.89	0.00	22194_3½_— $53369_{4½}^{{}^{\circ}}$	
3207.580		8	31167.15	−0.02	25209_4½_— $56376_{5½}^{{}^{\circ}}$	
3207.671		6	31166.26	+ 0.06	24918_1½_— $56084_{1½}^{{}^{\circ}}$	
3209.968	4	80	31143.96	−0.03	22194_3½_— $53338_{3½}^{{}^{\circ}}$	res
3215.275	2	40	31092.56	$\{\begin{array}{c} -0.08\\ +0.18 \end{array}$	33910_5½_— $65003_{4½}^{{}^{\circ}}$ 24991_1½_— $56084_{1½}^{{}^{\circ}}$	
3215.648	?	30	31088.95	−0.24	*a* ^4^F_2½_— $42390_{3½}^{{}^{\circ}}$	
3216.308	2	30	31082.57	$\{\begin{array}{c} -0.08\\ -0.01 \end{array}$	20780_4½_— $51863_{3½}^{{}^{\circ}}$ 29341_4½_— $60424_{3½}^{{}^{\circ}}$	
3218.806		6	31058.45	−0.09	33910_5½_— $64969_{5½}^{{}^{\circ}}$	
3219.890		5	31048.00	−0.07	23450_2½_— $54498_{3½}^{{}^{\circ}}$	
3221.914	60*A*	20	31028.49	+ 0.09	*a* ^4^D_3½_— $46175_{3½}^{{}^{\circ}}$	
3222.274		3	31025.03	+ 0.03	26227_2½_— $57252_{2½}^{{}^{\circ}}$	
3222.646		20	31021.45	− 0.09	28377_5½_— $59899_{4½}^{{}^{\circ}}$	
3223.22		2	31015.90	+ 0.09	19276_2½_— $50292_{2½}^{{}^{\circ}}$	
3223.900		8	31009.38	−0.36	23046_3½_— $54056_{4½}^{{}^{\circ}}$	
3224.320	3	4	31005.34	−0.17	20039_3½_— $51045_{3½}^{{}^{\circ}}$	
3224.550	6	15	31003.13	−0.05	23955_5½_— $54958_{5½}^{{}^{\circ}}$	6
3225.170	1	2	30997.17	+ 0.05	*a* ^4^F_2½_— $42298_{1½}^{{}^{\circ}}$	
3225.480	2	20	30994.19	0.00	23234_4½_— $54229_{5½}^{{}^{\circ}}$	res
3226.750		5	30981.99	−0.11	20455_1½_— $51438_{2½}^{{}^{\circ}}$	
3227.020	1	6	30979.40	−0.04	23046_3½_— $54026_{2½}^{{}^{\circ}}$	
3228.990	3	15	30960.50	−0.15	20534_5½_—*z* ${\;}^{6}F_{5½}^{{}^{\circ}}$	res
3229.422		8	30956.36	−0.08	30633_4½_— $61589_{5½}^{{}^{\circ}}$	
3230.604	4	20	30945.03	−0.07	*a* ^4^G_2½_— $47179_{1½}^{{}^{\circ}}$	
3230.846	8	12	30942.71	−0.15	*a*^4^F_3½_— $44354_{2½}^{{}^{\circ}}$	res
3231.053		4	30940.73	+0.15	25672_2½_— $56612_{3½}^{{}^{\circ}}$	
3231.442		1	30937.01	−0.10	22503_1½_— $53440_{0½}^{{}^{\circ}}$	
3232.132	1*5A*	6	30930.40	−0.32	26158_4½_— $57089_{4½}^{{}^{\circ}}$	
3233.140	3	8	30920.76	+0.04	*a*^4^P_2½_— $44354_{2½}^{{}^{\circ}}$	
3233.286		20	30919.36	$\{\begin{array}{c} -0.08\\ +0.02 \end{array}$	22194_3½_— $53113_{2½}^{{}^{\circ}}$ a^4^D_1½_— $45553_{1½}^{{}^{\circ}}$	
3233.46		1	30917.70	+0.12	30633_4½_— $61550_{3½}^{{}^{\circ}}$	
3233.820		2	30914.25	−0.18	25169_1½_— $56084_{1½}^{{}^{\circ}}$	
3234.50		4	30904.40	+0.03	22535_0½_— $53440_{0½}^{{}^{\circ}}$	7
3235.774	1–	8	30895.59	−0.12	*a*^4^G_5½_— $48332_{5½}^{{}^{\circ}}$	6
3236.644	3	25	30887.29	+0.11	22535_0__½_— $53422_{1½}^{{}^{\circ}}$	
3236.744		6	30886.33			
3237.684	1	30	30877.37	+0.09	29341_4½_— $60218_{5½}^{{}^{\circ}}$	
3238.220		2	30872.25	+0.01	25672_2½_— $56544_{2½}^{{}^{\circ}}$	
3243.008	5	40	30826.67	+0.02	22503_1½_— $53329_{1½}^{{}^{\circ}}$	res
3243.334	15	100	30823.58	−0.08	*a*^4^G_3½_— $47413_{2½}^{{}^{\circ}}$	5
3245.273		6	30805.16	−0.04	34091_4½_— $64896_{3½}^{{}^{\circ}}$	
3245.937		2	30798.86	+0.39	20455_1½_— $51254_{1½}^{{}^{\circ}}$	
3246.413		1	30794.35	+0.44	22535_0½_— $53329_{1½}^{{}^{\circ}}$	
3246.498	1	12	30793.54	−0.03	19637_2½_— $50430_{1½}^{{}^{\circ}}$	res
3249.848	10	30	30761.80	+0.03	22139_2½_— $52901_{3½}^{{}^{\circ}}$	res
3251.262	40*A*	40	30748.42	+0.05	*a*^4^F_2__½_—*z* ${\;}^{6}F_{2½}^{{}^{\circ}}$	res
3254.848	1	4	30714.54	−0.08	20780_4½_—*z* ${\;}^{6}F_{5½}^{{}^{\circ}}$	
3255.012		30	30713.00	−0.05	27273_3½_— $57986_{4½}^{{}^{\circ}}$	5
3255.582		20	30707.62	−0.04	22194_3½_— $52901_{3½}^{{}^{\circ}}$	
3255.824		1	30705.33	+0.06	26227_2½_— $56932_{1½}^{{}^{\circ}}$	
3255.957	60*A*	20	30704.08	−0.06	28187_6__½_— $58891_{1½}^{{}^{\circ}}$	
3257.815		12	30686.57	−0.13	23450_2½_— $54137_{1½}^{{}^{\circ}}$	
3260.32		3	30663.00	−0.03	22139_2½_— $52803_{1½}^{{}^{\circ}}$	
3261.937		8	30647.79	−0.20	26227_2½_— $56874_{2½}^{{}^{\circ}}$	
3262.244	3	60	30644.91	−0.04	28631_3½_— $59276_{3½}^{{}^{\circ}}$	6
3265.988	2	12	30609.78	−0.09	26158_4½_— $56768_{3½}^{{}^{\circ}}$	
3267.430		40	30596.27			
3268.353	–?	30	30587.63	−0.07	24804_3½_— $55392_{4½}^{{}^{\circ}}$	res
3268.882		4	30582.68	−0.16	27273_3½_— $57856_{2½}^{{}^{\circ}}$	
3269.622	50*A*	10	30575.76	+0.02	23450_2½_— $54026_{2½}^{{}^{\circ}}$	
3270.049		3	30571.77	−0.21	23803_3__½_— $54375_{2½}^{{}^{\circ}}$	
3273.300	1	8	30541.41	−0.20	26227_2½_— $56768_{3½}^{{}^{\circ}}$	
3274.788	1	12	30527.53	−0.05	29341_4½_— $59869_{3½}^{{}^{\circ}}$	
3277.608	3	3	30501.27	−0.45	31100_5½_— $61602_{6½}^{{}^{\circ}}$	
3278.148		15	30496.24	−0.21	24991_1½_— $55488_{1½}^{{}^{\circ}}$	7
3278.924	12*A*	20	30489.02	+0.02	31100_5½_— $61589_{5½}^{{}^{\circ}}$	
3282.478		4	30456.01	−0.05	27273_3½_— $57729_{3½}^{{}^{\circ}}$	
3282.693	–?	5	30454.02	+0.02	26158_4½_— $56612_{3½}^{{}^{\circ}}$	
3283.765		20	30444.08			
3285.820	1	15	30425.04	−0.31	34091_4½_— $64516_{4½}^{{}^{\circ}}$	
3286.566	25	75	30418.13	−0.02	*a*^4^F_1½_—z ${\;}^{6}F_{1½}^{{}^{\circ}}$	res
3290.10		1	30385.46	−0.28	26227_2½_— $56612_{3½}^{{}^{\circ}}$	
3291.45		3	30373.00	−0.07	22194_3½_— $52567_{4½}^{{}^{\circ}}$	
3293.138	2	25	30357.43	−0.20	24804_3½_— $55162_{2½}^{{}^{\circ}}$	
3297.418		4	30318.03	−0.11	25169_1½_— $55488_{1½}^{{}^{\circ}}$	
3297.497		4	30317.30	−0.10	26227_2½_— $56544_{2½}^{{}^{\circ}}$	
3298.284		40	30310.07	−0.01	28377_5½_— $58687_{4½}^{{}^{\circ}}$	
3299.39		4	30299.91	−0.03	22503_1½_— $52803_{1½}^{{}^{\circ}}$	
3299.741	6	25	30296.69	−0.06	*a*^4^P_0__½_—z ${\;}^{6}F_{1½}^{{}^{\circ}}$	
3300.344	5	12	30291.15	−0.12	23046_3½_— $53338_{3½}^{{}^{\circ}}$	6
3301.870		8	30277.15	−0.12	*a*^4^D_1½_— $44911_{1½}^{{}^{\circ}}$	
3303.216		6	30264.81	−0.06	20780_4½_— $51045_{3½}^{{}^{\circ}}$	
3303.736		2	30260.05	−0.03	31100_5__½_— $61360_{4½}^{{}^{\circ}}$	
3304.324	4*A*	3	30254.66	−0.24	26158_4½_— $56413_{4½}^{{}^{\circ}}$	
3304.548		80	30252.62	$\{\begin{array}{c} -0.08\\ +0.03 \end{array}$	2380_3½_— $54056_{4½}^{{}^{\circ}}$ 20039_3½_— $50292_{2½}^{{}^{\circ}}$	7
3305.46	1	1	30244.27	+0.07	24918_1½_— $55162_{2½}^{{}^{\circ}}$	
3308.358	3	60	30217.77	+0.06	26158_4½_— $56376_{5½}^{{}^{\circ}}$	5
3310.190		20	30201.05			
3312.163	1–	4	30183.06	−0.03	25209_4½_— $55392_{4½}^{{}^{\circ}}$	
3313.532		4	30170.59	−0.15	24991_1½_— $55162_{2½}^{{}^{\circ}}$	
3314.323		8	30163.39	−0.15	18990_1__½_— $49154_{0½}^{{}^{\circ}}$	
3316.820		3	30140.68	+0.33	31100_5½_— $61240_{5½}^{{}^{\circ}}$	
3317.412	2	15	30135.31	$\{\begin{array}{c} 0.00\\ +0.21 \end{array}$	22139_2½_— $52275_{3½}^{{}^{\circ}}$ 23234_4½_— $53369_{4½}^{{}^{\circ}}$	
3319.488	1	10	30116.47	−0.01	34091_4½_— $64207_{4½}^{{}^{\circ}}$	6
3320.944		10	30103.26	+0.06	23234_4½_— $53338_{3½}^{{}^{\circ}}$	
3322.345		6	30090.57	−0.09	22503_1½_— $52593_{0½}^{{}^{\circ}}$	
3323.414	15	10	30080.89	−0.31	22194_3½_— $52275_{3½}^{{}^{\circ}}$	6
3325.980	2	8	30057.68	−0.10	29341_4½_— $59399_{4½}^{{}^{\circ}}$	
3333.280	1	8	29991.85	−0.05	18990_1½_— $48982_{1½}^{{}^{\circ}}$	
3334.806		5	29978.13	−0.01	27273_3½_— $57252_{2½}^{{}^{\circ}}$	7
3335.404		3	29972.75	+0.27	23450_2½_— $53422_{1½}^{{}^{\circ}}$	
3336.170		2	29965.87	+0.29	19276_2½_— $49242_{2½}^{{}^{\circ}}$	
3337.352		3	29955.26	−0.08	31100_5½_— $61055_{4½}^{{}^{\circ}}$	
3338.276	1	12	29946.97	−0.08	22139_2½_— $52087_{2½}^{{}^{\circ}}$	
3338.632	5	25	29943.78	−0.03	*a* ^4^D_2½_— $44911_{1½}^{{}^{\circ}}$	res
3339.036	5	30	29940.15	−0.14	*a* ^4^G_4½_—*z* ${\;}^{6}F_{4½}^{{}^{\circ}}$	res
3339.590		8	29935.19	−0.06	29341_4½_— $59276_{3½}^{{}^{\circ}}$	
3342.468	25	120	29909.41	+ 0.05	*a* ^4^D_2½_—*z* ${\;}^{6}F_{3½}^{{}^{\circ}}$	res
3343.095	12	60	29903.80	+ 0.04	*a* ^4^G_3½_—*z* ${\;}^{6}F_{4½}^{{}^{\circ}}$	res
3343.410	15	80	29900.99	+ 0.11	*a* ^4^F_4½_— $44758_{4½}^{{}^{\circ}}$	6
3344.898	3	25	29887.69	+0.12	23450_2½_— $53338_{3½}^{{}^{\circ}}$	
3345.858	100*A*	80	29879.11	−0.10	23450_2½_— $53329_{1½}^{{}^{\circ}}$	7
3347.458	1	2	29864.83	−0.23	*a* ^4^F_1½_—*z* ${\;}^{2}S_{0½}^{{}^{\circ}}$	
3348.296	4	80	29857.36	+ 0.06	26227_2½_— $56084_{1½}^{{}^{\circ}}$	7
3348.876	4	40	29852.18	+ 0.13	22503_1½_— $52355_{0½}^{{}^{\circ}}$	6 res
3349.323	3	8	29848.20	+ 0.20	19276_2½_— $49124_{3½}^{{}^{\circ}}$	
3350.634	12	30	29836.52	+ 0.12	20455_1½_— $50292_{2½}^{{}^{\circ}}$	res
3352.388	3	10	29820.91	+ 0.09	*a* ${\;}^{4}D_{1½}^{{}^{\circ}}$— $44455_{0½}^{{}^{\circ}}$	
3352.954	20*A*	15	29815.88	+ 0.03	25672_2½_— $55488_{1½}^{{}^{\circ}}$	*d*
3356.241	4*A*	2	29786.68	+ 0.17	24918_1½_— $54704_{2½}^{{}^{\circ}}$	
3358.292		5	29768.49	−0.01	24991_1½_— $54760_{0½}^{{}^{\circ}}$	
3358.608	50	200	29765.69	−0.04	*a* ^4^G_3½_— $46355_{2½}^{{}^{\circ}}$	4
3359.274		10	29759.79	−0.23	1907_0½_— $48830_{3½}^{{}^{\circ}}$	
3360.326	3	30	29750.47	+ 0.05	19404_0_½— $49154_{0½}^{{}^{\circ}}$	res
3361.104	40*A*	100	29743.58	−0.08	*a* ^4^P_0½_—*z* ${\;}^{2}S_{0½}^{{}^{\circ}}$	res
3361.748		6	29737.89	+ 0.09	28118_2½_— $57856_{2½}^{{}^{\circ}}$	
3362.595		3	29730.39	+ 0.23	*a* ^4^D_3½_—*z* ${\;}^{6}F_{3½}^{{}^{\circ}}$	
3363.721	10	30	29720.44	−0.02	*a* ^4^D_1½_— $44354_{2½}^{{}^{\circ}}$	res
3364.351		4	29714.88	+ 0.02	25045_0_½— $54760_{0½}^{{}^{\circ}}$	
3364.588	1	3	29712.79	−0.26	24991_1½_— $54704_{2½}^{{}^{\circ}}$	
3366.718	2	30	29693.99	+ 0.09	24804_3_½— $54498_{3½}^{{}^{\circ}}$	res
3369.561		3	29668.93	−0.02	22194_3½_— $51863_{3½}^{{}^{\circ}}$	
3369.795	1	4	29666.87	0.00	23234_4½_— $52901_{3½}^{{}^{\circ}}$	
3370.240		3	29662.96	−0.06	23450_2½_— $53113_{2½}^{{}^{\circ}}$	
3372.202	2	15	29645.70	+0.01	30633_4½_— $60278_{4½}^{{}^{\circ}}$	6
3374.866	3	30	29622.30	+0.02	*a* ^4^G_4½_— $46175_{3½}^{{}^{\circ}}$	res
3376.146	30	300	29611.07	−0.01	*a* ^4^D_3½_— $44758_{4½}^{{}^{\circ}}$	res
3379.028	4	40	29585.81	+0.06	*a* ^4^G_3½_— $46175_{3½}^{{}^{\circ}}$	res
3379.825		2	29578.84	+0.06	19404_0½_— $48982_{1½}^{{}^{\circ}}$	
3380.681	1	8	29571.35	+0.20	24804_3½_— $54375_{2½}^{{}^{\circ}}$	
3382.598	50*A*	40	29554.59	+0.41	19276_2½_— $48830_{3½}^{{}^{\circ}}$	
3383.091	2	50	29550.28	+0.10	29341_4½_— $58891_{5½}^{{}^{\circ}}$	
3384.888	25	20	29534.60	−0.14	25169_1½_— $54704_{2½}^{{}^{\circ}}$	
3386.502	4	20	29520.52	+0.17	23046_2½_— $52567_{4½}^{{}^{\circ}}$	
3386.933		3	29516.76	+0.16	28491_1½_— $58007_{1½}^{{}^{\circ}}$	
3387.634	1	15	29510.66	+0.40	26929_5½_— $56439_{6½}^{{}^{\circ}}$	
3389.544		2	29494.03	−0.01	24991_1½_— $54485_{0½}^{{}^{\circ}}$	
3389.985		1	29490.19	+0.05	25672_2½_— $55162_{2½}^{{}^{\circ}}$	
3390.324	1	20	29487.24	+0.10	19637_2½_— $49124_{3½}^{{}^{\circ}}$	res
3394.450	2	15	29451.40	+0.16	23450_2½_— $52901_{3½}^{{}^{\circ}}$	
3394.907	1	3	29447.44	+0.33	26929_5½_— $56376_{5½}^{{}^{\circ}}$	
3395.712		2	29440.45	+0.05	25045_0½_— $54485_{0½}^{{}^{\circ}}$	
3398.690		1	29414.66	+0.09	23955_5½_— $53369_{4½}^{{}^{\circ}}$	
3398.922	15	120	29412.65	+0.02	1800_3½_— $47413_{2½}^{{}^{\circ}}$	7
3401.884	30	300	29387.04	+0.04	*a* ^4^D_2½_— $44354_{2½}^{{}^{\circ}}$	res
3402.194		40?	29384.37	+0.11	24991_1½_— $54375_{2½}^{{}^{\circ}}$	
3404.082		1	29368.07	+0.07	29341_4½_— $58709_{3½}^{{}^{\circ}}$	
3406.086	2	12	29350.79	+0.09	25672_2½_— $55022_{3½}^{{}^{\circ}}$	
3406.588	1	20	29346.47	+0.15	29341_4½_— $58687_{4½}^{{}^{\circ}}$	
3406.824	*30A*	40	29344.43	+0.14	*a* ^4^P_1½_— $39936_{2½}^{{}^{\circ}}$	
3407.450	2	10	29339.04	+0.16	2727_3½_— $56612_{3½}^{{}^{\circ}}$	
3408.214	2	5	29332.47	+0.19	2323_4½_— $52567_{4½}^{{}^{\circ}}$	
3410.170	1	25	29315.64	−0.09	25169_1½_— $54485_{0½}^{{}^{\circ}}$	
3412.736	3	30	29293.60	+0.08	18990_1½_— $48284_{2½}^{{}^{\circ}}$	res
3415.406	4	6	29270.70	+0.16	27273_3½_— $56544_{2½}^{{}^{\circ}}$	
3416.370		2	29262.44	+0.39	19070_4½_— $48332_{5½}^{{}^{\circ}}$	
3416.623	20	100	29260.27			
3417.620		3	29251.74	−0.13	24804_3½_— $54056_{4½}^{{}^{\circ}}$	
3419.720		5	29233.77	+0.14	26158_4½_— $55392_{4½}^{{}^{\circ}}$	
3421.134	4	40	29221.69	+0.12	24804_3½_— $54026_{2½}^{{}^{\circ}}$	4
3421.430		5	29219.17	+0.07	24918_1½_— $54137_{1½}^{{}^{\circ}}$	
3422.742	1	6	29207.97	+0.17	*a* ^4^D_3½_— $44354_{2½}^{{}^{\circ}}$	
3424.436	5	50	29193.52	+0.20	19637_2½_— $48830_{3½}^{{}^{\circ}}$	res
3426.20		4	29178.5	+0.3	31100_5½_— $60278_{4½}^{{}^{\circ}}$	
3430.558		1	29141.42	+0.12	20039_3½_— $49181_{4½}^{{}^{\circ}}$	
3430.739	1–	8	29139.89	+0.11	27273_3½_— $56413_{4½}^{{}^{\circ}}$	
3435.636		4	29098.35	+0.29	28631_3½_— $57729_{3½}^{{}^{\circ}}$	
3435.710	30*A*	6	29097.73	−0.17	23803_3½_— $52901_{3½}^{{}^{\circ}}$	*d*
3437.233	3	20?	29084.83	+0.05	20039_3½_— $49124_{3½}^{{}^{\circ}}$	res
3440.583	4	20	29056.51	+0.10	*a* ^4^G_5½_—*z* ${\;}^{6}F_{4½}^{{}^{\circ}}$	
3440.644		200	29056.00	+0.06	33910_5½_— $62966_{5½}^{{}^{\circ}}$	
3442.493	2	20	29040.40	+0.18	23046_3½_— $52087_{2½}^{{}^{\circ}}$	res
3443.167		2	29034.71	+0.03	24991_1½_— $54026_{2½}^{{}^{\circ}}$	
3444.934		2	29019.82	+0.04	25209_4½_— $54229_{5½}^{{}^{\circ}}$	
3449.862	10	100	28978.36	+0.05	*a* ^4^F_3½_— $42390_{3½}^{{}^{\circ}}$	6
3451.166		10	28967.41	+0.08	25169_1½_— $54137_{1½}^{{}^{\circ}}$	7
3452.494	8	30	28956.27	+0.10	*a* ^4^P2½— $42390_{3½}^{{}^{\circ}}$	res
3454.962	} 1*d*	30*d*	$\left\{ \begin{array}{l} 28935.54\\ 28935.14 \end{array}\right.$	+0.24	26227_2½_— $55162_{2½}^{{}^{\circ}}$	
3455.015				+0.17	22503_1_½— $51438_{2½}^{{}^{\circ}}$	5
3460.377	1	10	28890.31	+ 0.12	19442_6½_— $48332_{5½}^{{}^{\circ}}$	
3463.510	20	150	28864.18	+ 0.08	*a* ^4^P_2½_— $42298_{1½}^{{}^{\circ}}$	*7*
3464.446	1	20	28856.38	+ 0.01	25169_1½_— $54026_{2½}^{{}^{\circ}}$	res
3465.062	1	10	28851.25	+ 0.08	22194_3½_— $51045_{3½}^{{}^{\circ}}$	6
3468.225	4	6	28824.94	+ 0.16	23450_2½_— $52275_{3½}^{{}^{\circ}}$	
3469.266	2	25	28816.29	+ 0.60	23046_3½_— $51863_{3½}^{{}^{\circ}}$	6
3470.746		1	28804.CO	0.00	33910_5½_— $62714_{6½}^{{}^{\circ}}$	
3471.230	1	6	28799.99	+ 0.15	26158_4½_— $54958_{5½}^{{}^{\circ}}$	
3471.698		3	28796.10	+ 0.24	26227_2½_— $55022_{3½}^{{}^{\circ}}$	
3472.310	1	10	28791.03	+ 0.07	20039_3½_— $48830_{3½}^{{}^{\circ}}$	
3472.896		4	28786.17	0.00	20455_1½_— $49242_{2½}^{{}^{\circ}}$	
3475.288	2	80	28766.36	+ 0.04	30633_4½_— $59399_{4½}^{{}^{\circ}}$	res
3475.936	1	10	28761.00	0.00	28491_1½_— $57252_{2½}^{{}^{\circ}}$	
3476.496	30*A*	8	28756.36	+ 0.27	28118_2½_— $56874_{2½}^{{}^{\circ}}$	7
3477.092	1	4	28751.43	+ 0.09	22503_1½_— $51254_{1½}^{{}^{\circ}}$	6
3481.910	–?	10	28711.65	−0.01	28377_5½_— $57089_{4½}^{{}^{\circ}}$	
3483.490		3	28698.63	+ 0.06	20455_1½_— $49154_{0½}^{{}^{\circ}}$	
3486.122	10	50	28676.96	+ 0.17	*a* ^4^G_2½_— $44911_{1½}^{{}^{\circ}}$	res
3487.080		3	28669.08	+ 0.11	22194_3½_— $50863_{4½}^{{}^{\circ}}$	
3489.42		3	28649.86	+ 0.15	28118_2½_— $56768_{3½}^{{}^{\circ}}$	
3489.785	1	3	28646.86	+ 0.17	20534_5½_— $45181_{4½}^{{}^{\circ}}$	
3490.313	8	40	28642.53	+ 0.19	*a* ^4^G_2½_—*z* ${\;}^{6}F_{3½}^{{}^{\circ}}$	res
3490.912	12	50	28637.61	+ 0.12	*a* ^4^F_3½_—*z* ${\;}^{6}F_{2½}^{{}^{\circ}}$	4
3491.127	8	25	28635.85	+ 0.12	*a* ^4^F_2½_— $39936_{2½}^{{}^{\circ}}$	6 res
3492.056	3	40	28628.23	+ 0.07	23234_4½_— $51863_{3½}^{{}^{\circ}}$	
3492.46	1	3	28624.92	−0.04	34091_4½_— $62715_{4½}^{{}^{\circ}}$	
3493.62	1–		28615.42	+ 0.07	*a* ^4^P_4½_—*z* $F_{2½}^{{}^{\circ}}$	
3498.134	1	5	28578.49			
3503.22		3	28537.00	+ 0.11	*a* ^4^P_1½_—*z* ${\;}^{6}F_{1½}^{{}^{\circ}}$	
3503.656		30	28533.45	+ 0.05	24804_3½_— $53338_{3½}^{{}^{\circ}}$	
3505.05		2	28522.1	0.0	24918_1½_— $53440_{0½}^{{}^{\circ}}$	
3507.17		2	28504.87	−0.01	24918_1½_— $53422_{1½}^{{}^{\circ}}$	
3508.54	1	3	28493.73	−0.11	28118_2½_— $56612_{3½}^{{}^{\circ}}$	
3508.664		50	28492.73	0.00	18000_3½_—*z* ${\;}^{6}F_{4½}^{{}^{\circ}}$	
3511.269	2	15	28471.59	+ 0.15	23803_3½_— $52275_{3½}^{{}^{\circ}}$	
3512.077	1	20	28465.04	0.00	25672_2½_— $54137_{1½}^{{}^{\circ}}$	res
3512.98		3	28457.72	+ 0.12	28631_3½_— $57089_{4½}^{{}^{\circ}}$	
3514.106		20	28448.60	−0.01	24991_1½_— $53440_{0½}^{{}^{\circ}}$	
3514.990	1	4	28441.45	+ 0.18	28491_1½_— $56932_{1½}^{{}^{\circ}}$	
3516.234		15	28431.39	−0.03	24991_1½_— $53422_{1½}^{{}^{\circ}}$	
3517.39		3	28422.04	−0.33	18990_1½_— $47413_{2½}^{{}^{\circ}}$	
3518.56	1	2	28412.60	+ 0.07	23450_2½_— $51863_{3½}^{{}^{\circ}}$	
3521.57		2	28388.31	−0.05	29341_4½_— $57729_{3½}^{{}^{\circ}}$	
3522.102	1	10	28384.02	+ 0.03	28491_1½_— $56874_{2½}^{{}^{\circ}}$	
3523.133		8	28375.71			
3525.733	3	30	28354.79	+ 0.09	18000_3½_— $46355_{2½}^{{}^{\circ}}$	res
3526.817	–	50	28346.08	+ 0.06	34091_4½_— $62437_{4½}^{{}^{\circ}}$	6
3527.040	–?	30	28344.28	+ 0.14	20780_4½_— $49124_{3½}^{{}^{\circ}}$	
3529.546	10	100	28324.16	+ 0.12	*a* ^4^G_4½_—*z* ${\;}^{6}F_{3½}^{{}^{\circ}}$	res
3531.440	2	5	28308.97	+ 0.12	24804_3½_— $53113_{2½}^{{}^{\circ}}$	
3532.680		3	28299.03	+ 0.15	31100_5½_— $59399_{4½}^{{}^{\circ}}$	
3534.12		5	28287.50	−0.01	*a* ^4^G_3½_—*z* ${\;}^{6}F_{3½}^{{}^{\circ}}$	
3534.498	3	25	28284.48	−0.03	25045_0½_— $53329_{1½}^{{}^{\circ}}$	6?
3536.12		5	28271.50	−0.07	26227_2½_— $54498_{3½}^{{}^{\circ}}$	
3536.268	4	80	28270.32	+ 0.02	25169_1½_— $53440_{0½}^{{}^{\circ}}$	6?
3538.40		2	28253.29	+ 0.18	25169_1½_— $53422_{1½}^{{}^{\circ}}$	
3538.55	(—?)	3	28252.09	+ 0.09	28187_6½_— $56439_{6½}^{{}^{\circ}}$	
3539.455	3*A*	4	28244.87	+0.13	20039_3½_— $48284_{2½}^{{}^{\circ}}$	res
3544.460	5	25	28204.98	+ 0.02	a ^4^G_4½_— $44758_{4½}^{{}^{\circ}}$	res
3546.474	3*A*	3	28188.97	−0.01	18990_1½_— $47179_{1½}^{{}^{\circ}}$	
3547.00	1		28184.79	+ 0.23	19404_0½_— $47588_{1½}^{{}^{\circ}}$	
3548.258	15*A*	12	28174.79	+0.07	18000_3½_— $46175_{3½}^{{}^{\circ}}$	*d*
3549.052	25	150	28168.49	+0.06	*a* ^4^G_3½_— $44758_{4½}^{{}^{\circ}}$	res
3550.14		3	28159.86	+ 0.02	25169_1½_— $53829_{1½}^{{}^{\circ}}$	
3551.528		10	28148.85	+ 0.03	26227_2½_— $54375_{2½}^{{}^{\circ}}$	
3553.020		5	28137.03	+ 0.22	19276_2½_— $47413_{2½}^{{}^{\circ}}$	
3554.068	1	3	28128.74	−0.05	25209_4½_— $53338_{3½}^{{}^{\circ}}$	
3554.93		2	28121.92	−0.04	24991_1½_— $53113_{2½}^{{}^{\circ}}$	
3555.166	12	120	28120.05	+0.07	*a* ^4^G_2½_— $44354_{2½}^{{}^{\circ}}$	res
3557.914	5	30	28098.33	+0.08	22194_3½_— $50292_{2½}^{{}^{\circ}}$	
3560.67		2	28076.58	+0.04	30633_4½_— $58709_{3½}^{{}^{\circ}}$	
3561.456		5	28070.39	+ 0.07	26158_4½_— $54229_{5½}^{{}^{\circ}}$	
3562.508	1	5	28062.10	+0.30	28377_5½_— $56439_{6½}^{{}^{\circ}}$	4
3563.59		3	28053.6	+0.2	28491_1½_— $56544_{2½}^{{}^{\circ}}$	
3563.998	1	2	28050.37	+0.05	20780_4½_— $43830_{3½}^{{}^{\circ}}$	
3565.834	1	3	28035.92	+0.08	28377_5½_— $56413_{4½}^{{}^{\circ}}$	
3566.73		3	28028.9	−0.3	26929_5½_— $54958_{5½}^{{}^{\circ}}$	
3571.972	3	8	27987.75	+ 0.22	23450_2½_— $51438_{2½}^{{}^{\circ}}$	
3572.474	60	200	27983.82	+0.02	*a* ^4^P_1½_—*z* ${\;}^{2}S_{0½}^{{}^{\circ}}$	res
3572.830	1	5	27981.03	+ 0.15	28631_3½_— $56612_{3½}^{{}^{\circ}}$	6
3574.82		2	27965.4	0.0	28118_2½_— $56084_{1½}^{{}^{\circ}}$	
3577.59		2	27943.8	+0.2	25169_1½_— $53113_{2½}^{{}^{\circ}}$	
3579.64		3	27927.8	−0.1	22503_1½_— $50430_{1½}^{{}^{\circ}}$	
3581.876	2	6	27910.36	+0.16	26227_2½_— $54137_{1½}^{{}^{\circ}}$	res
3582.75	1	2	27903.56	+ 0.14	19276_2½_— $47179_{1½}^{{}^{\circ}}$	
3583.458	1	8	27898.04	+0.24	26158_4½_— $54056_{4½}^{{}^{\circ}}$	6
3584.68		3	27888.53	+0.09	27273_3½_— $55162_{2½}^{{}^{\circ}}$	
3592.418	50	150	27828.46	$\{\;\begin{array}{c} -0.09\\ +0.13 \end{array}$	20455_1½_— $48284_{2½}^{{}^{\circ}}$ *a* ^4^F_2½_—*z* ${\;}^{6}F_{1½}^{{}^{\circ}}$	5 res 4
3596.171	2	30	27799.42	+0.18	26227_2½_— $54026_{2½}^{{}^{\circ}}$	
3597.48	–?	2	27789.31	+0.04	22503_1½_— $50292_{2½}^{{}^{\circ}}$	
3598.45	2	20	27781.8	0.0	28631_3½_— $56413_{4½}^{{}^{\circ}}$	7
3600.594		1	27765.27	+0.12	*a* ^4^G_3½_— $44354_{2½}^{{}^{\circ}}$	
3600.933	1	6	27762.66	+0.18	24804_3½_— $52567_{4½}^{{}^{\circ}}$	
3601.55		1	27757.90	+0.10	25045_0½_— $52803_{1½}^{{}^{\circ}}$	
3602.455	3	8	27750.93	+ 0.11	25672_2½_— $53422_{1½}^{{}^{\circ}}$	
3602.700	–?	1	27749.04	+0.04	27273_3½_— $55022_{3½}^{{}^{\circ}}$	
3608.280		10	27706.13			
3610.044	1	20	27692.59	+0.13	25209_4½_— $52901_{3½}^{{}^{\circ}}$	res
3611.826	1	20	27678.92	+0.02	33910_5½_— $61589_{5½}^{{}^{\circ}}$	6
3613.530		4	27665.88	−0.03	25672_2½_— $53338_{3½}^{{}^{\circ}}$	
3613.785	30	150	27663.93	+ 0.09	*a* ^4^D_1½_— $43298_{1½}^{{}^{\circ}}$	res
3614.606	1	8	27657.64	+0.09	2567_2½_**—** $53329_{1½}^{{}^{\circ}}$	
3617.652		4	27634.36	$\{\;\begin{array}{c} +0.05\\ +0.17 \end{array}$	18990_1½_— $46625_{0½}^{{}^{\circ}}$ 23803_3½_— $51438_{2½}^{{}^{\circ}}$	
3618.434	1	25	27628.39	+0.21	23234_4½_— $50863_{4½}^{{}^{\circ}}$	
3628.413	3	30	27552.40	+0.05	20780_4½_— $48332_{5½}^{{}^{\circ}}$	res
3628.924	2	25	27548.52	0.00	25045_0½_— $52593_{0½}^{{}^{\circ}}$	res
3630.080	2	3	27539.75	0.15	23955_5½_**—***z* ${\;}^{6}F_{5½}^{{}^{\circ}}$	
3630.950		8	27533.15	+0.10	*a* ^4^F_4½_— $42390_{3½}^{{}^{\circ}}$	
3639.213		1	27470.64	+ 0.03	24804_3½_— $52275_{3½}^{{}^{\circ}}$	
3641.403	60	120	27454.12	+0.03	*a* ^4^F_3½_—*z* ${\;}^{6}F_{0½}^{{}^{\circ}}$	7
3645.594	10	100	27422.56	+0.11	*a* ^4^D_2½_— $42390_{3½}^{{}^{\circ}}$	res
3646.577	10	50	27415.16	+0.07	*a* ^4^D_1½_—*z* ${\;}^{6}F_{2½}^{{}^{\circ}}$	
3652.114	10	20	27373.60	+ 0.01	20039_3½_— $47413_{2½}^{{}^{\circ}}$	
3653.324	2	10	27364.54	+0.10	18990_1½_— $46355_{2½}^{{}^{\circ}}$	
3654.74		10	27353.94	+0.04	30633_4½_— $57986_{4½}^{{}^{\circ}}$	
3657.583	40	120	27332.67	−0.02	*a* ^4^P_0½_—z ${\;}^{6}F_{0½}^{{}^{\circ}}$	
3657.871	10	40	27330.52	+0.14	*a* ^4^D_2½_— $42298_{1½}^{{}^{\circ}}$	res
3660.604	30*A*	8	27310.12	+0.21	25045_0½_— $52355_{0½}^{{}^{\circ}}$	
3670.65		1?	27235.37	+0.10	34091_4½_— $61326_{3½}^{{}^{\circ}}$	
3672.547	5	25	27221.31	+0.12	19404_0½_— $46625_{0½}^{{}^{\circ}}$	6
3677.405	1	8	27185.35	+0.11	25169_1½_— $52355_{0½}^{{}^{\circ}}$	
3678.210	1	10	27179.40	+0.07	26158_4½_— $53338_{3½}^{{}^{\circ}}$	res
3687.510		2	27110.85	−0.22	26227_2½_— $53338_{3½}^{{}^{\circ}}$	
3689.596		10	27095.52	+0.06	24991_1½_— $52087_{2½}^{{}^{\circ}}$	
3691.463	1	2	27081.82	+0.19	*a* ^4^D_2½_—*z* ${\;}^{6}F_{2½}^{{}^{\circ}}$	
3691.846	4	40	27079.01	+0.13	19276_2½_— $46355_{2½}^{{}^{\circ}}$	res
3692.864		2	27071.55	−0.53	29341_4½_— $66413_{4½}^{{}^{\circ}}$	
3694.525	40*A*	40	27059.38	+0.17	23803_3½_— $50863_{4½}^{{}^{\circ}}$	5
3705.310	1	4	26980.62	+0.17	23450_2½_— $50430_{1½}^{{}^{\circ}}$	
3707.478	3	2	26964.84	+0.06	34091_4½_— $61055_{4½}^{{}^{\circ}}$	
3708.494	8	60	26957.45	+0.05	20455_1½_— $47413_{2½}^{{}^{\circ}}$	
3712.202	3	12	26930.53	+0.09	22194_3½_— $49124_{3½}^{{}^{\circ}}$	
3714.033		2	26917.25	+0.10	25169_1½_— $52087_{3½}^{{}^{\circ}}$	
3715.832	3	8	26904.22	+0.26	28118_2½_— $55022_{3½}^{{}^{\circ}}$	
3716.062	10	50	26902.55	+0.12	*a* ^4^D_3½_—*z* ${\;}^{6}F_{2½}^{{}^{\circ}}$	res
3716.550	2	2	26899.02	+0.12	19276_2½_— $46175_{3½}^{{}^{\circ}}$	
3719.66		3	26876.53	+0.05	18000_3½_—*z* ${\;}^{6}F_{3½}^{{}^{\circ}}$	
3721.102		10	26866.12			
3732.684	2	2	26782.76	+0.08	27273_3½_— $54056_{4½}^{{}^{\circ}}$	
3736.213	12	60	26757.46	+0.06	18000_3½_— $44758_{4½}^{{}^{\circ}}$	res
3736.91		2	26752.5	+0.1	27273_3½_— $54026_{2½}^{{}^{\circ}}$	
3741.706	30*A*	12	26718.18	+0.16	19637_2½_— $46355_{2½}^{{}^{\circ}}$	6
3745.530	4	40	26690.90	+0.17	22139_2½_— $43830_{3½}^{{}^{\circ}}$	7
3750.744	1	8	26653.80	+0.05	25209_4½_— $51863_{3½}^{{}^{\circ}}$	
3753.162		3	26636.63	+0.01	22194_3½_— $48830_{3½}^{{}^{\circ}}$	
3753.620		10	26633.38	+0.02	24804_3½_— $51438_{2½}^{{}^{\circ}}$	
3760.364	2	20	26585.61	−0.10	28118_2½_— $54704_{2½}^{{}^{\circ}}$	
3763.616	2	2	26562.64	−0.10	18990_1½_— $45553_{1½}^{{}^{\circ}}$	
3768.97		4	26524.91	+0.06	*a* ^4^F_3½_— $39936_{2½}^{{}^{\circ}}$	
3772.05	8*A*	3	26503.25	+0.54	*a* ^4^P_2½_— $39936_{2½}^{{}^{\circ}}$	
3774, 14	–	20	26488.58	+0.09	23803_3½_— $50292_{2½}^{{}^{\circ}}$	
3777.35	1	10	26466.07	+0.01	18990_1½_— $45457_{0½}^{{}^{\circ}}$	
3778.686	30*A*	8	26456.71	+0.27	30633_4½_— $57089_{4½}^{{}^{\circ}}$	
3785.58		20	26408.5	+0.1	26158_4½_— $52567_{4½}^{{}^{\circ}}$	
3789.726		20	26379.64	−0.03	28118_2½_— $54498_{3½}^{{}^{\circ}}$	
3804.481	1	2	26277.3	+0.1	19276_2½_— $45553_{1½}^{{}^{\circ}}$	
3814.418	5	6	26208.88	−0.32	25045_0½_— $51354_{1½}^{{}^{\circ}}$	
3822.198	2	4	26155.53	+0.10	*a* ^4^C_2½_— $42390_{3½}^{{}^{\circ}}$	
3823.062	3	10	26149.62	0.00	19404_6½_— $45553_{1½}^{{}^{\circ}}$	res
3827.89		4	26116.6	+0.1	26158_4½_— $52275_{3½}^{{}^{\circ}}$	
3833.61	2		26077.7	0.0	23046_3½_— $49124_{3½}^{{}^{\circ}}$	
3837.238	4	10	26053.02	+0.08	19404_0½_— $45457_{0½}^{{}^{\circ}}$	
3846.688	2		25989.02	+0.02	31100_5½_— $57089_{4½}^{{}^{\circ}}$	
3851.131	–?	2	25959.03	−0.05	20534_5½_—*z* ${\;}^{6}F_{4½}^{{}^{\circ}}$	
3851.570	12	60	25956.07	+0.04	*a* ^4^D_0½_—*z* ${\;}^{6}F_{1½}^{{}^{\circ}}$	res
3856.828	3	6	25920.69	+0.02	18990_1½_— $44911_{1½}^{{}^{\circ}}$	6
3859.986	2	6	25899.48	+0.01	20455_1½_— $48355_{2½}^{{}^{\circ}}$	
3872.656	4	10	25814.75	+0.14	*a* ^4^G_2½_—*z* ${\;}^{6}F_{2½}^{{}^{\circ}}$	
3877.29	2	15	25783.9	0.0	23046_3½_— $48830_{3½}^{{}^{\circ}}$	6
3877.65	1		25781.5	+0.1	22503_1½_— $48284_{2½}^{{}^{\circ}}$	
3883.336	2	3	25743.76	−0.20	28631_3½_— $54375_{2½}^{{}^{\circ}}$	
3887.951	1	3	25713.20	+0.15	20780_4½_—*z* ${\;}^{6}F_{4½}^{{}^{\circ}}$	
3890.658	–?	2	25695.31	0.00	*a* ^4^P_2½_—*z* ${\;}^{6}F_{1½}^{{}^{\circ}}$	
3891.83	1	1	25687.57	+0.15	19070_4½_— $44758_{4½}^{{}^{\circ}}$	
3896.930	3	20	25653.95	+0.18	25209_4½_— $50863_{4½}^{{}^{\circ}}$	6
3899.782	2	3	25635.19	+0.08	19276_2½_— $44911_{1½}^{{}^{\circ}}$	
3900.879	2	8	25627.98	+0.10	27273_3½_— $52901_{3½}^{{}^{\circ}}$	
3905.79	1	3	25595.8	0.0	23234_4½_— $48830_{3½}^{{}^{\circ}}$	
3909.276	2	2	25572.94	+0.11	*a* ^4^P_1½_—*z* ${\;}^{6}F_{0½}^{{}^{\circ}}$	
3915.456	2	5	25532.57	+0.21	23450_2½_— $48982_{1½}^{{}^{\circ}}$	
3935.43	4	10	25403.0	+0.1	*a* ^4^D_0½_—*z* ${\;}^{2}S_{0½}^{{}^{\circ}}$	
3936.653	1	5	25395.10	+0.06	20780_4½_— $46175_{3½}^{{}^{\circ}}$	
3936.782	1		25394.27	−0.11	28631_3½_— $54026_{2½}^{{}^{\circ}}$	
3938.10		1	25385.8	0.0	25045_0½_— $50460_{1½}^{{}^{\circ}}$	
3939.43	8	3	25377.2	0.0	23803_3½_— $49181_{4½}^{{}^{\circ}}$	
3951.067	3	6	25302.45	0.00	*a* ^4^D_1½_— $39936_{2½}^{{}^{\circ}}$	
3955.606	1	1	25273.42	+0.06	22139_2½_— $47413_{2½}^{{}^{\circ}}$	
3960.87	2	6	25239.83	+0.03	19637_2½_—*z* ${\;}^{6}F_{3½}^{{}^{\circ}}$	
3691.192	–?	3	25237.78	+0.10	23046_3½_— $48284_{2½}^{{}^{\circ}}$	
3964.096	–?	6	25219.29	+0.04	22194_3½_— $47413_{2½}^{{}^{\circ}}$	
3983.292	50*A*	5	25097.76	−0.01	20455_1½_— $45553_{2½}^{{}^{\circ}}$	6, *d*
3986.38		1	25078.3	0.0	19276_2½_— $44354_{2½}^{{}^{\circ}}$	
3990.42		2	25052.9	+0.1	22535_0½_— $47588_{1½}^{{}^{\circ}}$	
3994.56	2		25027.0	+0.1	23803_3½_— $48830_{3½}^{{}^{\circ}}$	
4000.10	1	1	24992.3	+0.1	28377_5½_— $53369_{4½}^{{}^{\circ}}$	
4001.883	1	4	24981.17	−0.01	33910_5½_— $58891_{5½}^{{}^{\circ}}$	6
4060.166	1	2	24622.58	+0.06	28491_1½_— $53113_{2½}^{{}^{\circ}}$	
4069.61	1		24565.4	−0.3	26929_5½_—*z* ${\;}^{6}F_{5½}^{{}^{\circ}}$	
4081.294	4	10	24495.12	+0.07	*a* ^4^D_1½_—*z* ${\;}^{6}F_{1½}^{{}^{\circ}}$	6
4114.19	1	2	24299.3	−0.08	22194_3½_—*z* ${\;}^{6}F_{4½}^{{}^{\circ}}$	
4144.41	1		24122.1	−0.1	22503_1½_— $46625_{0½}^{{}^{\circ}}$	
4146.62	–?	1	24109.2	−0.1	25045_0½_— $49154_{0½}^{{}^{\circ}}$	
4157.030	2	5	24048.86	+0.11	18000_3½_—*z* ${\;}^{6}F_{2½}^{{}^{\circ}}$	
4165.60	1	2	23999.4	+0.2	20455_1½_— $44455_{0½}^{{}^{\circ}}$	
4175.58	5	5	23942.0	0.0	*a* ^4^D_1½_—*z* ${\;}^{2}S_{0½}^{{}^{\circ}}$	
4257.82	1		23479.6	−0.2	24804_3½_— $48284_{2½}^{{}^{\circ}}$	
4262.75	1		23452.4	0.0	25672_2½_— $49124_{3½}^{{}^{\circ}}$	
4263.81	1		23446.6	0.0	23046_3½_—*z* ${\;}^{6}F_{4½}^{{}^{\circ}}$	
4289.28	2	2	23307.4	$\left\{ \begin{array}{r} 0.0\\ +0.2 \end{array}\right.$	28187_6½_—*z* ${\;}^{6}F_{5½}^{{}^{\circ}}$ 18990_1½_— $42298_{1½}^{{}^{\circ}}$	
4298.26	1		23258.7	+0.1	23234_4½_—*z* ${\;}^{6}F_{4½}^{{}^{\circ}}$	
4323.392	1	6	23123.49	+0.04	25209_4½_— $48332_{5½}^{{}^{\circ}}$	
4335.58	1?	6	23058.5	0.0	18990_1½_—*z* ${\;}^{6}F_{2½}^{{}^{\circ}}$	
4342.40		2	23022.3	0.0	26158_4½_— $49181_{4½}^{{}^{\circ}}$	
4343.20	2	5	23018.0	+0.1	22535_0½_— $45553_{1½}^{{}^{\circ}}$	res
4348.108	20A	25	22992.05	+0.08	*a* ^4^D_0½_—*z* ${\;}^{6}F_{0½}^{{}^{\circ}}$	res
4361.534	8A	3	22921.28	+0.06	22535_0½_— $45457_{0½}^{{}^{\circ}}$	
4393.80	1	2	22753.0	+0.1	19637_2½_— $42390_{3½}^{{}^{\circ}}$	
4396.88	2	2	22737.0	$\left\{ \begin{array}{r} -0.2\\ 0.0 \end{array}\right.$	22139_2½_—*z* ${\;}^{6}F_{3½}^{{}^{\circ}}$ 30633_4½_— $53369_{4½}^{{}^{\circ}}$	
4421.836	15*A*	3	22608.69	+0.03	24804_3½_— $47413_{2½}^{{}^{\circ}}$	
4438.92		2	22521.7	0.0	34091_4½_— $56612_{3½}^{{}^{\circ}}$	
4468.72		1	22371.5	−0.1	23803_3½_— $46175_{3½}^{{}^{\circ}}$	
4508.50		2	22174.1	+0.1	26158_4½_— $48332_{5½}^{{}^{\circ}}$	
4542.144	1	2	22009.86	$\left\{ \begin{array}{r} -0.21\\ +0.15 \end{array}\right.$	25169_1½_— $47179_{1½}^{{}^{\circ}}$ 20039_3½_—*z* ${\;}^{6}F_{2½}^{{}^{\circ}}$	
4554.045	1	2	21952.35	+0.23	22503_1½_— $44455_{0½}^{{}^{\circ}}$	
4604.576	1	3	21711.44	+0.14	23046_3½_— $44758_{4½}^{{}^{\circ}}$	
4658.24	2	2	21461.3	+0.19	23450_2½_— $44911_{1½}^{{}^{\circ}}$	
4665.76	4*A*	3	21426.74	+0.06	23450_2½_—*z* ${\;}^{6}F_{3½}^{{}^{\circ}}$	
4691.74	1	2	21308.1	+0.1	23046_3½_— $44354_{2½}^{{}^{\circ}}$	
4718.91	1	3	21185.4	−0.1	25169_1½_— $46355_{2½}^{{}^{\circ}}$	
4768.25	1	2	20966.2	+0.1	25209_4½_— $46175_{3½}^{{}^{\circ}}$	
4782.35	1	1	20904.4	+0.1	23450_2½_— $44354_{2½}^{{}^{\circ}}$	
4864.565	2	3	20551.08	+0.10	23803_3½_— $44354_{2½}^{{}^{\circ}}$	
4959.33	3	2	20158.4	+0.2	22139_2½_— $42298_{1½}^{{}^{\circ}}$	
5021.324	5	2	19909.51	+0.03	22139_2½_—*z* ${\;}^{6}F_{2½}^{{}^{\circ}}$	
5104.427	6	6	19585.37	+0.01	18990_1½_—*z* ${\;}^{2}S_{0½}^{{}^{\circ}}$	
5114.59	3	3	19546.46	+0.07	22503_1½_—*z* ${\;}^{6}F_{2½}^{{}^{\circ}}$	
5131.83	2	2	19480.8	−0.1	20455_1½_— $39936_{2½}^{{}^{\circ}}$	
5214.41	2	?	19172.3	+0.1	19404_0½_—*z* ${\;}^{2}S_{0½}^{{}^{\circ}}$	
5219.04	3	?	19155.3	−0.1	23234_1½_— $42390_{3½}^{{}^{\circ}}$	
5260.92	4	4	19002.8	+0.2	23046_3½_—*z* ${\;}^{6}F_{2½}^{{}^{\circ}}$	
5278.38	8	2	18939.9	+0.1	23450_2½_— $42390_{3½}^{{}^{\circ}}$	
5304.17	10	4	18847.8	+0.1	23450_2½_— $42298_{1½}^{{}^{\circ}}$	
5353.677	5	3	18673.54	+0.06	20455_1½_—*z* ${\;}^{6}F_{1½}^{{}^{\circ}}$	
5375.103	3	2	18599.12	$\left\{ \begin{array}{r} -0.24\\ +0.17 \end{array}\right.$	26158_4½_— $44758_{4½}^{{}^{\circ}}$ 23450_2½_—*z* ${\;}^{6}F_{2½}^{{}^{\circ}}$	
5410.89	2		18476.12	−0.01	34091_4½_— $52567_{4½}^{{}^{\circ}}$	
5597.056	3	3	17861.63	+0.06	28631_3½_—*z* ${\;}^{6}F_{4½}^{{}^{\circ}}$	
5698.47	1	1	17543.7	+0.1	28631_3½_— $46175_{3½}^{{}^{\circ}}$	
5821.00	4*A*	1	17174.42	+0.03	18990_1½_—*z* ${\;}^{6}F_{0½}^{{}^{\circ}}$	
5884.32	2	2	16989.6	+0.2	22139_2½_—*z* ${\;}^{6}F_{1½}^{{}^{\circ}}$	
5964.48	3	5	16761.28	+0.01	19404_0½_—*z* ${\;}^{6}F_{0½}^{{}^{\circ}}$	
6024.64	2	5	16593.91	+0.30	22535_0½_—*z* ${\;}^{6}F_{1½}^{{}^{\circ}}$	
6219.77	2	8	16073.32	+0.06	22503_1½_—*z* ${\;}^{2}S_{0½}^{{}^{\circ}}$	

## Footnotes

2 Figures in brackets indicate the literature references at the end of this paper.
